# YSIRK-G/S-directed translocation is required for *Streptococcus suis* to deliver diverse cell wall anchoring effectors contributing to bacterial pathogenicity

**DOI:** 10.1080/21505594.2020.1838740

**Published:** 2020-11-02

**Authors:** Qiankun Bai, Jiale Ma, Ze Zhang, Xiaojun Zhong, Zihao Pan, Yinchu Zhu, Yue Zhang, Zongfu Wu, Guangjin Liu, Huochun Yao

**Affiliations:** a MOE Joint International Research Laboratory of Animal Health and Food Safety, College of Veterinary Medicine, Nanjing Agricultural University, Nanjing, China; b Key Lab of Animal Bacteriology, Ministry of Agriculture, Nanjing, China; c Department of pathogenic diagnosis, OIE Reference Lab for Swine Streptococcosis, Nanjing, China

**Keywords:** LPxTG motif, YSIRK-G/S motif, cell wall anchoring effector, pathogenicity

## Abstract

The *Streptococcus suis* serotype 2 (SS2) is a significant zoonotic pathogen that is responsible for various swine diseases, even causing cytokine storms of Streptococcal toxic shock-like syndromes amongst human. Cell wall anchoring proteins with a C-terminal LPxTG are considered to play vital roles during SS2 infection; however, their exporting mechanism across cytoplasmic membranes has remained vague. This study found that YSIRK-G/S was involved in the exportation of LPxTG-anchoring virulence factors MRP and SspA in virulent SS2 strain ZY05719. The whole-genome analysis indicated that diverse LPxTG proteins fused with an N-terminal YSIRK-G/S motif are encoded in strain ZY05719. Two novel LPxTG proteins SspB and YzpA were verified to be exported via a putative transport system that was dependent on the YSIRK-G/S directed translocation, and portrayed vital functions during the infection of SS2 strain ZY05719. Instead of exhibiting an inactivation of C5a peptidase in SspB, another LPxTG protein with an N-terminal YSIRK-G/S motif from *Streptococcus agalactiae* was depicted to cleave the C5a component of the host complement. The consequent domain-architecture retrieval determined more than 10,000 SspB/YzpA like proteins that are extensively distributed in the Gram-positive bacteria, and most of them harbor diverse glycosyl hydrolase or peptidase domains within their middle regions, thus presenting their capability to interact with host cells. The said findings provide compelling evidence that LPxTG proteins with an N-terminal YSIRK-G/S motif are polymorphic effectors secreted by Gram-positive bacteria, which can be further proposed to define as cell wall anchoring effectors in a new subset.

## Introduction

The *Streptococcus suis* serotype 2 (SS2) is a significant zoonotic pathogen that is responsible for various swine diseases and human cases having streptococcal toxic shock syndromes [1,2]. Several factors associated with the pathogenic process of *S. suis* have been reported [3], and around one-third of them are surface proteins that function outside the cytoplasm. Various surface proteins of Gram-positive bacteria are covalently linked to the cell wall envelope by a mechanism that requires an N-terminal signal peptide and a C-terminal LPxTG motif sorting signal [4]. Several of them have been determined as vital virulence factors in pathogenic *Streptococcus* species: endo-β-N-acetylglucosaminidases that contain a C-terminal LPxTG motif are extensively present amongst *Streptococcus* species, and have the capability to cleave glycosidic bonds of host proteins during bacteria-host interactions, thus contributing to the virulence of *Streptococcus pneumoniae* [5], *Streptococcus pyogenes* [6] and *Streptococcus agalactiae* [7]; muramidase-released protein (MRP) and extracellular protein factor (Epf) of *S. suis* are prominent virulence factors, both of which contain a C-terminal LPxTG motif [8]; M-like protein SzM is a surface protein that carries an LPxTG motif, and plays key functions during *Streptococcus equi* infections [9]. Therefore, screening virulence factors from LPxTG anchoring surface proteins can contribute to further elucidate the pathogenic mechanism of SS2.

Translocation across the cytoplasmic membrane is required for the LPxTG anchoring proteins to localize to the bacterial cell surface [10], while the underlying secretion pathway remains vague. In *Staphylococcal aureus*, several LPxTG anchoring proteins have been identified to be recognized by the sortase A (SrtA), a cell wall sorting protein of the secY/A system [11,12]. Amongst these LPxTG-containing proteins, lots of them comprise a conserved YSIRK-G/S motif in their respective N-terminal regions. However, such sequence is not found in all SrtA substrates and can also be found in non-cell-wall proteins, suggesting that YSIRK-G/S is not specific to SrtA and SecY/A systems [12,13]. Combined with the predicament that the essential genes *secY/A* could not be deleted to perform a comparative secretome analysis for the identification of effectors, the said reports indicate no direct evidence to verify that these proteins export via SecY/A system currently. A previous study demonstrated that the inactivation of the YSIRK-G/S motif significantly diminished the rate of signal peptide processing and the protein secretion, whilst the cell wall anchoring or the functional assembly of protein was unaffected [13]. Consequently, it was validated that the YSIRK-G/S motif in *S. aureus* can address the proteins to cross walls, the peptidoglycan layer that forms during cell division to separate new daughter cells [14]. Indeed, some studies have indicated that YSIRK-G/S signaling-like motif portrays vital role for mediating numerous LPxTG anchoring proteins’ secretion in diverse Gram-positive bacteria [15–17], thus enabling screening more LPxTG anchoring proteins with an N-terminal YSIRK-G/S motif. This may further contribute toward exploring their potential secretion pathways and expanding our understanding to the pathogenic mechanisms of the Gram-positive bacteria.

Over the recent decades, a large number of studies have emphasized on the needlelike secretions that contain Type III, Type IV, or Type VI secretion systems (T3SS, T4SS, and T6SS) in Gram-negative bacterial species, which directly transfer related proteins across the cell envelope into the extracellular environment or the target cells [18]. These secreted proteins have been professionally defined as effectors, whilst the numerous cell surface proteins from Gram-positive bacterial species have never been identified as effectors that are exported by potential-secreted pathways. In fact, several cell wall anchoring proteins have been determined to mediate bacterial immune escapes just like many well-known T3SS effectors: the LPxTG anchoring 5ʹ-nucleotidases of *S. suis* and *S. equi* subspecies *zooepidemicus* dampen host immune responses or directly degrade neutrophil extracellular traps [19,20]; the C5a peptidases of *S. pyogenes* and *S. agalactiae* have the capability to cleave the C5a component of the host complement [21]; the EndoD like ENGases of *S. pyogenes* and *S. agalactiae* hydrolyze the host IgG [6,7]. Truly, most LPxTG anchoring proteins carry diverse functional domains within their middle regions to interact with the host to realize optimal colonization and full virulence [4]. Therefore, for *Streptococcal* pathogens including *S. suis*, the subset of LPxTG anchoring proteins that are delivered through a YSIRK-G/S directed translocation presents a kind of bacterial effector that may have been neglected for a long time. Thus, it is proposed that these proteins should be properly defined as cell wall anchoring effectors [16].

In this study, it is found YSIRK-G/S motif is required for the LPxTG-anchoring virulence factors MRP [8,22] and SspA [23] to export to the extracellular location of the SS2 virulent strain ZY05719. Following bioinformatics retrieval revealed that diverse LPxTG proteins fused with an N-terminal YSIRK-G/S motif are present in the SS2 strain ZY05719. Two novel LPxTG proteins SspB (subtilisin-like protease B) and YzpA (YSIRK-G/S-related zinc-binding protein A), were selected to validate the YSIRK-G/S dependent export and the potential roles during bacterial infection by strain ZY05719. A strategy of matching the diagnostic domain-architecture was then adopted to identify the potential effectors that delivered through a YSIRK-G/S directed translocation, employing a sample sequence with N-terminal YSIRK-G/S and C-terminal LPxTG motifs as the template. Through this approach, diverse and widely distributed potential effectors were discovered amongst the Gram-positive bacterial species. This suggests that cell wall anchoring proteins containing an N-terminal YSIRK-G/S motif are indeed polymorphic effectors secreted by pathogenic bacteria.

## Materials and methods

### Bacterial strains, plasmids, and culture conditions

The wild-type SS2 strain ZY05719 was isolated from a diseased pig during an outbreak in the Sichuan province of China. SS2 strain P1/7 and *S. agalactiae* strain A909 were maintained in the OIE Reference Laboratory for Swine Streptococcosis. All these strains were cultured at 37°C in Todd-Hewitt broth (THB, Oxoid Cheshire, UK) or THB agar (THA). The *Escherichia coli* strains were grown on Luria-Bertani (LB; BD) medium at 37°C. When necessary, antibiotics and chemicals were added at the following concentrations: spectinomycin (Sigma-Aldrich) at 100 μg/ml and sucrose at 10% (wt/vol) for *S. suis*, spectinomycin at 50 μg/ml and ampicillin (Sigma-Aldrich) at 100 μg/ml for *E. coli*. A detailed list of bacterial strains and plasmids used in this study can be found in Table S1.

### Ethics statement

Swine blood was collected to perform following studies from healthy pigs that tested negative for SS2, as determined by ELISA [24,25]. Five-week-old female-specific pathogen-free (SPF) BALB/c mice were purchased from Yangzhou University (Comparative Medicine Center). All animal experiments were performed in strict accordance with the animal welfare standards of the Animal Research Committee Guidelines of Jiangsu Province (License Number: SYXK (SU) 2017–0007), and approved by the Ethics Committee for Animal Experimentation of Nanjing Agricultural University.

### DNA manipulations and plasmids construction

DNA amplification, ligation, and electroporation were performed as previously described [26] unless otherwise indicated. All restriction and DNA-modifying enzymes were purchased from TaKaRa and performed according to supplier instruction. Deletion mutants were constructed using natural transformation method, according to two recent studies [27,28]. The forward and reverse homologous sequences of the target gene were fused with the *sacB-spc* cassette by overlap PCR. Then, the DNA products were mixed with the peptide (a key fragment of the signal factor ComX for competence regulation) and wild-type bacterial cells, incubated, and selected on THB agar (Spc^R^). The complemented strains were also constructed by the natural DNA transformation to replenish target genes containing the putative promoter sequences to the non-coding region [20] of the deleted strain genome using the *sacB-cm* cassette. Additionally, ∆*ysirk*C∆*sspB*, ∆*ysirk*C∆*yzpA* were constructed on the basis of C∆*sspB*, C∆*yzpA* with three point-mutations (ASARA) in YSIRK-G/S encoding sequence. DNA sequencing was performed by Genscript Biotechnology Co Ltd (Nanjing, China).

### Purification of recombinant protein and preparation of polyclonal antibody

Full-length gene sequences of *sspA, sspB* and *yzpA* excepting N-terminal signal peptide encoding region were amplified (primers listed in Table S2) with the genomic DNA of strain ZY05719 or P1/7. The PCR products were cloned into pET-21a vector (Table S1) following the standard molecular cloning procedures. The recombinant protein (rSspA, rSspB and rYzpA) was purified by Ni-NTA Spin Columns (QIAGEN) from BL21 (DE3) carrying the recombinant pET-21a plasmid after IPTG induction (0.1 mM) for 6 h at 37°C, and then ultrafiltered using the 30.0-kD cutoff spin columns (Millipore) to maintain homogeneity. Six five-week-old BALB/C mice in each group were immunized by purified recombinant proteins (rSspA, rSspB, and rYzpA) with 100 μg via intraperitoneal injection. The polyclonal antiserum was collected from immunized mice after 10 days of the third immunization.

### Indirect immunofluorescence analysis

An immunofluorescence microscopy assay was performed in order to visualize the localizations of SspA, SspB, and YzpA at the *S. suis* cell surface. Briefly, ZY05719, Δ*sspA*, Δ*sspB*, Δ*yzpA* and their complemented strains were grown to exponential phase, and then washed three times with sterile PBS. The 5 μl samples were adsorbed on the coverslips, and fixed in 4% paraformaldehyde-PBS. Subsequently, the bacterial cells were blocked with 5% BSA and then labeled with polyclonal antibody (anti-SspA, anti-SspB and anti-YzpA) diluted at 1:500 at 37°C for 2 h. After thrice washed, the samples were incubated with a goat anti-mouse IgG (H + L) secondary antibody, Alexa Fluor 488-conjugated (Thermo Fisher), diluted at 1:2000. Around an hour later, bacterial DNA was stained by 4′,6-diamidino-2-phenylindole (DAPI; KeyGEN BioTECH) after three times’ washing. The treated samples were finally visualized on the laser scanning confocal microscopes (Nikon Instruments, Inc. Leica Sp5 AOBS confocal system). To verify the YSIRK-G/S signal peptide is required for LPxTG proteins’ export to extracellular location, the ∆*ysirk*C∆*sspB*, ∆*ysirk*C∆*yzpA*, C∆*sspB*, C∆*yzpA* and ZY05719 were conducted the same experiment.

### Mouse infection assays

Instead of piglet infection model, BALB/c mouse model is also widely used in virulence assessment of SS2 [29–31]. Ten mice in each group were challenged with the indicated *S. suis* strain at a dose of 5 × 10^8^ CFU/mouse by intraperitoneal injection, and the clinical symptoms and death of mice were monitored every 12 h for 7 days. The negative-control group was challenged with an equal volume of sterile PBS. Additionally, a bacterial load assay was conducted to evaluate the proliferation and invasion capacity of the indicated *S. suis* strains. Mice were injected with 3 × 10^8^ CFU/mouse, and their blood and brain were harvested, weighed, and homogenized in PBS at 6 h postinfection. Subsequently, the homogenates and blood were serially 10-fold diluted and plated on THA medium to enumerate CFU.

### Cell experiments

The human laryngeal cancer epithelial cells (HEp-2) were used to perform adhesion assays, and murine macrophage-like RAW264.7 cells were used to study antiphagocytosis of SS2. These two cell lines are the classical pathogen-host interaction models for *S. suis*, and has been widly used to explore the pathogenic mechanism of SS2 [22,32,33]. The cells were cultured in Dulbecco’s modified Eagle medium (DMEM), supplemented with 10% fetal bovine serum (FBS), and maintained at 37°C with 5% CO2. Adhesion assays were performed as previously reported [34]. The bacterial cells in logarithmic growth phase (OD_600_ = 0.6) were washed twice with PBS and resuspended with DMEM culture medium to 1 × 10^7^ CFU/mL. Subsequently, the prepared bacterial cells were suspended with DMEM culture medium incubated with HEp-2 cells for 2 h at a bacterium-to-cell ratio of 100:1. The following cell lysate was serial diluted 10-fold and dropped on the THA plate and incubated at 37°C for 12 h. The adhesion rate of wild-type strain was set to 100%. All experiments were repeated three times. For the anti-phagocytosis assay, the bacterial strains in mid-exponential growth phase (OD_600_ = 0.6) were incubated with macrophages RAW264.7 for 1.5 h at a bacterium-to-cell ratio of 100:1. Then, the infected cells were washed three times with PBS and incubated in DMEM containing gentamicin (100 g/ml) and penicillin (5 g/ml) for another 1.5 h. Finally, the macrophages were washed for five times with PBS and lysed with sterile water. Serial dilutions of the cell lysate were plated onto THA plate. Each assay was repeated independently three times.

### Survival in swine serum and blood

Briefly, late log-phase bacteria were collected and washed with PBS for two times, and then were adjusted to a concentration of 1 × 10^8^ CFU/mL with sterile PBS. Subsequently, 100 µl prepared bacterial cells was added into a serum mixture, which contains 800 µl fresh swine serum from the healthy pigs and 100 µl anti-ZY05719 serum. The anti-ZY05719 serum was used to activate the complement pathway. Similarly, 100 µl prepared bacterial cells was added into a blood mixture, which contain 900ul fresh swine blood. The viable cells were determined after incubation in indicated times by plating serial dilutions onto THB plates and incubating overnight at 37°C.

### Bioinformatics

The sample sequence with the N-terminal YSIRK-G/S motif and C-terminal LPxTG motif was used as a template for bioinformatics screening using the CDART tool as previously described [35]. These analyses were performed in *Streptococcus* species at first, and then extended to all Gram-positive bacterial species. LPxTG-anchoring protein sequences of *Streptococcus* species were retrieved from the National Center for Biotechnology Information (NCBI) database, and the corresponding information are listed in Table S3, Table S4, Table S5, Figure 6 and Fig. S2. Their functional prediction was performed using HHpred (probability >50%) or Phyre2 (confidence >50%) [36,37]. BLASTP analyses were performed against the non-redundant protein database (ftp://ftp.ncbi.nih.gov/blast/db/) to identify their homologs of screened LPxTG motif-containing proteins. Phylogenetic analyses were performed following the procedures outlined by Bingle et al [38]. A ClustalW alignment was generated using the amino-acid sequences of SspA/B like C5a peptidase homologs. A phylogenetic tree was constructed using the MEGA 7.0 with the neighbor-joining method with Poisson correction and 1000 bootstrap replicates.

### Cleavage of complement C5a observed by SDS-PAGE

Recombinant protein rSspA_P1/7_, rSspB_ZY05719,_ rScpB_A909_ (100 ng) were incubated with human or mouse complement component C5a (0.5 μg) (R&D) in HEPE++ buffer (20 mM HEPES, 140 mM NaCL, 5 mM CaCL_2_, 2.5 mM MgCL_2_) in a final volume 10 μL. At the same time, SS2 strain ZY05719 and *S. agalactiae* strain A909 in stationary phase (adjusted to 4 × 10^6^ CFU) were washed twice with PBS to incubated with human or mouse C5a (0.5 μg) (R&D) with THB in a final volume 10 μl. All samples were incubated at 37°C with 5% CO_2_. After 16 h, samples were fractionated on 4–20% Bis-Tris plus gels (Genscript) and stained by Instant blue (Expedeon) for following SDS-PAGE analysis.

### RNA isolation and qRT-PCR analysis

To compare the transcription level of the *sspA, sspB* and *yzpA* genes in THB cultured cells and blood proliferating cells during infection, three five-week-old BALB/c mouse were challenged with the strain ZY05719 at a dose of 3 × 10^8^ CFU/mouse and the blood were collected at 6 h postinfection for RNA isolation. Total RNA was extracted using TRIzol reagent (Vazyme, China) according to the manufacturer’s instructions, and residual genomic DNA was then removed by digestion with DNase I (TaKaRa). cDNA was synthesized using the HiScriptII first-strand cDNA synthesis kit (Vazyme). The relative amount of target gene mRNA was normalized to the housekeeping gene *parC* transcript [39]. The relative fold change was calculated by the threshold cycle (2^−ΔΔCT^) method [40]. The reported values represented the mean ±SD of three independent RNA extractions.

### Subcellular proteins extractions

The subcellular proteins of *S. suis* strains were extracted as the previous report with minor modifications [41]. Bacterial culture of 40 ml was incubated to logarithmic phase (OD_600_ = 1.0), then centrifuged at 12,000 rpm for 10 min. For the secreted proteins preparation, the supernatant was collected and filtered through 0.22-μm-pore filters twice, then precipitated with 10% trichloracetic acid on ice for 30 min. The precipitates were washed twice with ice-cold acetone, and then the secreted proteins were acquired. For fractionated cell wall and cytoplasmic proteins, the bacterial pellets were resuspended into 2 mL buffer A which is a mixture contained 30 mM Tris-HCl, 3 mM MgCl_2_, 25% sucrose, 125 U/ml lysostaphin in pH 7.4, for a 60 min incubation at 37°C, then centrifuged at 12,000 rpm for 10 min at 4°C. The supernatant contained the cell wall proteins was precipitated with 10% trichloracetic acid on ice for 30 min, then the precipitates were washed twice with ice-cold acetone, and the cell wall proteins were acquired. The residual pellets were resuspended by the buffer which is a mixture contained 30 mM Tris-HCl and 3 mM MgCl_2_. After three freeze-thaw treatments, the samples were sonicated for a 10-s pulse and centrifuged at 12,000 rpm for 10 min, then the supernatant was collected and treated as the same steps for the secreted proteins, and the cytoplasmic proteins were obtained.

### Western blot analysis

To further reveal the subcellular localizations of bacterial proteins, a western blot analysis was performed. The proteins were separated by SDS-PAGE, and then transferred to PVDF membranes (Bio-Rad) and blocked with 5% (w/v) skimmed milk for 2 h at 37°C. Subsequently, the membranes were washed 3 times and incubated with the prepared polyclonal antibody (anti-SspA, anti-SspB, and anti-YzpA) diluted at 1:1000 by the milk at 37°C for 2 h. After thrice wash, the processed membranes were stained with the HRP conjugated secondary antibodies (Thermo Fisher, 1:2000) at 37°C for 1 h. The positive bands were detected using the 3,3ʹ-diaminobenzidine.

### Statistical analysis

Statistical analyses were performed using Prism 5.0 (GraphPad, LaJolla, CA, USA). Two-way ANOVA was performed for the qRT-PCR results, and one-way ANOVA was used for the bacterial survival assay in fresh swine serum. For infection experiments, survival data were analyzed with the log-rank test. Data from *in vivo* colonization assays were analyzed by Mann–Whitney two-tailed U tests. For all tests, a *P* value <0.05 were considered statistically significant, and all of the data were shown as mean ± SD.

## Results

### Cell wall anchoring virulence factors MRP and SspA harbored an N-terminal YSIRK-G/S motif

**Figure 1.**
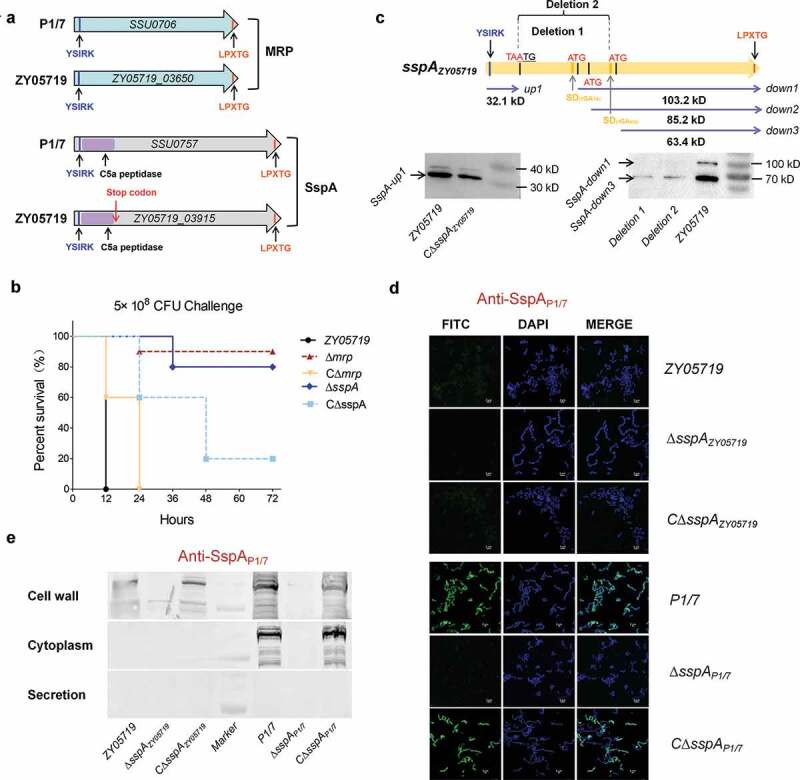
The cell wall anchoring virulence factors MRP and SspA of SS2 contain an N-terminal YSIRK-G/S signal peptide. (a) Schematic diagram of the domain organization of virulence factors MRP and SspA in SS2 strains ZY05719 and P1/7. (b) Survival curve of ZY05719, ∆*sspA*, ∆*mrp* and their complemented strains in the mouse infection model. The five-week-old BALB/c mice were infected with indicated SS2 strains at the same dose and monitored over a 7-day period. The figure showed the data for 72 h postinfection. After 72 h, there were no newly dead mice. (c) Schematic diagram of the pseudogene *sspA* in strain ZY05719, and identification of its protein production by Western Blot using the anti-SspA_P1/7_ serum. A frameshift mutation at the position of 967 bp generates a stop codon to divide *sspA* into two segments *sspA*-up carrying YSIRK-G/S motif and *sspA*-down containing LPXTG domain. Multiple putative ORFs were predicted in *sspA*-down fragment. Deletion 1: the fragment from 945 to 2201 bp was deleted to damage the first ORF; Deletion 2: the fragment from 945 to 2873 bp was deleted to damage the first and second ORFs. The protein samples for western blot were prepared using whole-cell lysate. (d) To verify whether SspA-up and SspA-down could be secreted and anchored to the cell wall, the indirect immunofluorescence tests were performed using an anti-SspA_P1/7_ serum. The surface proteins, but not the intracellular proteins, could be recognized by the extracellular SspA_P1/7_ antiserum. CΔ*sspA* is the complemented strain of ΔsspA mutant. (e) Western blot analysis identified the subcellular localizations of protein SspA in strains P1/7 and ZY05719

Several cell wall surface proteins of gram-positive bacteria are covalently anchored to the cell wall by a mechanism that requires a C-terminal anchoring motif LPxTG [42,43], and are typically identified as required for bacterial pathogenicity. In *S. suis*, muramidase-released protein (MRP) and subtilisin-like serine protease (SspA), two prominent virulence factors, were found to encode a C-terminal LPxTG motif (Figure 1a), and thus anchored to the cell wall to play key functions within the pathogen-host interaction [22,23]. This study determined that the deletion of *mrp* or *sspA* caused a significantly higher survival rate compared to a wild-type strain (100% death rate) in a mouse infection model (Figure 1b), thus suggesting that the two aforementioned proteins were likewise involved in the bacterial pathogenicity of the clinical SS2 isolate ZY05719. However, a frameshift mutation had occurred in the *sspA* gene of strain ZY05719 to generate a termination codon at the 946 bp position, which split the gene into two segments: redesignated as *sspA*-up and *sspA*-down. Further study found three potential initiation codons existing within the *sspA*-down to generate three new ORFs. Thus, two deletion mutants were constructed as shown in Figure 1c to validate the production of SspA-down. A Western blot analysis confirmed the presence of one SspA-up at 32.1kD, and two SspA-downs at 103.2 kD and 63.4 kD (Figure 1c). The samples from strains Deletion 1 (deleted 945–2201 bp to damage the first ORF) and Deletion 2 (deleted 945–2873 bp to damage the first and second ORFs) depicted the protein band only at 63.4 kD, implying that the predicted ORF SspA-down2 did not actually produce protein.

Afterward, it was probed whether SspA-up or SspA-down could be secreted and anchored to the cell wall. As illustrated in Figure 1d, the indirect immunofluorescence assays applying an anti-SspA_intact_ serum depicted that neither SspA-up nor SspA-down carrying a C-terminal LPxTG motif could be anchored to the bacterial surface, whilst the intact SspA presented an apparent surface localization on the P1/7 cells. Then, a Western blot analysis using an anti-SspA_P1/7_ serum was performed to identify the subcellular localizations of the protein SspA on strains P1/7 and ZY05719. As illustrated in Figure 1e, the interrupted protein SspA in strain ZY05719 could not be detected both in the cell wall and in the culture supernatant; instead, it was observed in the strain P1/7 samples. These observations have indicated that the breakaway fragment of SspA-up is required for the subcellular localization of SspA on the cell surface. A sequence comparison of homologs from strains P1/7 and ZY05719 found that both SspA and MRP encoded an N-terminal YSIRK-G/S motif. A succeeding test using the SignalP server 5.0 software predicted YSIRK-G/S as a vital signal peptide to mediate the transport of extracellular proteins. Further analysis of the several virulence factors with a C-terminal LPxTG motif from the other *Streptococcus* species identified that most of them encode an N-terminal YSIRK-G/S motif, such as the M-like protein SzM of the *S. equi* subspecies *zooepidemicus* [9], the C5a peptidase ScpB of *S. agalactiae* [21] and the zinc metalloprotease ZmpB of *S. pneumoniae* [44]. These observations have suggested that the YSIRK-G/S-directed translocation may portray functions during the infection of *Streptococcus* pathogens, which have prompted screening more effectors secreted by this approach for a better clarification of bacterial pathogenesis.

### Diverse LPxTG proteins fused with an N-terminal YSIRK-G/S signal peptide were encoded in SS2 strain ZY05719

Bioinformatics retrieval of the marker domain has been successfully applied to the effectors’ screening of bacterial secretion systems [45,46], hence, using a YSIRK-G/S motif as a marker to screen the potential effectors delivered through a YSIRK-G/S-directed translocation can be an alternative proposal. Since this study primarily focuses on exploring the pathogenic roles of effectors’ translocation that is directed by YSIRK-G/S during SS2 infection, mainly screening virulence-related effectors from cell wall anchoring proteins can be an effective method. As presented in Figure 1d, neither SspA-up nor SspA-down proteins could be anchored to the bacterial surface, implying that both YSIRK-G/S and LPXTG motifs might be required for an accurate subcellular localization and biological function of such proteins. Therefore, 23 predicted LPxTG proteins from the genomes of SS2 strains P1/7 and ZY05719 were screened to analyze the encoding of YSIRK-G/S motifs in their respective N-terminal regions (Table 1). A total of 10 proteins including six prominent virulence factors were identified to include an N-terminal YSIRK-G/S motif, thus denoting as the potential cell wall anchoring effectors secreted by the putative transport system. Consequently, two unknown proteins *zy05719_07925* and *zy05719_09405* were selected for further examination in the virulent SS2 strain ZY05719. Notably, the expression of the said genes has been proven to be significantly up-regulated during infection using an *in vivo* induced antigen technology (IVIAT) [47].

**Table 1.** LPxTG proteins with or without N-terminal signal peptide YSIRK-G/S in *Streptococcus suis* serotype 2. The genomic accession No. of SS2 strain P1/7 and ZY05719 are AM946016 and CP007497, respectively

P1/7	ZY05719	Description	YSIRK-G/S	LPxTG-like	Reference
*SSU0171/72^a^*	*ZY05719_00985/90/95^a^*	Extracellular protein factor, Epf	Y	Y	[8]
*SSU0186*	*ZY05719_01065*	Unknown	Y	Y	[56]
*SSU0201*	*ZY05719_01130*	Unknown	N	Y	-
*SSU0253*	*ZY05719_01380*	Atrophin	N	Y	-
*SSU0255 ^a^*	*ZY05719_01390*	Transposase	N	Y	-
*SSU0427*	*ZY05719_02340*	Unknow	N	Y	-
*SSU0496*	*ZY05719_02665*	Full = IgM protease	Y	Y^1^	[57]
*SSU0587*	*ZY05719_03065*	Glycosyl hydrolase family protein	N	Y	-
*SSU0706*	*ZY05719_03650*	Muramidase-released protein precursor, MRP	Y	Y	[8,22]
*SSU0757*	*ZY05719_03915 ^a^*	C5a_Peptidase	Y	Y	[23]
*SSU0860*	*ZY05719_04860*	MPP	N	Y	-
*SSU0879*	*ZY05719_04950*	IgA-specific zinc metalloproteinase	Y	Y	[58]
*SSU1128*	*ZY05719_06180*	Unknow	N	Y^2^	-
*SSU1143*	*ZY05719_06260*	M14_Peptidase	N	Y	[59]
*SSU1201*	*ZY05719_06545*	Unknow	N	Y	-
*SSU1215*	*ZY05719_06615*	Peptidase C69	N	Y	[60]
*SSU1355*	*ZY05719_07310*	MPP	N	Y	-
*SSU1476*	*ZY05719_07925*	Unknown, LPXTG protein	Y	Y	[47]
*SSU1715*	*ZY05719_09105*	Endo-beta-N-acetylglucosaminidase	Y	Y	-
*SSU1773*	*ZY05719_09405*	C5a_Peptidase	Y	Y	[47]
*SSU1849*	*ZY05719_09785*	Surface-anchored amylopullulanase	Y	Y	[61]
*SSU1879*	*ZY05719_09925*	2ʹ,3ʹ-cyclic-nucleotide 2ʹ-phosphodiesterase	N	Y	[62,63]
*SSU1888*	*ZY05719_09960*	Pilus subunit protein	N	Y^3^	[64]

^a^: pseudogene; ^1^: LGSTG; ^2^: LPXTS; ^3^: YPKTG.

### YSIRK-G/S motif was required for LPxTG proteins’ export to extracellular locations

**Figure 2.**
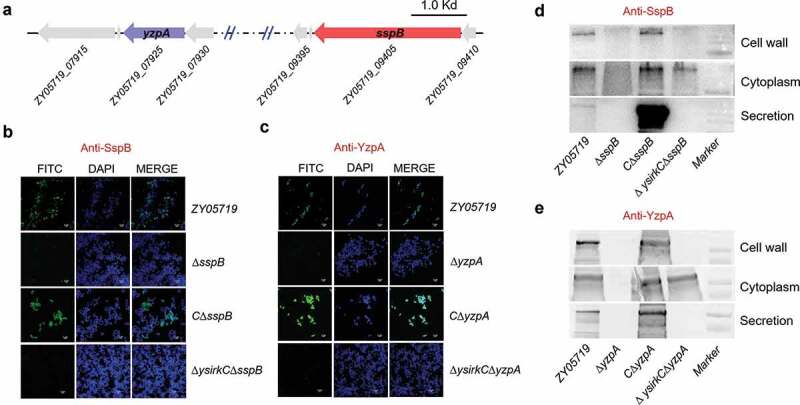
YSIRK-G/S motif is required for the export of SspB and YzpA to extracellular location. (a) Schematic diagram of the genetic neighborhood of genes *sspA* and *yzpA* in *S. suis* strain ZY05719. Immunofluorescence test identified SspB (b) and YzpA (c) to be secreted and anchored to the cell wall using the anti-SspB and anti-YzpA serum, respectively. It should be noted that the intracellular proteins could not be recognized by the extracellular antiserums. (d, e) Western blot analyses identified the subcellular localizations of proteins SspB and YzpA in the indicated strains

The genetic neighborhoods of genes *zy05719_09405* and *zy05719_07925* were validated (Figure 2a); their encoding proteins were redesignated as SspB (subtilisin-like protease B) and YzpA (YSIRK-G/S related zinc-binding protein A), respectively. To verify whether these two proteins were secreted to extracellular locations and anchored to the cell wall, an indirect immunofluorescence assay was conducted to detect their subcellular localizations using anti-SspB and anti-YzpA antibodies, which were prepared by immunizations on mice using recombinant proteins rSspB and rYzpA, respectively. As illustrated in Figure 2b,c, the green fluorescence of proteins SspB and YzpA was detected on 70% to 80% of the surface of cells in wild-type ZY05719 strain, but not on the surface of Δ*sspB* or Δ*yzpA* cells using the same detection approach. The expression of *sspB* and *yzpA* was controlled at a low level in the wild-type strain, which might cause a missed observation of the fluorescence completely suffusing the cell surface. However, when the genes *sspB* and *yzpA* were, respectively, reintroduced into the Δ*sspB* and Δ*yzpA* cells, the specific green fluorescence was brighter than that of wild-type cells (Figure 2b,c), further indicating that the two proteins were successfully secreted and anchored to the cell surface. It was then tested whether the YSIRK-G/S motif was required for the proteins’ exportation, hence, such motif was destroyed by point mutations to construct the mutants Δ*ysirk*CΔ*sspB* and Δ*ysirk*CΔ*yzpA*. As presented in the bottommost panels of Figure 2b,c, indirect immunofluorescence detected specific green fluorescence on the cell surfaces of strains CΔ*sspB* and CΔ*yzpA*, but not on point mutation strains Δ*ysirk*CΔ*sspB* and Δ*ysirk*CΔ*yzpA*. Western blot analyses determined that SspB could not be detected in the cell wall and culture supernatant of the strain Δ*ysirk*CΔ*sspB*, but was rather correctly produced and intracellularly accumulated prior to exportation (Figure 2d). Similar results were likewise found in strain Δ*ysirk*CΔ*yzpA* (Figure 2e). These findings implied that LPxTG-anchoring proteins SspB and YzpA were indeed exported through a YSIRK-G/S-dependent approach, and were thus identified as the cell wall anchoring effectors of SS2.

### Cell wall anchoring effector SspB was required for full-virulence of SS2 strain ZY05719

**Figure 3.**
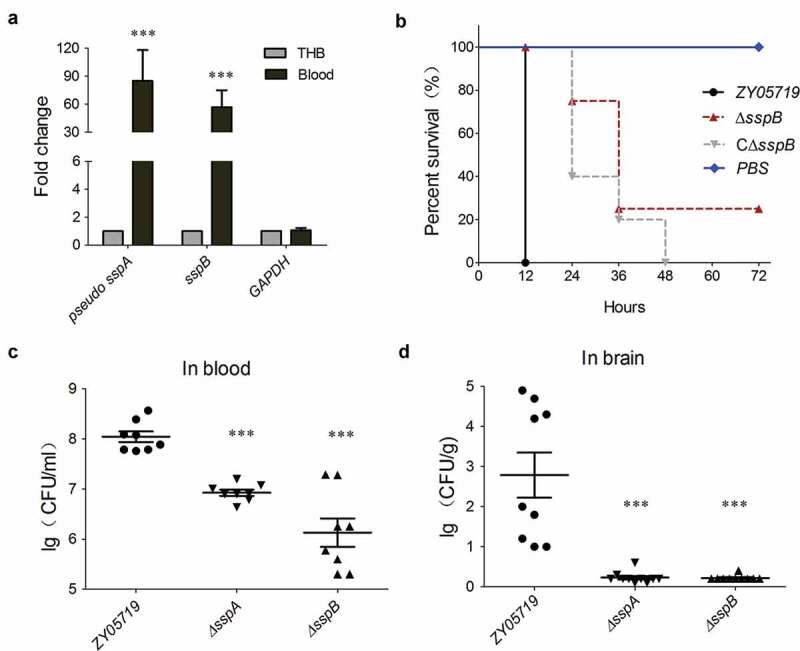
SspB is critical for full-virulence of SS2 strain ZY05719. (a) Identification of the transcriptional activation of *sspB* during SS2 infection comparing with the THB culture. The data were normalized to the housekeeping gene *parC* transcript [39]. The relative expression levels represented the mean ±SD of three biological repeats (*** *P* < 0.01). (b) Survival curve of ZY05719, Δ*sspB* and CΔ*sspB* strains in the mouse infection model. The five-week-old BALB/c mice were infected with indicated SS strains at the same dose and monitored over a 7-day period. Mice inoculated with PBS were served as a negative control. The figure showed the data for 72 h postinfection. After 72 h, there were no newly dead mice. (c, d) Systemic infection experiments were conducted to assess the bacterial proliferation in mouse blood and brain. Bacterial reisolation from the blood or brain at 6 h postinoculation was quantified by plate count. Statistical significance was determined by a Student’s *t* test based on comparisons with the wild-type group (*** *P* < 0.001)

Through bioinformatics analysis, both SspA (a prominent virulence factor [23]) and SspB were predicted to encode an N-terminal YSIRK-G/S motif, a C5a peptidase domain and a C-terminal LPxTG motif. The qRT-PCR results depicted that both *sspA* and *sspB* of strain ZY0519 were significantly upregulated during the bloodstream infection of a BALB/c mouse model (Figure 3a), which partially verified the IVIAT screening study [47], and suggested that SspB might be involved in SS2 pathogenicity. Subsequently, a virulence assessment test was conducted using BALB/c mice injected with 5 × 10^8^ CFU of related mutant and wild-type strains. As illustrated in Figure 3b, the mice that were challenged with wild-type and complemented strains developed serious clinical symptoms within 12 h; all of them died within 48 h. In contrast, the mice that were challenged with ∆*sspB* presented slight clinical signs up to 36 h postinfection, and eventually obtained a 25% survival rate (Figure 3b). The growth curves manifested no significant differences amongst the ZY05719, ∆*sspB* and C∆*sspB* strains (Fig. S1). The role of SspB in SS2 fitness in the host bloodstream and brain was further verified. Figure 3c,d depicts that the deletion of *sspB* or *sspA* significantly attenuated the bacterial loads in mice brain and blood compared to the wild-type strain. Overall, the findings have indicated that the cell wall anchoring protein SspB contributes to the full virulence of SS2 in the host bloodstream and brain during systemic infection.

### SspB was inactivated in C5a peptidase activity to attack the host complement for the escape of serum killing

**Figure 4.**
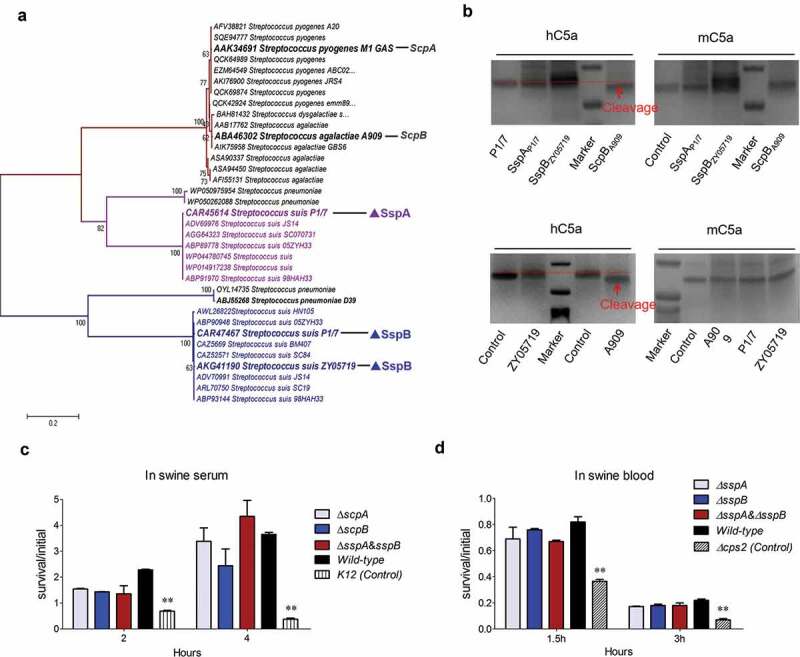
SspB is inactivated in C5a peptidase activity to attack host complement. (a) Phylogenetic analysis of C5a peptidases from *Streptococcus* species. A neighbor-joining tree (bootstrap n = 1000; Poisson correction) was constructed based on a ClustalW alignment of the amino acid sequences of SspB and its homologs from diverse *Streptococcus* species using the MEGA software version 5.0. The representative C5a peptidase homologs were labeled by overstriking and increasing font size. (b) Cleavage of human and mouse complement factors C5a (0.5 μg) by whole-cell pellets (4 × 10^6^ CFU) and recombinant protein rSspA and rSspB (100 ng). Tests were performed at 37°C for 16 hours and visualized by SDS-PAGE and Coomassie staining. (c, d) Survival rate of indicted *S. suis* strains in fresh swine serum and blood. The bacterial cells (1 × 10^7^ CFU) were added into the fresh swine serum or blood, and incubated in 5% CO_2,_ 37°C for indicated times. Data were shown as the mean ± SEM of three independent experiments performed in triplicate. The laboratory K12 strain of *E. coli* and ∆ *cps2* mutant of ZY05719 were used as the positive controls

Several existing studies have reported that the homologs of LPxTG-anchoring C5a peptidase from *S. pyogenes* and *S. dysgalactiae* can cleave the human complement component C5a to release abnormally sized and functionally impaired fragments, thus facilitating the bacterial cells to escape phagocytosis and clearance from the host’s innate immunity [21]. Both SspA and SspB harbored C5a peptidase domain; hence, it should be verified whether to perform a similar function in *S. suis*. A phylogenetic analysis of C5a peptidase homologs from *Streptococcus* species depicted that all proteins were divided into three distinct clades (Figure 4a). Notably, SspA and SspB were located at distinct branches, and exhibited a great evolutionary distance from the first branch that contained prominent C5a peptidase homologs from the *S. pyogenes* strain M1 and the *S. dysgalactiae* strain A909 (Figure 4a), thus applying a potential functional difference between them. To verify the function of both SspA and SspB in C5a peptidase, the hydrolytic activities of the recombinant proteins SspA_P1/7_ and SspB_ZY05719_ on human and mouse C5a were evaluated. As illustrated in the upper panel of Figure 4b, both hC5a and mC5a that were co-incubated with SspA_P1/7_ and SspB_ZY05719_ presented no size reduction compared to C5a alone when visualized by SDS-PAGE gels, whilst the ScpB_A909_ treated hC5a manifested a significant size reduction. These findings have implied that the human or mouse C5a is not a substrate of cell wall anchoring peptidases SspA and SspB of *S. suis*. Further test evaluated the cleavage activity of whole bacterial cells in SS2 strain ZY05719, P1/7 and *S. agalactiae* strain A909 (4 × 10^6^ CFU) to hC5a and mC5a. Similar to both SspA_P1/7_ and SspB_ZY05719_, the SS2 cells of strains ZY05719 and P1/7 could not cleave the complement component from either the human or mouse C5a (Figure 4b), while the *S. agalactiae* cells of strain A909 depicted a significant cleavage to the human C5a. The cleaved protein band by ScpB_A909_ or strain A909 was identified by mass spectrometry, and illustrated that the C-terminal 7aa fragment of human C5a was cut off. This result is consistent with a previous study [48], and validates that both SspA and SspB of *S. suis* may have no C5a peptidase activity to attack the host complement for escape of serum killing. Indeed, the ∆*sspA* and ∆*sspB* deletion mutant strains have presented no significant difference in swine serum and blood survival compared to the wild-type strain (Figure 4c,d). Indeed, the underlying pathogenic mechanisms of SspA and SspB during SS2 infection need to be further explored.

### Cell wall anchoring effector YzpA was required for full-virulence of SS2 strain ZY05719 by anti-phagocytosis

**Figure 5.**
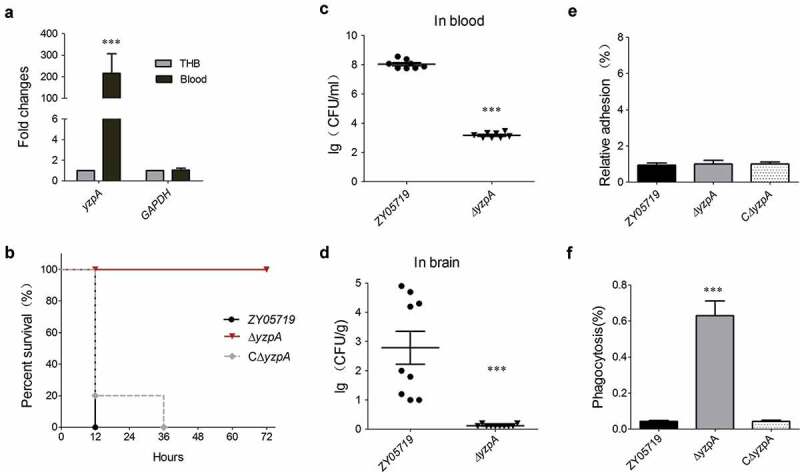
YzpA is required for full-virulence of SS2 strain ZY05719 by anti-phagocytosis. (a) Identification of the transcriptional activation of *yzpA* during SS2 infection comparing with the THB culture. The data were normalized to the housekeeping gene *parC* transcript [39]. The relative expression levels represented the mean ±SD of three biological repeats (*** *P* < 0.01). (b) Survival curve of ZY05719, Δ*yzpA* and CΔ*yzpA* strains in the mouse infection model. The five-week-old BALB/c mice were infected with indicated SS strains at the same dose and monitored over a 7-day period. Mice inoculated with PBS were served as a negative control. (c, d) Systemic infection experiments were conducted to assess the bacterial proliferation in mouse blood and brain. Bacterial reisolation from the blood or brain at 6 h postinoculation was quantified by plate count. Statistical significance was determined by a Student’s *t* test based on comparisons with the wild-type group (*** *P* < 0.001). (e) The role of YzpA on the ability of SS2 to adhere to HEp-2 cells. There was no significant difference in adhesion rate observed between ZY05719, ∆*yzpA* and C∆*yzpA* after 2 h co-incubation. (f) The role of YzpA on the ability of SS2 to resist phagocytosis to RAW 264.7 after 1.5 h co-incubation. Data were shown as the mean ± SEM of three independent experiments performed in triplicate. Two-tailed unpaired Student’s tests were used for statistical analysis (***, *P* < 0.001)

Another screened cell wall anchoring effector that was delivered through YSIRK-G/S-directed translocation was the *zy05719_07925* encoding protein YzpA (Table 1). The qRT-PCR results presented that the *yzpA* of strain ZY0519 was significantly upregulated during bloodstream infection of the BALB/c mouse model (Figure 5a), implying that it might be involved in SS2 pathogenicity. Indeed, the mice challenged with Δ*yzpA* indicated a significantly higher survival rate (100%), with or without slight clinical signs, compared with the 100% death of mice infected by the wild-type strain, which manifested acute clinical signs, including shivering, rough hair coat, and depression (Figure 5b). The role of YzpA in SS2 fitness during systemic infection was then verified. As presented in Figure 5c,d, the deletion of *yzpA* significantly attenuated the bacterial loads in mice brain and blood compared to the wild-type strain. Altogether, the data indicated that the cell wall anchoring effector YzpA delivered by the YSIRK-G/S directed translocation contributed to the full virulence of SS2.

Adhesion is the first step for bacterial cells to invade host tissues [49]. Unexpectedly, the adhesion of Δ*yzpA* had exhibited no significant difference compared to the wild-type strain, insinuating that the cell surface location of YzpA was not involved in bacterial adhesion (Figure 5e). Meanwhile, anti-phagocytosis against macrophages is considered as another critical step for the bacterial pathogens to cause systemic infection [50]. As indicated in Figure 5f, the *yzpA* deletion mutant strain was more readily phagocytosed by the RAW264.7 cells, thus depicting a significantly attenuated anti-phagocytosis versus the wild-type strain. These results have implied that YzpA is involved in anti-phagocytosis to play key functions in the full virulence of SS2.

### *Cell wall anchoring effectors delivered through YSIRK-G/S-directed translocation are widespread in* Streptococcus *species*

The cell wall anchoring proteins with a C-terminal LPxTG motif are distributed extensively in Gram-positive bacteria [42], and have been confirmed in this study to fuse with an N-terminal YSIRK-G/S motif for secretion in *S. suis*. It was speculated that a few of these LPxTG-anchoring proteins might carry diverse function domains in their middle regions to serve as effectors that were delivered through YSIRK-G/S-directed translocation in numerous *Streptococcus* species. Thus, a whole-genome search of the LPxTG-anchoring proteins was then performed in strains *S. suis* serotype 9 (SS9) GZ0565, *S. pneumoniae* D39, *S. agalactiae* A909 and *S. equi* ATCC 35,246, and found more than 40 target proteins (Table 2). To further validate the N-terminal sequences, a total of 26 (more than 60%) proteins encoded a YSIRK-G/S motif (Table 2), and were predicted as the potential effectors delivered through YSIRK-G/S-directed translocation. Thereafter, at least 15 proteins including 5ʹ-nucleotidase [20], C5a peptidases [21], Fibronectin-binding proteins [51], and zinc metalloprotease ZmpB [44], were identified as vital virulence factors [52], further implying that the YSIRK-G/S-directed translocation had contributed to export diverse cell wall anchoring effectors to be involved in the pathogenicity of the *Streptococcus* species. Applying protein homology detection programs, HHpred and Phyre2 [36,37], it was found that the potential effectors encoded protease, host component binding, glycosyl hydrolase, and nuclease domains, with the exception of a few domains with unknown function (Table 2). Such an extensive distribution of cell wall anchoring proteins highlights their ability to acquire potential functions, thus acting as new effectors exported through YSIRK-G/S-directed translocation.

**Table 2.** LPxTG proteins with or without N-terminal signal peptide YSIRK-G/S in *S. suis* serotype 9 (SS9), *S. pneumoniae, S. agalactiae, S. equi* subsp zooepidemicus. The genomic accession No. of SS9 GZ0565, *S. pneumoniae* D39, *S. agalactiae* A909 and *S. equi* subspecies *zooepidemicus* are CP017142, CP00410, CP000114 and CP002904 respectively

Gene locus	Strain	Description	YSIRK-G/S	LPxTG-like	Reference
BFP66_00950	SS9 GZ0565	chimeric erythrocyte binding protein	Y	Y	-
BFP66_01270	SS9 GZ0565	cell surface protein	N	Y	-
BFP66_01275	SS9 GZ0565	methyl-accepting chemotaxis protein	N	Y	-
BFP66_03605	SS9 GZ0565	hyaluronidase	Y	Y	-
BFP66_04530	SS9 GZ0565	glucan-binding protein	N	Y	-
BFP66_04855	SS9 GZ0565	bifunctional metallophosphatase/5ʹ-nucleotidase	N	Y	[^20]^
BFP66_05510	SS9 GZ0565	Glucan-binding protein C	Y	Y	-
BFP66_05275	SS9 GZ0565	serine protease	Y	Y	-
BFP66_06160	SS9 GZ0565	Glycosyl hydrolase family 20 (GH20) catalytic	N	Y	-
BFP66_06465	SS9 GZ0565	Mac family protein	Y	Y	[^65]^
BFP66_06960	SS9 GZ0565	bifunctional metallophosphatase/5ʹ-nucleotidase	N	Y	[^20]^
BFP66_07660	SS9 GZ0565	Unknown	Y	Y	-
BFP66_08445	SS9 GZ0565	G5 superfamily protein	Y	Y	-
BFP66_09230	SS9 GZ0565	Nuclease	N	Y	[^20]^
SPD_0063	*S. pneumoniae* D39	beta-N-acetylhexosaminidase	Y	Y	[^66,67]^
SPD_0080	*S. pneumoniae* D39	Fibronectin-binding	Y	Y	[^51]^
SPD_0250	*S. pneumoniae* D39	pullulanase, extracellular	Y	Y	-
SPD_0335	*S. pneumoniae* D39	endo-alpha-N-acetylgalactosaminidase	Y	Y	-
SPD_0444	*S. pneumoniae* D39	endo-beta-N-acetylglucosaminidase D	N	Y	[^5]^
SPD_0562	*S. pneumoniae* D39	beta-galactosidase precursor	Y	Y	-
SPD_0577	*S. pneumoniae* D39	zinc metalloprotease ZmpB	Y	Y	[^44]^
SPD_1018	*S. pneumoniae* D39	Immunoglobulin A1 protease precursor	Y	Y	[^53]^
SPD_1376	*S. pneumoniae D39*	chimeric erythrocyte binding protein	Y	Y	-
SPD_1617	*S. pneumoniae D39*	cell wall surface anchor family protein	Y	Y	-
SPD_2017	*S. pneumoniae* D39	choline binding protein A	Y	Y	[^68]^
SPD_1321	*S. pneumoniae* D39	cell wall surface anchor family protein	N	Y	-
SPD_1789	*S. pneumoniae* D39	cell wall surface anchor family protein	N	Y	-
SAK_0186	*S. agalactiae* A909	IgA-binding beta antigen	Y	Y	[^69]^
SAK_1991	*S. agalactiae* A909	cell surface serine endopeptidase CspA	Y	Y	-
SAK_1320	*S. agalactiae* A909	C5a peptidase	Y	Y	[^21]^
SeseC_00398	*S. equi* ATCC 35246	cell surface-anchored protein	N	Y	-
SeseC_00614	*S. equi* ATCC 35246	serine endopeptidase ScpC, lactocepin	Y	Y	[^70]^
SeseC_00619	*S. equi* ATCC 35246	MUCin-Binding Protein	Y	Y	-
SeseC_00994	*S. equi* ATCC 35246	collagen-like protein	N	Y	-
SeseC_00180	*S. equi* ATCC 35246	fibrinogen- and Ig-binding protein precursor	N	Y	-
SeseC_01072	*S. equi* ATCC 35246	UDP-N-acetylgl 1-carboxyvinyltransferase	N	Y	[^71]^
SeseC_01085	*S. equi* ATCC 35246	Szp protein	Y	Y	[^72]^
SeseC_01092	*S. equi* ATCC 35246	cell surface-anchored protein	N	Y	-
SeseC_02415	*S. equi* ATCC 35246	IgG Fc-binding protein SzM	Y	Y	[^9]^
SeseC_02304	*S. equi* ATCC 35246	sspB-C2 type	Y	Y	-
SeseC_01703	*S. equi* ATCC 35246	hydrolase HAD family	Y	Y	-

**Figure 6.**
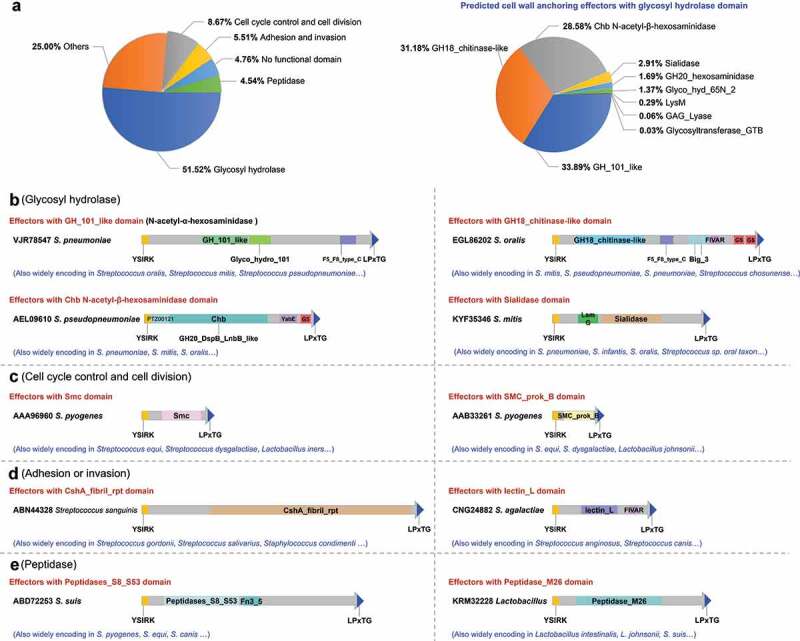
Potential cell wall anchoring effectors delivered through the YSIRK-G/S directed translocation are found across various Gram-positive bacteria species. (a) Distribution of predicted functional domains divided into six groups (left panel), and distribution of nine main glycosyl hydrolase domains (right panel). The domain-architectures of predicted cell wall anchoring effectors from the glycosyl hydrolase encoding group (b), cell cycle control domain encoding group (c), adhesion or invasion domain encoding group (d) and peptidase encoding group (e), are presented briefly with different colored boxes

The identification of cell wall anchoring effectors according to their specific domain architecture, namely, the N-terminal YSIRK-G/S motif and the C-terminal LPxTG domain, has been demonstrated to be successful in *S. suis* both bioinformatically and experimentally. The retrieval of specific domain architecture was then extended to more pathogenic bacteria species. After exception of the retrieved proteins with YSIRK-G/S or LPxTG motif out of the C- or N- terminus (Table S4) by further classification, more than 10,000 potential cell wall anchoring effectors were identified in more than 60 bacterial species (Table S3). These potential effectors had exhibited considerable diversity with different permutations of domains in their middle regions, which could be classified into 394 types (Table S3). Based on functional prediction, the effectors could be divided into six groups: 1) glycosyl hydrolase; 2) cell cycle control and cell division; 3) adhesion and invasion; 4) peptidase; 5) have no functional domain; and, 6) others (Figure 6a). The domain architectures of the representative proteins were presented in Figure 6b–e. Notably, more than 65% of the retrieved effectors were encoded by *Streptococcus* species, and 51.52% harbored glycosyl hydrolase domains in their middle regions (Figure 6a). Further analysis classified the glycosyl hydrolase domains into nine types: GH_101_like, GH18_chitinase-like, Chb N-acetyl-β-hexosaminidase, Sialidase, GH20_hexosaminidase, Glyco_hyd_65N_2, LysM, GAG_Lyase and Glycosyltransferase_GTB (right panel of Figure 6a, Fig. S2). These observations indicate that cell wall anchoring effectors typically exist in Gram-positive bacteria.

## Discussion

Secretion systems have been confirmed to play key functions in pathogen–host interaction, thus contributing to the full virulence of numerous bacteria [18]. However, most of them are encoded only by Gram-negative bacteria [18], and are neglected for conducing related studies in Gram-positive bacteria. This study has confirmed that the diverse LPxTG proteins fused with an N-terminal YSIRK-G/S motif are export to extracellular location in numerous *Streptococcus* species, and has s proposed to identify this new subset of secreted proteins as cell wall anchoring effectors delivered through YSIRK-G/S-directed translocation.

For the putative effector SspA, it should be noted that the ORF of gene *sspA* is interrupted by frameshift mutation in strain ZY05719, whilst it is still involved in the virulence of such strain. The Western blot analysis presented that the interrupted *sspA* could produce three fragments of SspA; as such, future works should be done to explore their potential roles during bacterial infection. Meanwhile, two novel LPxTG-anchoring effectors SspB and YzpA were selected to explore their potential roles during bacteria-host interaction and the pathogenic processes in an animal infection model. Despite significant transcriptional upregulation of *sspB* and *yzpA* in infected blood, and significant decrease of bacterial loads and mortality in Δ*sspB* and Δ*yzpA* mutant strains was observed in the animal infection model, the biological functions of SspB and YzpA were not fully elucidated in this study. SspB contains a C5a peptidase domain, but succeeding tests have revealed that both human and mouse C5a cannot be cleaved by co-incubation with the recombinant SspB. Arguably, SspB may specifically cleave the C5a component of swine complement system, but not the C5a homologs from other hosts. To explore the potential roles of SspB against the bactericidal activity of the swine complement system, the growth characteristics of wild-type ZY05719, Δ*sspA* and Δ*sspB* strains in fresh swine blood and serum with anti-ZY05719 antibodies were compared. No significant difference was found in the survival rate amongst these strains, thus suggesting that the predicted attack from SspB to the swine complement system was ineffective or inexistent. Certainly, the cleaving activity of a professional peptidase is always released under strict incubation conditions, including specific temperature, pH, metal ions, special substrate modifications, and some other vague terms. It is not easy to meet an unknown reaction condition or fully simulate the internal environment of the host cells or tissues *in vivo*. For another protein, this study has presented that YzpA facilitates bacterial cells to avoid host clearance through anti-phagocytosis, therefore playing a crucial role during *S. suis* infection. However, regrettably, little information can be found by bioinformatics approach to determine a clear direction for further clarification of the biological function of YzpA.

One windfall of the C5a peptidase activity tests was that the positive control protein from the *S. agalactiae* strain A909, ScpB [21], was found to encode a C-terminal LPxTG motif, thus being identified as one of the cell wall anchoring effectors with a clarified biological function. This result indirectly indicates that the proteins’ translocation directed by YSIRK-G/S indeed contributes to bacteria-host interaction and evasion from host immune clearance by exporting specific cell wall anchoring effectors. Further analysis has determined that the biological functions of numerous cell wall anchoring effectors have been clarified, such as the IgA1 protease of *S. suis, S. pneumoniae* and *Neisseria meningitidis* for IgA degradation [53], M-like protein SzM of *S. equi* for antibody binding [9], and IgG endopeptidase Mac of *S. pyogenes* and *S. equi* for IgG degradation [54,55]. These observations imply that cell wall anchoring effectors delivered through YSIRK-G/S-directed translocation play vital roles in the pathogenic processes of Gram-positive bacteria.

This study primarily focused on the retrieval and identification of virulence-related effectors that were delivered through YSIRK-G/S-directed translocation from cell wall anchoring proteins to better illustrate the pathogenic mechanisms of *Streptococcus* species; thus, numerous secretory proteins that only harbored only an N-terminal YSIRK-G/S motif were filtered out from the screening results. In fact, this type of secretory proteins is widely distributed in Gram-positive bacteria (Table S5), and most of them are delivered to the extracellular space to participate in the physiological processes of the bacteria themselves. However, there are still many other proteins that carry potential effect domains (Table S5), including diverse glycosyl hydrolases (e.g., sialidase, LysM) and peptidases (e.g., M26, abhydrolase), which are predicted to sense environmental shifts in extracellular spaces and mediate bacteria-host interactions. In this case, further study should be conducted to clarify the functional mechanisms of this type of effectors.

Amongst the retrieved cell wall anchoring effectors (Table S3), the glycosyl hydrolase and peptidase encoding proteins should be more deeply explored. The several proteins from these two types have been confirmed to play significant functions in both bacteria-host interaction and pathogenic processes, such as Endo-β-N-acetylglucosaminidases [5–7], C5a peptidases [21], IgA1 protease [53]. In particular, the three encoded domains of potential effectors, including GH_101_like N-acetyl-α-hexosaminidase, Chb N-acetyl-β-hexosaminidase and GH20_hexosaminidase, were predicted to portray similar enzymic activities to the Endo-β-N-acetylglucosaminidase homologs, thus suggesting their potential roles during bacterial infection. Further data analysis from domain architecture retrieval determined that the encoded YSIRK-G/S or LPxTG motif is not located at the N- or C-terminus of around 8.6% of the proteins (Table S4), including: 1) N-terminal YSIRK-G/S and non-C-terminal LPxTG; 2) non-N-terminal YSIRK-G/S and C-terminal LPxTG; and 3) non-N-terminal YSIRK-G/S and non-C-terminal LPxTG. The first protein group was predicted to be delivered through YSIRK-G/S-directed translocation to the extracellular space, but the localization on the cell surface remained indeterminate. The second group needed to be further explored as to whether they can be exported to the extracellular space. Meanwhile, the last group proteins needed to verify the aforementioned concerns from the first and second groups.

In summary, this study found that the diverse LPxTG proteins fused with an N-terminal YSIRK-G/S signal peptide are encoded in the SS2 strain ZY05719. Consequently, two novel LPxTG proteins SspB and YzpA were verified to be exported to the extracellular space via a putative transport system that was dependent on YSIRK-G/S-directed translocation, and played vital roles during the infection of the SS2 strain ZY05719. A strategy for matching the diagnostic domain-architecture “N-terminal YSIRK-G/S motif and C-terminal LPxTG motif” was then adopted, thus discovering diverse potential effectors that were extensively distributed on Gram-positive bacteria. These findings have suggested that cell wall anchoring proteins that contain an N-terminal YSIRK-G/S motif are polymorphic effectors secreted by pathogenic bacteria, and may serve as therapeutic targets for counteracting or preventing bacterial infection by *Streptococcus* species.

## Supplementary Material

Supplemental Material
