# Supplementary material for: YSIRK-G/S-directed translocation is required for Streptococcus suis to deliver diverse cell wall anchoring effectors contributing to bacterial pathogenicity
Source: Virulence. 2020 Nov 2;11(1):1539–56. doi: 10.1080/21505594.2020.1838740 (PMC7644249; doi:10.1080/21505594.2020.1838740)
Supplement: Supplemental Material [file KVIR_A_1838740_SM7169.zip › Supplementary Figure S1-S2.docx]

**
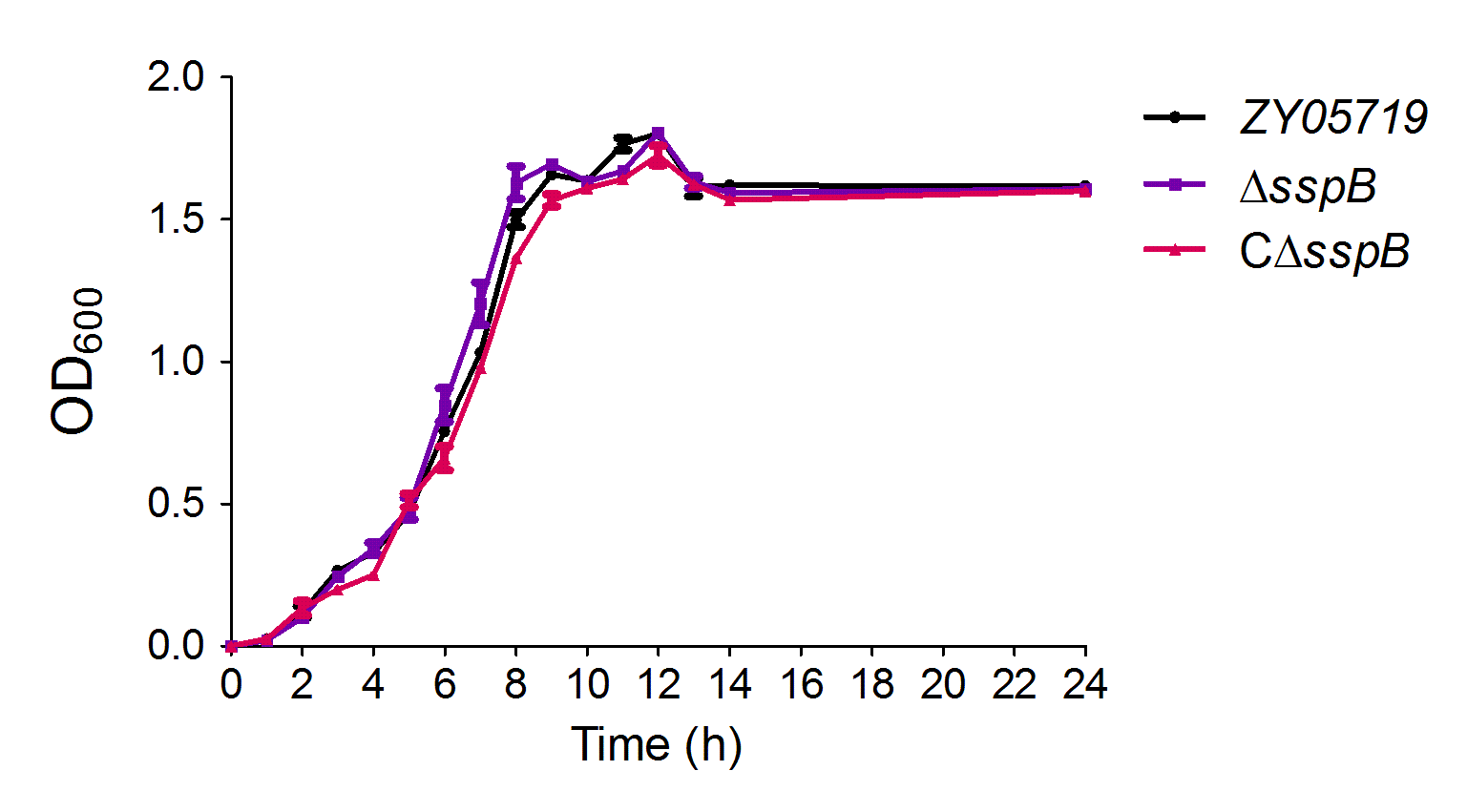
**

**Figure S1** The growth curves of strains ZY05719, ∆*sspB* and C∆*sspB.*


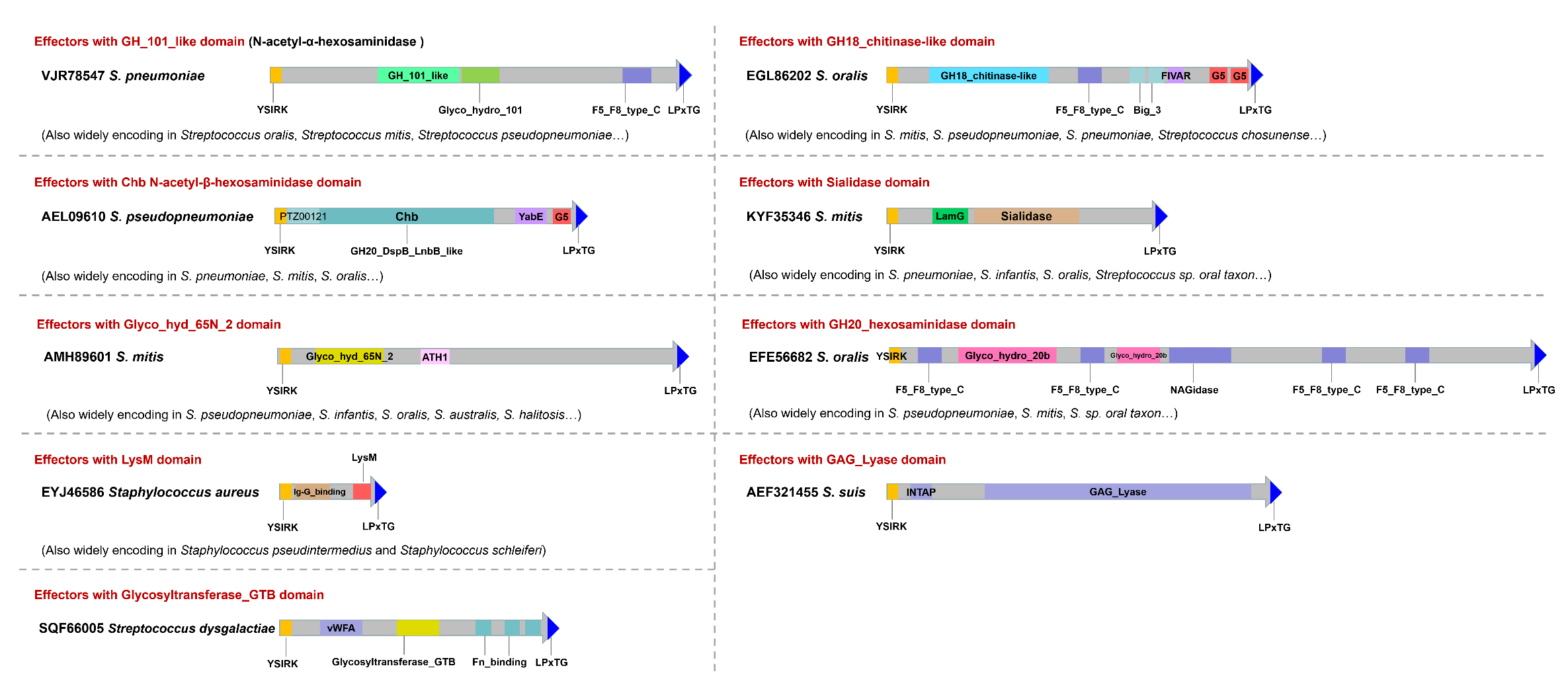


**Figure S2** Potential cell wall anchoring effectors of the putative YSIRK-related secretion pathway harbor diverse glycosyl hydrolase domains.
