# Supplementary material for: YSIRK-G/S-directed translocation is required for Streptococcus suis to deliver diverse cell wall anchoring effectors contributing to bacterial pathogenicity
Source: Virulence. 2020 Nov 2;11(1):1539–56. doi: 10.1080/21505594.2020.1838740 (PMC7644249; doi:10.1080/21505594.2020.1838740)
Supplement: Supplemental Material [file KVIR_A_1838740_SM7169.zip › Supplementary Table S1-S2.docx]

**Supplemental Table S1** Summary of bacterial strains and plasmids used for this study.

| Strains or plasmids | Strains or plasmids | Source or reference |
| --- | --- | --- |
| **Bacterial strains** |  |  |
| ZY05719 | The SS2 strain was isolated from a diseased pig | Our laboratory |
| A909 | Streptococcus agalactiae strain | Our laboratory |
| P1/7 | A virulent SS2 strain isolated from a diseased pig in Europe | Our laboratory |
| E. coli BL21(DE3) | For cloning the recombinant plasmids | Purchased from Vazyme |
| E. coli DH5α | For cloning the recombinant plasmids | Purchased from Vazyme |
| Δ*sspA*  Δ*sspB*  Δ*yzpA*  CΔ*sspA*  CΔ*sspB*  CΔ*yzpA*  ∆*ysirk*C∆*sspB*  ∆*ysirk*C*∆yzpA* | Deletion mutant of *sspA* with ZY05719 background  Deletion mutant of *sspB* with ZY05719 background  Deletion mutant of *yzpA* with ZY05719 background  Replenished of *sspA* with Δ*sspA* mutant background  Replenished of *sspB* with Δ*sspB* mutant background  Replenished of *yzpA* with Δ*yzpA* mutant background  Deletion of *ysirk* motif with CΔ*sspB* background  Deletion of *ysirk* motif with CΔ*yzpA* background | This study  This study  This study  This study  This study  This study  This study  This study |
| **Plasmid** |  |  |
| Pet21a | His-tag expressing vector, Amp^r^ | Purchased from Vazyme |

Amp^r^ , Ampicillin resistant.

**Supplemental Table S2** Primers used for PCR amplification.

| Primers | Primers sequence (5’-3’) |
| --- | --- |
| **For deletion** |  |
| Del-*sspA*-1 | CTCTCAAATTCATACCGTTCG |
| Del-*sspA*-2  Del-*sspA*-C2 | TCAGCATTATCCCATCTGCAGCTACCGATGGTG  TTGGCTTCCATCTGCAGCTACCGATGGTG |
| Del-*sspA*-3  Del-*sspA*-C3 | GGTAATCAGATTGAAGCCAAACCCTCAACCAAC  TGCAGATGGAAGCCAAACCCTCAACCAAC |
| Del-*sspA*-4 | GAAAGAACTTCCCGTGTAAAC |
| Del-*sspA*-5 | CGTAGGATATTTGAGGGAGAT |
| Del-*sspA*-6  Del-*sspB*-1  Del-*sspB*-2  Del-*sspB*-C2  Del-*sspB*-3  Del-*sspB*-C3  Del-*sspB*-4  Del-*sspB*-5  Del-*sspB*-6  Del-*yzpA*-1  Del-*yzpA*-2  Del-*yzpA*-C2  Del-*yzpA*-3  Del-*yzpA*-C3  Del-*yzpA*-4  Del-*yzpA*-5  Del-*yzpA*-6  ∆*ysirk*C∆*sspB*-F  ∆*ysirk*C∆*sspB*-R  ∆*ysirk*C∆*yzpA*-F  ∆*ysirk*C∆*yzpA*-R  Spc+SacB-F  Spc+SacB-R  **For** **complementation** strains  Complement-1  Complement-2  Complement-3  Complement-4  Complement-5  Complement-6  C∆*sspB*-F  C∆*sspB*-R  C∆*yzpA*-F  C∆yz*pA*-R  For checking  JC-SacB+Spc-F  JC-SacB+Spc-R  JC-*sspA*-F  JC-*sspA*-R  JC-*sspB*-F  JC-*sspB-*R  JC-*yzpA*-F  JC-*yzpA*-R  JC-∆*ysirk*C∆*sspB*-F  JC-∆*ysirk*C∆*sspB*-R  JC-∆*ysirk*C∆*yzpA*-F  JC-∆*ysirk*C∆*yzpA*-R  Pet21a-F  Pet21a-R  Pet21a-*sspA*-F  Pet21a-*sspA*-R  Pet21a-*sspB*-F  Pet21a-*sspB*-R  Pet21a-*yzpA*-F  Pet21a-*yzpA*-R | CAAGTCATCCTGACACCCCAC  GCCCTTCTAAAAAATCGAGTA  TCAGCATTATCCTGGTGGAGCTATGGCCCTCGG  TACAGTTGTGGTGGAGCTATGGCCCTCGG  GGTAATCAGATT CAACTGTAGACTCCAATCCTT  CTCCACCACAACTGTAGACTCCAATCCTT  AACCAGATAGGAGTCACGCAT  CTTCTAAAACACTTTCCATCG  ACCAGATAGGAGTCACGCATG  TGCATTCGATTTTGGTAAGGC  TCAGCATTATCCCGTATCTGTGCATGATAGTGG  TGGAAACCCGTATCTGTGCATGATAGTGG  GGTAATCAGATTGGTTTCCATATTCATCCCTGT  CAGATACGGGTTTCCATATTCATCCCTGT  ACATCGATGCCAAAACATTTG  TTCATAAGGTGCCTAAGTCCT  CATGACAAATAAACCCACTCC  GAGGCTTTTACTCAAGCAGGTGCACCATCGGTAG  CGGCATTATCTCATATTAGACTTCTTCTTTATGACG  **GAGGCTTTTACTCAA**AATATGGAAACCGCAATGG  CGGCATTATCTCATATTACTCACTTTCTGTTATCTT  GATAATGCTGAAAACTCCTTG  ATCTGATTACCAATTAGAATG  GTCTACTCGACTATCGATTGT  TTCAGCATTATCCAAATGATTGGGAACCGAGTAA  ACCCATCGAATTAGAACATATTTCAACAGGGTAG  ATACTATTTCTTGTGGGAATA  ACTTCAAATTCTTACTCGTTC  TATCTTTTTCAAAGCCGTAAC  GAGGCTTTTACTCAAATGAAACAGAAGTGGTCTC  CGGCATTATCTCATACTATTCTTTTCGCTTCAAATTTC  GAGGCTTTTACTCAAATGAATAGCAAGATTTTTTCGT  CGGCATTATCTCATATTACTCACTTTCTGTTATCT  ACTTTCTTTCCCTACCTCTAC  TCTTGCCAGTCACGTTACGTT  AAGCCTCATTTAGCTATCTCG  TAGAAATTCACCTCGGCAGT  CTTTCAATGTAAAACGCCCAT  TCCGATAGAATTGCACCGTTG  TAGGCATTAAGATGTATCGTT  ATACGAGCAAAGTTATTGGT  ATAAACAACGCTTTAGTATCA  ACTTTCTTTCCCTACCTCTAC  AAAGTAAGATGGGTTTAGTTT  TTTTGAACCTGCTAAACTCTC  TAATACGACTCACTATAGGG  TGCTAGTTATTGCTCAGCGG  AAATGGGTCGCGGATCCGAATTCACAAGCCTTGTAGAGACTAA  TGGTGGTGGTGGTGGTGCTCGAGGTAAATCGGCTCTTCTAGAAC  AAATGGGTCGCGGATCCGAATTCATCCGACCGACTCTTCATA  TGGTGGTGGTGGTGGTGCTCGAGCCTTTCTTTTGACCAGTCACC  AAATGGGTCGCGGATCCGAATTCATGGCTGAGGAGACTGACGCT  TGGTGGTGGTGGTGGTGCTCGAGGTCTTAGGCAGGCTTTGAACT |

Spc^r^, spectinomycin resistant
