# Supplementary material for: YSIRK-G/S-directed translocation is required for Streptococcus suis to deliver diverse cell wall anchoring effectors contributing to bacterial pathogenicity
Source: Virulence. 2020 Nov 2;11(1):1539–56. doi: 10.1080/21505594.2020.1838740 (PMC7644249; doi:10.1080/21505594.2020.1838740)
Supplement: Supplemental Material [file KVIR_A_1838740_SM7169.zip › Supplementary Table S3.docx]

| **Table S3** Potential cell wall anchoring effectors of the putative YSIRK-related secretion pathway screened by domain-architecture retrieval. | | |
| --- | --- | --- |
| Sequences | Taxonomy | Domain Names |
| 1956 | Streptococcus | YSIRK_signal~GH_101_like~Glyco_hyd_101C~F5_F8_type_C~Gram_pos_anchor |
| 1494 | Streptococcus | YSIRK_signal~MSCRAMM_SdrC~GH18_chitinase-like~F5_F8_type_C~Big_3~FIVAR~G5~Gram_pos_anchor |
| 794 | Streptococcus | YSIRK_signal~PTZ00121~Chb~G5~Gram_pos_anchor |
| 656 | Streptococcus pneumoniae | YSIRK_signal~RICH~PRK10819~Gram_pos_anchor |
| 484 | Lactobacillales | YSIRK_signal~Smc~Gram_pos_anchor |
| 329 | Streptococcus | YSIRK_signal~PTZ00121~Chb~G5~SucB_Actino~Gram_pos_anchor |
| 316 | Streptococcus pneumoniae | YSIRK_signal~RICH~PRK10263~Gram_pos_anchor |
| 289 | Streptococcus pyogenes | YSIRK_signal~vWFA~Fn_bind~Gram_pos_anchor |
| 273 | Streptococcus pneumoniae | YSIRK_signal~MSCRAMM_SdrC~GH18_chitinase-like~F5_F8_type_C~Big_3~CALCOCO1~FIVAR~G5~Gram_pos_anchor |
| 237 | Bacilli | YSIRK_signal~Gram_pos_anchor |
| 225 | Lactobacillales | YSIRK_signal~PHA03247~Gram_pos_anchor |
| 201 | Streptococcus pneumoniae | YSIRK_signal~PRK11633~PTZ00121~Chb~G5~Gram_pos_anchor |
| 193 | Lactobacillales | YSIRK_signal~SMC_prok_B~Gram_pos_anchor |
| 182 | Streptococcus | YSIRK_signal~AlphaC_N~AlphaC_C~Rib~Gram_pos_anchor |
| 179 | Streptococcus | YSIRK_signal~Peptidases_S8_S53~PA~fn3_5~Gram_pos_anchor |
| 177 | Streptococcus | YSIRK_signal~MSCRAMM_SdrC~Peptidases_S8_S53~PA~fn3_5~FlgD_ig~FIVAR~pullulan_Gpos~Gram_pos_anchor |
| 151 | Streptococcus | YSIRK_signal~MSCRAMM_SdrC~Chb~G5~Gram_pos_anchor |
| 126 | Streptococcus | YSIRK_signal~Tryp_SPc~GH_101_like~Glyco_hyd_101C~F5_F8_type_C~Gram_pos_anchor |
| 123 | Bacilli | YSIRK_signal~PTZ00121~Gram_pos_anchor |
| 116 | Streptococcus | YSIRK_signal~LamG~Sialidase~Gram_pos_anchor |
| 89 | Streptococcus pyogenes | YSIRK_signal~MSCRAMM_SdrC~Peptidases_S8_S53~PA~fn3_5~Gram_pos_anchor |
| 78 | Streptococcus | YSIRK_signal~SucB_Actino~Trypan_PARP~Gram_pos_anchor |
| 72 | Streptococcus pneumoniae | YSIRK_signal~RICH~PHA03247~Gram_pos_anchor |
| 72 | Streptococcus pneumoniae | YSIRK_signal~PTZ00121~Chb~G5~TonB_N~Gram_pos_anchor |
| 71 | Streptococcus pneumoniae | YSIRK_signal~RICH~PRK10819~**predic_Ig_block**~Gram_pos_anchor |
| 67 | Bacilli | YSIRK_signal~rne~Gram_pos_anchor |
| 66 | Streptococcus | YSIRK_signal~PRK11633~F5_F8_type_C~Glyco_hydro_20b~GH20_hexosaminidase~F5_F8_type_C~Glyco_hydro_20b~NAGidase~F5_F8_type_C~Gram_pos_anchor |
| 62 | Streptococcus pneumoniae | YSIRK_signal~PRK11633~PTZ00121~Chb~G5~SucB_Actino~Gram_pos_anchor |
| 56 | Streptococcus | YSIRK_signal~MSCRAMM_SdrC~Chb~G5~SucB_Actino~Gram_pos_anchor |
| 56 | Terrabacteria group | YSIRK_signal~MSCRAMM_SdrC~Gram_pos_anchor |
| 53 | Bacilli | YSIRK_signal~MSCRAMM_SdrC~**CshA_fibril_rpt**~Gram_pos_anchor |
| 53 | Streptococcus pyogenes | YSIRK_signal~PRK09039~Gram_pos_anchor |
| 48 | Streptococcus pneumoniae | YSIRK_signal~RICH~PRK14948~RICH~Gram_pos_anchor |
| 46 | Streptococcus pneumoniae | YSIRK_signal~RICH~PRK10263~**predic_Ig_block**~Gram_pos_anchor |
| 44 | Streptococcus pyogenes | YSIRK_signal~PRK07003~Gram_pos_anchor |
| 41 | Streptococcus | YSIRK_signal~LacZ~Big_4~G5~Gram_pos_anchor |
| 41 | Staphylococcus | YSIRK_signal~MSCRAMM_SdrC~MG1~Gram_pos_anchor |
| 40 | Lactobacillales | YSIRK_signal~MSCRAMM_SdrC~MucBP~Gram_pos_anchor |
| 38 | Streptococcus | YSIRK_signal~MSCRAMM_SdrD~GH18_chitinase-like~F5_F8_type_C~Big_3~FIVAR~G5~Gram_pos_anchor |
| 37 | Staphylococcus | YSIRK_signal~MSCRAMM_SdrC~SasC_Mrp_aggreg~DUF1542~PRK05035~PTZ00121~Gram_pos_anchor |
| 36 | Streptococcus pneumoniae | YSIRK_signal~RICH~PRK11633~PRK10819~Gram_pos_anchor |
| 35 | Streptococcus | YSIRK_signal~PTZ00121~GbpC~ProTailRpt~Gram_pos_anchor |
| 33 | Staphylococcus aureus | YSIRK_signal~TonB~AlphaC_N~Gram_pos_anchor |
| 33 | Firmicutes | YSIRK_signal~MSCRAMM_SdrC~LamG~Sialidase~Gram_pos_anchor |
| 31 | Streptococcus | YSIRK_signal~**CshA_fibril_rpt**~repeat_SSSPR51~Gram_pos_anchor |
| 28 | Streptococcus | YSIRK_signal~**CshA_fibril_rpt**~Gram_pos_anchor |
| 28 | Lactobacillales | YSIRK_signal~MucBP~Gram_pos_anchor |
| 28 | Streptococcus pneumoniae | YSIRK_signal~PTZ00121~RICH~CCDC158~RICH~PHA03247~Gram_pos_anchor |
| 27 | Streptococcus pneumoniae | YSIRK_signal~PTZ00121~Chb~Gram_pos_anchor |
| 27 | Streptococcus agalactiae | YSIRK_signal~SucB_Actino~PRK10819~Trypan_PARP~Gram_pos_anchor |
| 27 | Streptococcus pneumoniae | YSIRK_signal~RICH~Smc~RICH~PHA03247~Gram_pos_anchor |
| 27 | Streptococcus pyogenes | YSIRK_signal~APG6~Smc~Gram_pos_anchor |
| 26 | Streptococcus | YSIRK_signal~TonB~Chb~G5~Gram_pos_anchor |
| 25 | Streptococcus agalactiae | YSIRK_signal~SMC_N~GAGBD~RICH~PTZ00449~Gram_pos_anchor |
| 25 | Streptococcus | YSIRK_signal~MSCRAMM_SdrC~Peptidases_S8_S53~PA~fn3_5~FlgD_ig~PRK11907~Gram_pos_anchor |
| 25 | Streptococcus | YSIRK_signal~rne~GH18_chitinase-like~F5_F8_type_C~Big_3~FIVAR~G5~Gram_pos_anchor |
| 24 | Streptococcus pyogenes | YSIRK_signal~PHA03307~Gram_pos_anchor |
| 23 | Streptococcus pyogenes | YSIRK_signal~PTZ00121~PRK09039~Gram_pos_anchor |
| 23 | Streptococcus equi | YSIRK_signal~PRK12704~SMC_prok_B~Gram_pos_anchor |
| 21 | Lactobacillales | YSIRK_signal~Rib~Gram_pos_anchor |
| 21 | Staphylococcus | YSIRK_signal~rne~MSCRAMM_SdrC~SdrG_C_C~pullulan_Gpos~Gram_pos_anchor |
| 21 | Streptococcus suis | YSIRK_signal~MSCRAMM_SdrC~Peptidases_S8_S53~PA~fn3_5~FlgD_ig~FLgD_tudor~FIVAR~pullulan_Gpos~Gram_pos_anchor |
| 21 | Streptococcus pneumoniae | YSIRK_signal~GAGBD~Aim21~Trypan_PARP~Gram_pos_anchor |
| 20 | Streptococcus | YSIRK_signal~MSCRAMM_SdrC~Glyco_hyd_65N_2~ATH1~Gram_pos_anchor |
| 20 | Streptococcus | YSIRK_signal~MSCRAMM_SdrC~SbcC~GbpC~ProTailRpt~Gram_pos_anchor |
| 19 | Streptococcus | YSIRK_signal~DUF1542~PTZ00121~Gram_pos_anchor |
| 19 | Streptococcus equi | YSIRK_signal~Peptidase_S46~SMC_prok_B~Gram_pos_anchor |
| 19 | Streptococcus | YSIRK_signal~PTZ00121~GH18_chitinase-like~F5_F8_type_C~Big_3~FIVAR~G5~Gram_pos_anchor |
| 19 | Streptococcus pneumoniae | YSIRK_signal~RICH~predic_Ig_block~Gram_pos_anchor |
| 18 | Streptococcus | YSIRK_signal~rne~Chb~G5~Gram_pos_anchor |
| 18 | Streptococcus pneumoniae | YSIRK_signal~PTZ00121~RICH~PHA03247~Gram_pos_anchor |
| 18 | Bacilli | YSIRK_signal~Trypan_PARP~Gram_pos_anchor |
| 18 | Streptococcus suis | YSIRK_signal~MucBP~PRK15313~repeat_SSSPR51~Gram_pos_anchor |
| 18 | Streptococcus | YSIRK_signal~MucBP~Trypan_PARP~Gram_pos_anchor |
| 18 | Staphylococcus | YSIRK_signal~B~**LysM**~Gram_pos_anchor |
| 17 | Bacilli | YSIRK_signal~PRK13108~Gram_pos_anchor |
| 17 | Lactobacillales | YSIRK_signal~MSCRAMM_SdrC~Rib~Gram_pos_anchor |
| 17 | Bacilli | YSIRK_signal~MSCRAMM_SdrD~Gram_pos_anchor |
| 16 | Streptococcus | YSIRK_signal~**lectin_L-type**~FIVAR~Gram_pos_anchor |
| 16 | Streptococcus pyogenes | YSIRK_signal~PHA03255~vWFA~Fn_bind~Gram_pos_anchor |
| 16 | Streptococcus suis | YSIRK_signal~MSCRAMM_SdrC~MucBP~PTZ00441~repeat_SSSPR51~MucBP~repeat_SSSPR51~Gram_pos_anchor |
| 15 | Streptococcus | YSIRK_signal~Trypan_PARP~F5_F8_type_C~Glyco_hydro_20b~GH20_hexosaminidase~F5_F8_type_C~Glyco_hydro_20b~NAGidase~F5_F8_type_C~Gram_pos_anchor |
| 15 | Streptococcus pneumoniae | YSIRK_signal~RICH~predic_Ig_block~PRK10819~Gram_pos_anchor |
| 15 | Streptococcus | YSIRK_signal~Spc7~SMC_prok_B~Gram_pos_anchor |
| 15 | Streptococcus | YSIRK_signal~lectin_L-type~Gram_pos_anchor |
| 14 | Streptococcus | YSIRK_signal~rne~Glyco_hyd_65N_2~ATH1~FN3~Gram_pos_anchor |
| 14 | Streptococcus | YSIRK_signal~rne~CshA_fibril_rpt~repeat_SSSPR51~Gram_pos_anchor |
| 14 | Streptococcus | YSIRK_signal~GH18_chitinase-like~F5_F8_type_C~Big_3~FIVAR~G5~Gram_pos_anchor |
| 14 | Streptococcus | YSIRK_signal~MSCRAMM_SdrC~Glyco_hyd_65N_2~ATH1~PHA03378~Gram_pos_anchor |
| 14 | Staphylococcaceae | YSIRK_signal~MDN1~MSCRAMM_SdrD~He_PIG~Gram_pos_anchor |
| 14 | Streptococcus pneumoniae | YSIRK_signal~RICH~pullulan_Gpos~Gram_pos_anchor |
| 13 | Bacilli | YSIRK_signal~He_PIG~Gram_pos_anchor |
| 13 | Streptococcus | YSIRK_signal~DUF4775~GH18_chitinase-like~F5_F8_type_C~Big_3~FIVAR~G5~Gram_pos_anchor |
| 12 | Streptococcus | YSIRK_signal~TonB~GH18_chitinase-like~F5_F8_type_C~Big_3~FIVAR~G5~Gram_pos_anchor |
| 12 | Streptococcus pyogenes | YSIRK_signal~Smc~Phage_capsid~Gram_pos_anchor |
| 12 | Staphylococcus | YSIRK_signal~MSCRAMM_SdrC~SasC_Mrp_aggreg~DUF1542~SMC_prok_B~DUF1542~Gram_pos_anchor |
| 12 | Streptococcus | YSIRK_signal~PTZ00121~GAGBD~RICH~PTZ00449~Gram_pos_anchor |
| 11 | Streptococcus sanguinis | YSIRK_signal~DUF1542~PTZ00121~Herpes_BLLF1~Gram_pos_anchor |
| 11 | Streptococcus | YSIRK_signal~MSCRAMM_SdrC~Peptidases_S8_S53~PA~fn3_5~FLgD_tudor~FIVAR~pullulan_Gpos~Gram_pos_anchor |
| 11 | Streptococcus suis | YSIRK_signal~MSCRAMM_SdrC~MucBP~MISS~repeat_SSSPR51~MucBP~repeat_SSSPR51~Gram_pos_anchor |
| 11 | Staphylococcus | YSIRK_signal~PRK11281~PTZ00121~Gram_pos_anchor |
| 11 | Staphylococcus aureus | YSIRK_signal~PTZ00121~lectin_L-type~G5~SasG_E~G5~SasG_E~G5~SasG_E~Gram_pos_anchor |
| 11 | Streptococcus agalactiae | YSIRK_signal~Rib~He_PIG~Rib~Gram_pos_anchor |
| 11 | Staphylococcus chromogenes | YSIRK_signal~rne~Rib~PRK15316~Gram_pos_anchor |
| 11 | Bacilli | YSIRK_signal~MSCRAMM_SdrD~PTZ00449~Gram_pos_anchor |
| 11 | Streptococcus | YSIRK_signal~SucB_Actino~PRK10819~Gram_pos_anchor |
| 11 | Staphylococcus aureus | YSIRK_signal~PTZ00121~lectin_L-type~G5~SasG_E~G5~SasG_E~G5~SasG_E~G5~SasG_E~Gram_pos_anchor |
| 10 | Lactobacillales | YSIRK_signal~G5~Gram_pos_anchor |
| 10 | Streptococcus oralis | YSIRK_signal~PTZ00121~CshA_fibril_rpt~repeat_SSSPR51~Gram_pos_anchor |
| 10 | Streptococcus | YSIRK_signal~SMC_prok_A~Gram_pos_anchor |
| 10 | Lactobacillales | YSIRK_signal~pullulan_Gpos~Gram_pos_anchor |
| 10 | Firmicutes | YSIRK_signal~Rib~hyperosmo_Ebh~Rib~Gram_pos_anchor |
| 9 | Streptococcus | YSIRK_signal~Cornifin~Gram_pos_anchor |
| 9 | Streptococcus | YSIRK_signal~MSCRAMM_SdrC~Glyco_hyd_65N_2~ATH1~FN3~Gram_pos_anchor |
| 9 | Bacilli | YSIRK_signal~IsdH_HarA~IsdB~Gram_pos_anchor |
| 9 | Staphylococcus | YSIRK_signal~PRK08581~MSCRAMM_SdrC~SdrG_C_C~Gram_pos_anchor |
| 9 | Streptococcus pneumoniae | YSIRK_signal~RICH~PTZ00121~RICH~Gram_pos_anchor |
| 9 | Streptococcus | YSIRK_signal~MSCRAMM_SdrD~LamG~Sialidase~Gram_pos_anchor |
| 9 | Streptococcus iniae | YSIRK_signal~PRK00708~Gram_pos_anchor |
| 8 | Staphylococcus capitis | YSIRK_signal~IsdH_HarA~Gram_pos_anchor |
| 8 | Streptococcus | YSIRK_signal~Smc~GbpC~PTZ00449~Gram_pos_anchor |
| 8 | Streptococcus | YSIRK_signal~LamG~Sialidase~rne~Gram_pos_anchor |
| 8 | Streptococcus agalactiae | YSIRK_signal~hyperosmo_Ebh~Rib~He_PIG~Rib~Gram_pos_anchor |
| 8 | Streptococcus sanguinis | YSIRK_signal~PRK13108~CshA_fibril_rpt~Gram_pos_anchor |
| 8 | Streptococcus suis | YSIRK_signal~rne~MucBP~MISS~repeat_SSSPR51~MucBP~repeat_SSSPR51~Gram_pos_anchor |
| 8 | Streptococcus | YSIRK_signal~PTZ00108~Trypan_PARP~Gram_pos_anchor |
| 8 | Staphylococcus | YSIRK_signal~MSCRAMM_SdrC~PTZ00121~Gram_pos_anchor |
| 8 | Staphylococcus | YSIRK_signal~MDN1~PTZ00121~Gram_pos_anchor |
| 8 | Streptococcus | YSIRK_signal~SucB_Actino~PRK10819~SucB_Actino~Gram_pos_anchor |
| 7 | Streptococcus suis | YSIRK_signal~MSCRAMM_SdrC~MucBP~MISS~repeat_SSSPR51~MucBP~repeat_SSSPR51~MucBP~repeat_SSSPR51~Gram_pos_anchor |
| 7 | Staphylococcus | YSIRK_signal~MIP-T3~lectin_L-type~MucBP~Gram_pos_anchor |
| 7 | Staphylococcus agnetis | YSIRK_signal~MSCRAMM_SdrC~rne~Gram_pos_anchor |
| 7 | Streptococcus | YSIRK_signal~Peptidases_S8_S53~PA~fn3_5~FlgD_ig~Gram_pos_anchor |
| 7 | Streptococcus agalactiae | YSIRK_signal~PTZ00108~Trypan_PARP~PRK10819~SucB_Actino~Gram_pos_anchor |
| 7 | Streptococcus | YSIRK_signal~PTZ00121~GbpC~Aim21~Gram_pos_anchor |
| 7 | Streptococcus parauberis | YSIRK_signal~ligA~Gram_pos_anchor |
| 7 | Streptococcus pyogenes | YSIRK_signal~Lamp~PHA03247~Gram_pos_anchor |
| 7 | Streptococcus agalactiae | YSIRK_signal~Neisseria_TspB~Trypan_PARP~Gram_pos_anchor |
| 7 | Staphylococcus simulans | YSIRK_signal~PRK14949~MSCRAMM_SdrC~SdrG_C_C~pullulan_Gpos~Gram_pos_anchor |
| 7 | Lactobacillus acidophilus | YSIRK_signal~MSCRAMM_SdrC~DUF285~LRR_5~DUF285~TonB~Gram_pos_anchor |
| 7 | Streptococcus | YSIRK_signal~MSCRAMM_SdrC~LamG~Sialidase~rne~Gram_pos_anchor |
| 7 | Streptococcus pneumoniae | YSIRK_signal~RICH~Trypan_PARP~Gram_pos_anchor |
| 6 | Lactobacillales | YSIRK_signal~rne~pullulan_Gpos~AmyAc_family~pullulan_Gpos~Gram_pos_anchor |
| 6 | Streptococcus | YSIRK_signal~PTZ00121~GbpC~PTZ00449~Gram_pos_anchor |
| 6 | Bacillales | YSIRK_signal~TonB~Gram_pos_anchor |
| 6 | Streptococcus suis | YSIRK_signal~MucBP~PHA03247~repeat_SSSPR51~Gram_pos_anchor |
| 6 | Streptococcus suis | YSIRK_signal~MSCRAMM_SdrC~MucBP~PHA03247~repeat_SSSPR51~Gram_pos_anchor |
| 6 | Streptococcus | YSIRK_signal~PRK07003~CshA_fibril_rpt~Gram_pos_anchor |
| 6 | Streptococcus suis | YSIRK_signal~rne~MucBP~MISS~repeat_SSSPR51~MucBP~repeat_SSSPR51~MucBP~repeat_SSSPR51~MucBP~repeat_SSSPR51~Gram_pos_anchor |
| 6 | Streptococcus agalactiae | YSIRK_signal~PTZ00108~PRK10819~Trypan_PARP~Gram_pos_anchor |
| 6 | Streptococcus agalactiae | YSIRK_signal~PTZ00121~Trypan_PARP~Gram_pos_anchor |
| 6 | Streptococcus | YSIRK_signal~Tryp_SPc~AfuC~Calx-beta~Mitofilin~G5~Gram_pos_anchor |
| 6 | Streptococcus oralis | YSIRK_signal~PRK11633~F5_F8_type_C~Glyco_hydro_20b~GH20_hexosaminidase~F5_F8_type_C~Glyco_hydro_20b~NAGidase~Glyco_hydro_106~F5_F8_type_C~Gram_pos_anchor |
| 6 | Staphylococcus | YSIRK_signal~MDN1~MSCRAMM_SdrC~Gram_pos_anchor |
| 6 | Staphylococcus | YSIRK_signal~MSCRAMM_SdrC~SasC_Mrp_aggreg~DUF1542~PTZ00121~Gram_pos_anchor |
| 6 | Streptococcus | YSIRK_signal~MSCRAMM_SdrC~GH18_chitinase-like~F5_F8_type_C~Big_3~FIVAR~HOOK~G5~Gram_pos_anchor |
| 6 | Streptococcus pneumoniae | YSIRK_signal~MSCRAMM_SdrC~GH18_chitinase-like~Big_3~FIVAR~G5~Gram_pos_anchor |
| 6 | Streptococcus | YSIRK_signal~MSCRAMM_SdrC~LacZ~Big_4~G5~Gram_pos_anchor |
| 6 | Streptococcus sanguinis | YSIRK_signal~DUF1542~PTZ00121~PHA03381~Gram_pos_anchor |
| 6 | Staphylococcus haemolyticus | YSIRK_signal~MSCRAMM_SdrC~SasC_Mrp_aggreg~PTZ00121~DUF1542~Gram_pos_anchor |
| 6 | Streptococcus | YSIRK_signal~DUF4775~Tryp_SPc~AfuC~Calx-beta~Mitofilin~G5~Gram_pos_anchor |
| 5 | Streptococcus pneumoniae | YSIRK_signal~PTZ00121~RICH~PRK10819~Gram_pos_anchor |
| 5 | Lactobacillales | YSIRK_signal~FIVAR~Gram_pos_anchor |
| 5 | Streptococcus suis | YSIRK_signal~PRK05035~MucBP~PTZ00441~repeat_SSSPR51~MucBP~repeat_SSSPR51~MucBP~repeat_SSSPR51~Gram_pos_anchor |
| 5 | Streptococcus | YSIRK_signal~Tryp_SPc~AfuC~Calx-beta~DUF1542~PHA03247~G5~Gram_pos_anchor |
| 5 | Staphylococcus | YSIRK_signal~NEAT~Gram_pos_anchor |
| 5 | Streptococcus pneumoniae | YSIRK_signal~PTZ00121~GH18_chitinase-like~F5_F8_type_C~Big_3~CALCOCO1~FIVAR~G5~Gram_pos_anchor |
| 5 | Streptococcus pneumoniae | YSIRK_signal~RICH~tolA~PRK14948~RICH~Gram_pos_anchor |
| 5 | Streptococcus pneumoniae | YSIRK_signal~PTZ00121~RICH~PRK14948~RICH~Gram_pos_anchor |
| 5 | Lactobacillales | YSIRK_signal~PRK10819~Gram_pos_anchor |
| 5 | Streptococcus sanguinis | YSIRK_signal~COG5665~CshA_fibril_rpt~Gram_pos_anchor |
| 5 | Streptococcus cristatus | YSIRK_signal~MSCRAMM_SdrC~Chb~G5~Rib_recp_KP_reg~Gram_pos_anchor |
| 5 | Streptococcus pneumoniae | YSIRK_signal~RICH~PTZ00121~Gram_pos_anchor |
| 5 | Streptococcus gordonii | YSIRK_signal~PRK14951~CshA_fibril_rpt~Gram_pos_anchor |
| 5 | Bacilli | YSIRK_signal~PTZ00341~Gram_pos_anchor |
| 5 | Bacilli | YSIRK_signal~PRK11907~Gram_pos_anchor |
| 5 | Streptococcus suis | YSIRK_signal~MSCRAMM_SdrC~Peptidases_S8_S53~PA~fn3_5~FIVAR~pullulan_Gpos~Gram_pos_anchor |
| 5 | Streptococcus pneumoniae | YSIRK_signal~GAGBD~Aim21~Gram_pos_anchor |
| 5 | Lactobacillales | YSIRK_signal~DUF1542~Gram_pos_anchor |
| 5 | Staphylococcus | YSIRK_signal~MSCRAMM_SdrC~SasC_Mrp_aggreg~DUF1542~PRK05035~PTZ00121~DUF1542~Gram_pos_anchor |
| 5 | Bacilli | YSIRK_signal~MSCRAMM_SdrC~PHA03247~Gram_pos_anchor |
| 5 | Streptococcus oralis | YSIRK_signal~Glyco_hyd_65N_2~ATH1~FN3~Gram_pos_anchor |
| 5 | Lactobacillus salivarius | YSIRK_signal~MucBP~PRK11907~Gram_pos_anchor |
| 5 | Streptococcus parasanguinis | YSIRK_signal~Remorin_C~GbpC~Gram_pos_anchor |
| 4 | Lactobacillales | YSIRK_signal~MucBP~repeat_SSSPR51~Gram_pos_anchor |
| 4 | Streptococcus | YSIRK_signal~PRK11907~Smc~GbpC~ProTailRpt~Gram_pos_anchor |
| 4 | Lactobacillus gasseri | YSIRK_signal~MSCRAMM_SdrC~DUF3664~Gram_pos_anchor |
| 4 | Streptococcus pneumoniae | YSIRK_signal~MSCRAMM_SdrC~Chb~G5~TonB_N~Gram_pos_anchor |
| 4 | Streptococcus | YSIRK_signal~PTZ00121~Chb~YabE~G5~SucB_Actino~Gram_pos_anchor |
| 4 | Streptococcus suis | YSIRK_signal~INTAP~GAG_Lyase~IsdB~Gram_pos_anchor |
| 4 | Staphylococcus aureus | YSIRK_signal~AlphaC_N~Gram_pos_anchor |
| 4 | Staphylococcus | YSIRK_signal~MDN1~Gram_pos_anchor |
| 4 | Lactobacillus delbrueckii | YSIRK_signal~PRK10856~PRK07003~Gram_pos_anchor |
| 4 | Streptococcus | YSIRK_signal~MSCRAMM_SdrC~F5_F8_type_C~Glyco_hydro_20b~GH20_hexosaminidase~F5_F8_type_C~Glyco_hydro_20b~NAGidase~F5_F8_type_C~rne~Gram_pos_anchor |
| 4 | Streptococcus | YSIRK_signal~MSCRAMM_SdrC~GH_101_like~Glyco_hyd_101C~F5_F8_type_C~Gram_pos_anchor |
| 4 | Streptococcus suis | YSIRK_signal~rne~MucBP~PTZ00441~repeat_SSSPR51~MucBP~repeat_SSSPR51~MucBP~repeat_SSSPR51~Gram_pos_anchor |
| 4 | Streptococcus azizii | YSIRK_signal~CshA_fibril_rpt~hyperosmo_Ebh~DUF1542~PTZ00121~DUF1542~Gram_pos_anchor |
| 4 | Gemella | YSIRK_signal~SSURE~Gram_pos_anchor |
| 4 | Streptococcus agalactiae | YSIRK_signal~PTZ00108~PRK10819~SucB_Actino~Gram_pos_anchor |
| 4 | Streptococcus | YSIRK_signal~MSCRAMM_SdrC~Chb~G5~PDHac_trf_long~Gram_pos_anchor |
| 4 | Staphylococcus | YSIRK_signal~PHA03169~MSCRAMM_SdrC~Gram_pos_anchor |
| 4 | Streptococcus | YSIRK_signal~MSCRAMM_SdrC~Glyco_hyd_65N_2~ATH1~FN3~PRK10263~Gram_pos_anchor |
| 4 | Bacilli | YSIRK_signal~Herpes_BLLF1~Gram_pos_anchor |
| 4 | Lactobacillus salivarius | YSIRK_signal~CBP_CCPA~Gram_pos_anchor |
| 4 | Clostridiales | YSIRK_signal~hyperosmo_Ebh~Rib~Gram_pos_anchor |
| 4 | Streptococcus uberis | YSIRK_signal~Collagen~RPT_S_cricet~Gram_pos_anchor |
| 4 | Streptococcus agalactiae | YSIRK_signal~PRK10819~Trypan_PARP~Gram_pos_anchor |
| 4 | Staphylococcus | YSIRK_signal~rne~MSCRAMM_SdrC~SdrG_C_C~MSCRAMM_SdrC~Gram_pos_anchor |
| 4 | Streptococcus | YSIRK_signal~PTZ00121~PRK10819~Gram_pos_anchor |
| 4 | Streptococcus | YSIRK_signal~GAG_Lyase~Gram_pos_anchor |
| 4 | Streptococcus pneumoniae | YSIRK_signal~RICH~PRK14948~predic_Ig_block~Gram_pos_anchor |
| 4 | Streptococcus | YSIRK_signal~vWFA~Gram_pos_anchor |
| 4 | Lactobacillus | YSIRK_signal~GbpC~Gram_pos_anchor |
| 4 | Streptococcus suis | YSIRK_signal~VirB10~MucBP~pullulan_Gpos~Gram_pos_anchor |
| 4 | Streptococcus | YSIRK_signal~PRK08581~SbcC~GAGBD~Pro-rich~Gram_pos_anchor |
| 4 | Streptococcus | YSIRK_signal~Smc~GbpC~Gram_pos_anchor |
| 4 | Streptococcus | YSIRK_signal~rne~Glyco_hyd_65N_2~ATH1~FN3~rne~Gram_pos_anchor |
| 4 | Streptococcus suis | YSIRK_signal~rne~MucBP~MISS~repeat_SSSPR51~MucBP~repeat_SSSPR51~MucBP~repeat_SSSPR51~Gram_pos_anchor |
| 4 | Streptococcus gordonii | YSIRK_signal~PRK13335~CshA_fibril_rpt~Gram_pos_anchor |
| 4 | Streptococcus | YSIRK_signal~MSCRAMM_SdrC~Glyco_hyd_65N_2~ATH1~PRK10263~Gram_pos_anchor |
| 4 | Streptococcus canis | YSIRK_signal~F5_F8_type_C~LamG~Gram_pos_anchor |
| 4 | Streptococcus | YSIRK_signal~pullulan_Gpos~Rib~Gram_pos_anchor |
| 4 | Staphylococcus hyicus | YSIRK_signal~MSCRAMM_SdrD~SdrG_C_C~PTZ00341~PRK08581~Gram_pos_anchor |
| 4 | Bacilli | YSIRK_signal~rne~Rib~Gram_pos_anchor |
| 3 | Streptococcus equi | YSIRK_signal~GA-like~IgG_binding_B~Gram_pos_anchor |
| 3 | Streptococcus | YSIRK_signal~MucBP~pullulan_Gpos~Gram_pos_anchor |
| 3 | Streptococcus sobrinus | YSIRK_signal~PTZ00121~GbpC~PTZ00449~Aim21~Gram_pos_anchor |
| 3 | Lactobacillus | YSIRK_signal~DUF285~Gram_pos_anchor |
| 3 | Streptococcus pneumoniae | YSIRK_signal~RICH~PRK14950~predic_Ig_block~Gram_pos_anchor |
| 3 | Staphylococcus sciuri | YSIRK_signal~DUF4775~lectin_L-type~MucBP~Gram_pos_anchor |
| 3 | Streptococcus cristatus | YSIRK_signal~DUF1542~BshC~Gram_pos_anchor |
| 3 | Streptococcus | YSIRK_signal~Tryp_SPc~AfuC~Calx-beta~NESP55~PTZ00449~G5~Gram_pos_anchor |
| 3 | Staphylococcus condimenti | YSIRK_signal~PTZ00341~MSCRAMM_SdrC~SdrG_C_C~Gram_pos_anchor |
| 3 | Streptococcus agalactiae | YSIRK_signal~Neisseria_TspB~PRK10819~Trypan_PARP~Gram_pos_anchor |
| 3 | Streptococcus mitis | YSIRK_signal~MSCRAMM_SdrC~Rib~repeat_SSSPR51~Gram_pos_anchor |
| 3 | Streptococcus equi | YSIRK_signal~APG6~SMC_prok_B~Gram_pos_anchor |
| 3 | Lactobacillus | YSIRK_signal~PRK13042~Gram_pos_anchor |
| 3 | Streptococcus agalactiae | YSIRK_signal~AlphaC_N~AlphaC_C~Gram_pos_anchor |
| 3 | Staphylococcus | YSIRK_signal~PRK14949~MSCRAMM_SdrC~SdrG_C_C~Gram_pos_anchor |
| 3 | Bacilli | YSIRK_signal~DUF4775~Rib~Gram_pos_anchor |
| 3 | Lactobacillus salivarius | YSIRK_signal~CBP_CCPA~PHA03247~Gram_pos_anchor |
| 3 | Streptococcus pneumoniae | YSIRK_signal~RICH~PTZ00121~PRK14948~RICH~Gram_pos_anchor |
| 3 | Lactobacillus | YSIRK_signal~Peptidase_M26_C~Gram_pos_anchor |
| 3 | Lactobacillus reuteri | YSIRK_signal~PRK10856~Rib~Gram_pos_anchor |
| 3 | Streptococcus merionis | YSIRK_signal~PTZ00121~GAGBD~G5~Gram_pos_anchor |
| 3 | Streptococcus parasanguinis | YSIRK_signal~TolA~GbpC~Gram_pos_anchor |
| 3 | Streptococcus | YSIRK_signal~tolA_full~GbpC~PTZ00449~Gram_pos_anchor |
| 3 | Staphylococcus | YSIRK_signal~PRK08581~MSCRAMM_SdrC~Gram_pos_anchor |
| 3 | Streptococcus suis | YSIRK_signal~SbcC~GA~FIVAR~hyperosmo_Ebh~rne~hyperosmo_Ebh~rne~MSCRAMM_SdrC~termin_org_DnaJ~hyperosmo_Ebh~Gram_pos_anchor |
| 3 | Lactobacillales | YSIRK_signal~PRK08026~Gram_pos_anchor |
| 3 | Staphylococcus haemolyticus | YSIRK_signal~rne~MSCRAMM_SdrC~Gram_pos_anchor |
| 3 | Staphylococcus chromogenes | YSIRK_signal~rne~Rib~hyperosmo_Ebh~DUF4175~Gram_pos_anchor |
| 3 | Streptococcus suis | YSIRK_signal~PRK14949~He_PIG~Gram_pos_anchor |
| 3 | Streptococcus oralis | YSIRK_signal~MttA_Hcf106~Chb~G5~Gram_pos_anchor |
| 3 | Streptococcus | YSIRK_signal~Aim21~Chb~G5~Gram_pos_anchor |
| 3 | Streptococcus suis | YSIRK_signal~PTZ00121~GA~FIVAR~rne~hyperosmo_Ebh~rne~hyperosmo_Ebh~Gram_pos_anchor |
| 3 | Streptococcus parasanguinis | YSIRK_signal~MSCRAMM_SdrC~Peptidases_S8_S53~PA~fn3_5~FLgD_tudor~FlgD_ig~PRK11907~Gram_pos_anchor |
| 3 | Streptococcus | YSIRK_signal~MSCRAMM_SdrC~GH18_chitinase-like~F5_F8_type_C~Big_3~SbcC~FIVAR~G5~Gram_pos_anchor |
| 3 | Streptococcus | YSIRK_signal~rne~F5_F8_type_C~Glyco_hydro_20b~GH20_hexosaminidase~F5_F8_type_C~Glyco_hydro_20b~NAGidase~F5_F8_type_C~Gram_pos_anchor |
| 3 | Streptococcus | YSIRK_signal~LamG~Sialidase~MARCKS~Gram_pos_anchor |
| 3 | Lactobacillus salivarius | YSIRK_signal~COG4932~MucBP~Gram_pos_anchor |
| 3 | Lactobacillus acidophilus | YSIRK_signal~FIVAR~YhgE~DUF1542~PTZ00121~Gram_pos_anchor |
| 3 | Streptococcus agalactiae | YSIRK_signal~PTZ00121~DedD~Gram_pos_anchor |
| 3 | Streptococcus agalactiae | YSIRK_signal~PTZ00121~SucB_Actino~Trypan_PARP~Gram_pos_anchor |
| 3 | Streptococcus gallolyticus | YSIRK_signal~MSCRAMM_SdrC~pullulan_Gpos~AmyAc_family~pullulan_Gpos~Gram_pos_anchor |
| 3 | Streptococcus agalactiae | YSIRK_signal~MucBP~PRK10819~Gram_pos_anchor |
| 3 | Streptococcus parasanguinis | YSIRK_signal~MSCRAMM_SdrC~PRK15313~Gram_pos_anchor |
| 3 | Streptococcus | YSIRK_signal~BUD22~Rib~Gram_pos_anchor |
| 3 | Streptococcus mitis | YSIRK_signal~TonB_N~PRK11907~Gram_pos_anchor |
| 3 | Streptococcus | YSIRK_signal~MSCRAMM_SdrC~SbcC~GbpC~PTZ00449~Gram_pos_anchor |
| 3 | Lactobacillus johnsonii | YSIRK_signal~MSCRAMM_SdrD~DUF285~Rib~Gram_pos_anchor |
| 3 | Streptococcus suis | YSIRK_signal~MucBP~PHA03247~Gram_pos_anchor |
| 3 | Staphylococcus argenteus | YSIRK_signal~MSCRAMM_SdrC~SasC_Mrp_aggreg~DUF1542~SMC_prok_B~Gram_pos_anchor |
| 3 | Streptococcus oralis | YSIRK_signal~Tryp_SPc~AfuC~Calx-beta~ftsN~PHA03247~G5~Gram_pos_anchor |
| 3 | Streptococcus uberis | YSIRK_signal~PRK12678~Gram_pos_anchor |
| 3 | Staphylococcus aureus | YSIRK_signal~PTZ00449~AlphaC_N~Gram_pos_anchor |
| 3 | Streptococcus suis | YSIRK_signal~GH18_chitinase-like~F5_F8_type_C~Big_3~TBPIP~G5~Gram_pos_anchor |
| 3 | Lactobacillus mucosae | YSIRK_signal~Herpes_ICP4_C~MucBP~Gram_pos_anchor |
| 2 | Streptococcus sp. NPS 308 | YSIRK_signal~rne~He_PIG~CshA_fibril_rpt~repeat_SSSPR51~Gram_pos_anchor |
| 2 | Streptococcus suis | YSIRK_signal~PRK05035~MucBP~PTZ00441~repeat_SSSPR51~MucBP~repeat_SSSPR51~MucBP~repeat_SSSPR51~MucBP~repeat_SSSPR51~Gram_pos_anchor |
| 2 | Streptococcus agalactiae | YSIRK_signal~Neisseria_TspB~PRK10819~SucB_Actino~Gram_pos_anchor |
| 2 | Streptococcus mitis | YSIRK_signal~Trypan_PARP~F5_F8_type_C~Glyco_hydro_20b~GH20_hexosaminidase~F5_F8_type_C~Glyco_hydro_20b~NAGidase~Glyco_hydro_106~F5_F8_type_C~Gram_pos_anchor |
| 2 | Streptococcus suis | YSIRK_signal~PHA03247~MucBP~repeat_SSSPR51~Gram_pos_anchor |
| 2 | Macrococcus canis | YSIRK_signal~MSCRAMM_SdrC~MSCRAMM_SdrD~PTZ00449~Gram_pos_anchor |
| 2 | Lactobacillus mucosae | YSIRK_signal~PHA03247~MucBP~Gram_pos_anchor |
| 2 | Lactobacillus gasseri | YSIRK_signal~PRK14708~Gram_pos_anchor |
| 2 | Streptococcus suis | YSIRK_signal~GA~PTZ00121~FIVAR~hyperosmo_Ebh~Herpes_BLLF1~rne~hyperosmo_Ebh~rne~hyperosmo_Ebh~Gram_pos_anchor |
| 2 | Facklamia hominis | YSIRK_signal~G5~Flg_new~G5~Gram_pos_anchor |
| 2 | Streptococcus pneumoniae | YSIRK_signal~GAGBD~PTZ00449~Gram_pos_anchor |
| 2 | Lactobacillus oris | YSIRK_signal~PHA03255~PRK10856~Gram_pos_anchor |
| 2 | Streptococcus acidominimus | YSIRK_signal~He_PIG~Rib~Gram_pos_anchor |
| 2 | Streptococcus pneumoniae | YSIRK_signal~PTZ00121~RICH~Gram_pos_anchor |
| 2 | Staphylococcus pseudintermedius | YSIRK_signal~rne~termin_org_DnaJ~Rib~hyperosmo_Ebh~MSCRAMM_SdrC~Gram_pos_anchor |
| 2 | Streptococcus pneumoniae | YSIRK_signal~PHA03307~PTZ00121~Gram_pos_anchor |
| 2 | Streptococcus sp. oral taxon 071 | YSIRK_signal~Trypan_PARP~F5_F8_type_C~Glyco_hydro_20b~GH20_hexosaminidase~F5_F8_type_C~Glyco_hydro_20b~NAGidase~F5_F8_type_C~rne~Gram_pos_anchor |
| 2 | Staphylococcus haemolyticus | YSIRK_signal~MSCRAMM_SdrC~SasC_Mrp_aggreg~DUF1542~PTZ00121~DUF1542~Gram_pos_anchor |
| 2 | Lactobacillus | YSIRK_signal~MSCRAMM_SdrC~DUF5011~Gram_pos_anchor |
| 2 | Streptococcus suis | YSIRK_signal~GAGBD~PHA03247~rne~G5~Gram_pos_anchor |
| 2 | Streptococcus salivarius | YSIRK_signal~PTZ00121~GbpC~PHA02030~ProTailRpt~Gram_pos_anchor |
| 2 | Streptococcus oralis | YSIRK_signal~Tryp_SPc~AfuC~Calx-beta~PHA03247~G5~Gram_pos_anchor |
| 2 | Streptococcus oralis | YSIRK_signal~PRK05035~GH18_chitinase-like~F5_F8_type_C~Big_3~FIVAR~G5~Gram_pos_anchor |
| 2 | Streptococcus equi subsp. zooepidemicus | YSIRK_signal~Macoilin~SMC_prok_B~Gram_pos_anchor |
| 2 | Streptococcus oralis | YSIRK_signal~MSCRAMM_SdrC~Glyco_hyd_65N_2~ATH1~rne~Gram_pos_anchor |
| 2 | Streptococcus | YSIRK_signal~Tryp_SPc~AfuC~Calx-beta~SMC_prok_B~G5~Gram_pos_anchor |
| 2 | Streptococcus mitis | YSIRK_signal~RNase_Y~hyperosmo_Ebh~Gram_pos_anchor |
| 2 | Streptococcus oralis | YSIRK_signal~rne~Glyco_hyd_65N_2~ATH1~FN3~PRK13108~Gram_pos_anchor |
| 2 | Streptococcus | YSIRK_signal~PRK12495~CshA_fibril_rpt~Gram_pos_anchor |
| 2 | Streptococcus suis | YSIRK_signal~MSCRAMM_SdrC~Peptidases_S8_S53~PA~fn3_5~FlgD_ig~YicC_N~FIVAR~pullulan_Gpos~Gram_pos_anchor |
| 2 | Streptococcus suis | YSIRK_signal~PRK14949~Peptidase_M26_C~Gram_pos_anchor |
| 2 | Streptococcus suis | YSIRK_signal~PRK14960~Peptidases_S8_S53~PA~fn3_5~FlgD_ig~FIVAR~Gram_pos_anchor |
| 2 | Streptococcus suis | YSIRK_signal~MucBP~MISS~repeat_SSSPR51~MucBP~repeat_SSSPR51~MucBP~repeat_SSSPR51~MucBP~repeat_SSSPR51~Gram_pos_anchor |
| 2 | Facklamia hominis | YSIRK_signal~IsdB~Rib~Gram_pos_anchor |
| 2 | Bacilli | YSIRK_signal~PTZ00121~Rib~Gram_pos_anchor |
| 2 | Streptococcus equi | YSIRK_signal~Smc~SMC_prok_B~Gram_pos_anchor |
| 2 | Streptococcus agalactiae | YSIRK_signal~Neisseria_TspB~PRK10819~Gram_pos_anchor |
| 2 | Streptococcus | YSIRK_signal~DUF4775~Tryp_SPc~AfuC~Calx-beta~YabE~G5~Gram_pos_anchor |
| 2 | Streptococcus oralis | YSIRK_signal~DUF4775~Glyco_hyd_65N_2~ATH1~FN3~Gram_pos_anchor |
| 2 | Streptococcus oralis | YSIRK_signal~MSCRAMM_SdrC~AfuC~Calx-beta~PTZ00449~G5~Gram_pos_anchor |
| 2 | Streptococcus suis | YSIRK_signal~MSCRAMM_SdrC~Peptidases_S8_S53~PA~fn3_5~FlgD_ig~FIVAR~tolC~FIVAR~pullulan_Gpos~Gram_pos_anchor |
| 2 | Streptococcus suis | YSIRK_signal~MucBP~FAP~repeat_SSSPR51~Gram_pos_anchor |
| 2 | Facklamia hominis | YSIRK_signal~F1-ATPase_gamma~Gram_pos_anchor |
| 2 | Streptococcus suis | YSIRK_signal~PRK08026~PRK05035~MucBP~PTZ00441~repeat_SSSPR51~MucBP~repeat_SSSPR51~MucBP~repeat_SSSPR51~Gram_pos_anchor |
| 2 | Streptococcus azizii | YSIRK_signal~MARCKS~GH_101_like~Glyco_hyd_101C~F5_F8_type_C~G5~Gram_pos_anchor |
| 2 | Streptococcus suis | YSIRK_signal~CshA_fibril_rpt~PHA03247~MucBP~Gram_pos_anchor |
| 2 | Streptococcus suis | YSIRK_signal~rne~MucBP~PTZ00441~repeat_SSSPR51~MucBP~repeat_SSSPR51~Gram_pos_anchor |
| 2 | Streptococcus suis | YSIRK_signal~MucBP~PHA03247~MucBP~repeat_SSSPR51~Gram_pos_anchor |
| 2 | Streptococcus agalactiae | YSIRK_signal~PLN03237~Trypan_PARP~Gram_pos_anchor |
| 2 | Staphylococcus auricularis | YSIRK_signal~MDN1~YhgE~MARTX_Nterm~MDN1~PTZ00121~Gram_pos_anchor |
| 2 | Streptococcus | YSIRK_signal~MucBP~PRK15313~MucBP~repeat_SSSPR51~Gram_pos_anchor |
| 2 | Lactobacillus jensenii | YSIRK_signal~PTZ00121~FIVAR~Gram_pos_anchor |
| 2 | Streptococcus suis | YSIRK_signal~SMC_prok_B~GA~FIVAR~rne~hyperosmo_Ebh~rne~MSCRAMM_SdrC~hyperosmo_Ebh~termin_org_DnaJ~hyperosmo_Ebh~Gram_pos_anchor |
| 2 | Streptococcus sp. C150 | YSIRK_signal~PRK10856~COG4932~FctA~Gram_pos_anchor |
| 2 | Streptococcus suis | YSIRK_signal~PRK08026~MucBP~PRK15313~repeat_SSSPR51~Gram_pos_anchor |
| 2 | Streptococcus | YSIRK_signal~rne~Glyco_hyd_65N_2~ATH1~Gram_pos_anchor |
| 2 | Staphylococcus stepanovicii | YSIRK_signal~MSCRAMM_SdrC~MucBP~PTZ00441~MucBP~PTZ00441~MucBP~PTZ00441~MucBP~PTZ00441~MucBP~PTZ00441~MucBP~PTZ00441~MucBP~PTZ00441~MucBP~PTZ00441~MucBP~PTZ00441~Gram_pos_anchor |
| 2 | Streptococcus dysgalactiae | YSIRK_signal~vWFA~Glycosyltransferase_GTB-type~Fn_bind~Gram_pos_anchor |
| 2 | Lactobacillus iners | YSIRK_signal~PTZ00121~PRK05901~Gram_pos_anchor |
| 2 | Streptococcus suis | YSIRK_signal~SbcC~GA~FIVAR~hyperosmo_Ebh~rne~hyperosmo_Ebh~rne~MSCRAMM_SdrC~hyperosmo_Ebh~Gram_pos_anchor |
| 2 | Streptococcus suis | YSIRK_signal~VirB10~pullulan_Gpos~Gram_pos_anchor |
| 2 | Streptococcus | YSIRK_signal~F5_F8_type_C~Glyco_hydro_20b~GH20_hexosaminidase~F5_F8_type_C~Glyco_hydro_20b~NAGidase~F5_F8_type_C~Gram_pos_anchor |
| 2 | Streptococcus azizii | YSIRK_signal~Flg_new~Gram_pos_anchor |
| 2 | Streptococcus suis | YSIRK_signal~rne~MucBP~PHA03247~Rib~MSCRAMM_SdrC~pullulan_Gpos~Gram_pos_anchor |
| 2 | Streptococcus cristatus | YSIRK_signal~PRK00708~Chb~G5~Gram_pos_anchor |
| 2 | Staphylococcus auricularis | YSIRK_signal~PRK13108~MSCRAMM_SdrD~SdrG_C_C~Gram_pos_anchor |
| 2 | Staphylococcus chromogenes | YSIRK_signal~rne~Rib~Herpes_BLLF1~Gram_pos_anchor |
| 2 | Streptococcus | YSIRK_signal~MSCRAMM_SdrC~F5_F8_type_C~Glyco_hydro_20b~GH20_hexosaminidase~F5_F8_type_C~PTZ00449~Glyco_hydro_20b~NAGidase~F5_F8_type_C~rne~Gram_pos_anchor |
| 2 | Streptococcus | YSIRK_signal~MSCRAMM_SdrC~FctA~Gram_pos_anchor |
| 2 | Streptococcus parasanguinis | YSIRK_signal~PRK11907~FctA~Antigen_C~FctA~Antigen_C~FctA~Antigen_C~FctA~Antigen_C~FctA~Antigen_C~FctA~Antigen_C~FctA~Gram_pos_anchor |
| 2 | Staphylococcus pseudintermedius | YSIRK_signal~rne~termin_org_DnaJ~Rib~MSCRAMM_SdrC~Gram_pos_anchor |
| 2 | Streptococcus | YSIRK_signal~rne~GH18_chitinase-like~F5_F8_type_C~Big_3~FIVAR~HOOK~G5~Gram_pos_anchor |
| 2 | Lactobacillus iners | YSIRK_signal~SMC_N~DUF4988~He_PIG~Gram_pos_anchor |
| 2 | Streptococcus | YSIRK_signal~rne~vWFA~Gram_pos_anchor |
| 2 | Streptococcus henryi | YSIRK_signal~Endomucin~GH43_62_32_68_117_130~Gram_pos_anchor |
| 2 | Streptococcus mitis | YSIRK_signal~repeat_SSSPR51~Trypan_PARP~Gram_pos_anchor |
| 2 | Staphylococcus aureus | YSIRK_signal~PTZ00121~lectin_L-type~G5~SasG_E~G5~SasG_E~G5~SasG_E~G5~SasG_E~G5~SasG_E~G5~SasG_E~Gram_pos_anchor |
| 2 | Streptococcus suis | YSIRK_signal~MucBP~MISS~repeat_SSSPR51~MucBP~repeat_SSSPR51~MucBP~repeat_SSSPR51~Gram_pos_anchor |
| 2 | Streptococcus suis | YSIRK_signal~MucBP~MISS~repeat_SSSPR51~MucBP~repeat_SSSPR51~Gram_pos_anchor |
| 2 | Streptococcus mitis | YSIRK_signal~PHA03325~Smc~PTZ00449~DUF3824~PTZ00449~PHA03247~PTZ00449~repeat_SSSPR51~Gram_pos_anchor |
| 2 | Streptococcus suis | YSIRK_signal~MucBP~PTZ00441~repeat_SSSPR51~MucBP~repeat_SSSPR51~Gram_pos_anchor |
| 2 | Streptococcus | YSIRK_signal~Smc~GbpC~ProTailRpt~Gram_pos_anchor |
| 2 | Staphylococcus warneri | YSIRK_signal~PRK13108~MSCRAMM_SdrC~SdrG_C_C~Gram_pos_anchor |
| 2 | Streptococcus suis | YSIRK_signal~rne~MucBP~PTZ00441~repeat_SSSPR51~MucBP~repeat_SSSPR51~MucBP~repeat_SSSPR51~MucBP~repeat_SSSPR51~Gram_pos_anchor |
| 2 | Streptococcus | YSIRK_signal~PTZ00121~GbpC~Gram_pos_anchor |
| 2 | Streptococcus henryi | YSIRK_signal~pullulan_Gpos~AmyAc_family~pullulan_Gpos~PRK10856~Gram_pos_anchor |
| 2 | Streptococcus | YSIRK_signal~Trypan_PARP~GH18_chitinase-like~F5_F8_type_C~Big_3~FIVAR~G5~Gram_pos_anchor |
| 2 | Streptococcus suis | YSIRK_signal~PRK08026~MucBP~PHA03247~repeat_SSSPR51~Gram_pos_anchor |
| 2 | Streptococcus oralis | YSIRK_signal~MSCRAMM_SdrC~Glyco_hyd_65N_2~ATH1~CBP_CCPA~Gram_pos_anchor |
| 2 | Streptococcus | YSIRK_signal~DUF1542~PRK14949~DUF1542~Gram_pos_anchor |
| 2 | Lactobacillus hayakitensis | YSIRK_signal~PHA03378~Gram_pos_anchor |
| 2 | Lactobacillus oris | YSIRK_signal~MSCRAMM_SdrC~pullulan_Gpos~Gram_pos_anchor |
| 2 | Streptococcus | YSIRK_signal~He_PIG~LbR-like~RPT_S_cricet~PTZ00449~Gram_pos_anchor |
| 2 | Streptococcus suis | YSIRK_signal~PRK14951~vWFA~Gram_pos_anchor |
| 2 | Staphylococcus capitis | YSIRK_signal~NEAT~NEAT~PTZ00108~Gram_pos_anchor |
| 2 | Streptococcus | YSIRK_signal~Smc~DUF1542~Gram_pos_anchor |
| 2 | Streptococcus | YSIRK_signal~PTZ00121~Trypan_PARP~PRK10819~Gram_pos_anchor |
| 2 | Streptococcus pneumoniae | YSIRK_signal~GAGBD~PTZ00121~Gram_pos_anchor |
| 2 | Streptococcus pneumoniae | YSIRK_signal~GAGBD~SMC_prok_A~PTZ00449~Gram_pos_anchor |
| 2 | Streptococcus pneumoniae | YSIRK_signal~PTZ00449~Trypan_PARP~Gram_pos_anchor |
| 2 | Streptococcus pneumoniae | YSIRK_signal~Sialidase~Gram_pos_anchor |
| 2 | Streptococcus pneumoniae | YSIRK_signal~MSCRAMM_SdrC~GH18_chitinase-like~F5_F8_type_C~Big_3~G5~Gram_pos_anchor |
| 2 | Lactobacillus jensenii | YSIRK_signal~Flg_new~GA~PTZ00121~Gram_pos_anchor |
| 2 | Gemella | YSIRK_signal~Smc~PHA03247~repeat_SSSPR51~Gram_pos_anchor |
| 2 | Streptococcus suis | YSIRK_signal~DUF1542~hyperosmo_Ebh~Gram_pos_anchor |
| 2 | Streptococcus oralis | YSIRK_signal~BASP1~GH18_chitinase-like~F5_F8_type_C~Big_3~FIVAR~G5~Gram_pos_anchor |
| 2 | Lactobacillus reuteri | YSIRK_signal~MSCRAMM_SdrC~choice_anch_A~Gram_pos_anchor |
| 2 | Granulicatella | YSIRK_signal~MSCRAMM_SdrC~Glyco_hydro_20b~NAGidase~PRK10100~F5_F8_type_C~Gram_pos_anchor |
| 2 | Lactobacillus saerimneri | YSIRK_signal~SPR1~Gram_pos_anchor |
| 2 | Lactobacillus johnsonii | YSIRK_signal~Hia~PRK08581~Gram_pos_anchor |
| 2 | Gemella morbillorum | YSIRK_signal~MSCRAMM_SdrD~Abhydrolase~Rib~Gram_pos_anchor |
| 1 | Streptococcus oralis | YSIRK_signal~LamG~Sialidase~PHA03169~Gram_pos_anchor |
| 1 | Lactobacillus coleohominis | YSIRK_signal~SasC_Mrp_aggreg~Rib~Gram_pos_anchor |
| 1 | Salipaludibacillus aurantiacus | YSIRK_signal~SdrG_C_C~Gram_pos_anchor |
| 1 | Lactobacillus iners | YSIRK_signal~hyperosmo_Ebh~Gram_pos_anchor |
| 1 | Enterococcus columbae | YSIRK_signal~Abhydrolase~Gram_pos_anchor |
