# Supplementary material for: YSIRK-G/S-directed translocation is required for Streptococcus suis to deliver diverse cell wall anchoring effectors contributing to bacterial pathogenicity
Source: Virulence. 2020 Nov 2;11(1):1539–56. doi: 10.1080/21505594.2020.1838740 (PMC7644249; doi:10.1080/21505594.2020.1838740)
Supplement: Supplemental Material [file KVIR_A_1838740_SM7169.zip › Supplementary Table S4.docx]

| **Table S4** Proteins with a YSIRK or LPxTG motif not in N-terminus and C-terminus screened by domain-architecture retrieval. | | |
| --- | --- | --- |
| Sequences | Taxonomy | Domain Names |
| 461 | Streptococcus pneumoniae | YSIRK_signal~Gram_pos_anchor~YabE~G5~DUF4775~Peptidase_M26_N~Peptidase_M26_C |
| 305 | Streptococcus | YSIRK_signal~Gram_pos_anchor~YabE~G5~rne~Peptidase_M26_N~Peptidase_M26_C |
| 257 | Bacilli | YSIRK_signal~Gram_pos_anchor~G5~Peptidase_M26_N~Peptidase_M26_C |
| 189 | Streptococcus pneumoniae | YSIRK_signal~Gram_pos_anchor~YabE~G5~TonB_N~PTZ00441~Peptidase_M26_N~Peptidase_M26_C |
| 152 | Streptococcus | YSIRK_signal~Gram_pos_anchor~YabE~G5~rne~Tryp_SPc~Peptidase_M26_N~Peptidase_M26_C |
| 144 | Streptococcus | YSIRK_signal~Gram_pos_anchor~YabE~G5~TonB_N~Peptidase_M26_N~Peptidase_M26_C |
| 101 | Streptococcus pneumoniae | YSIRK_signal~Gram_pos_anchor~YabE~G5~TonB_N~MSA_2~Peptidase_M26_N~Glug~Peptidase_M26_C |
| 86 | Streptococcus pneumoniae | YSIRK_signal~Gram_pos_anchor~YabE~G5~TonB_N~PRK08691~Peptidase_M26_N~Peptidase_M26_C |
| 86 | Streptococcus pneumoniae | YSIRK_signal~Gram_pos_anchor~YabE~G5~TonB_N~Peptidase_M26_N~Glug~Peptidase_M26_C |
| 84 | Streptococcus | YSIRK_signal~Gram_pos_anchor~rne~G5~Peptidase_M26_N~PRK12758~Peptidase_M26_C |
| 75 | Streptococcus | YSIRK_signal~Gram_pos_anchor~YabE~G5~rne~Peptidase_M26_N~Glug~Peptidase_M26_C |
| 75 | Streptococcus pneumoniae | YSIRK_signal~Gram_pos_anchor~YabE~G5~TonB_N~MSA_2~Peptidase_M26_N~Peptidase_M26_C |
| 71 | Streptococcus | YSIRK_signal~Gram_pos_anchor~rne~G5~Peptidase_M26_N~Peptidase_M26_C |
| 65 | Streptococcus | YSIRK_signal~Gram_pos_anchor~YabE~G5~PRK10263~Peptidase_M26_N~Peptidase_M26_C |
| 64 | Streptococcus | YSIRK_signal~Gram_pos_anchor~YabE~G5~PRK10263~Peptidase_M26_N~Glug~Peptidase_M26_C |
| 64 | Streptococcus pneumoniae | YSIRK_signal~Gram_pos_anchor~YabE~G5~SucB_Actino~Tryp_SPc~Peptidase_M26_N~Peptidase_M26_C |
| 59 | Streptococcus pneumoniae | YSIRK_signal~Gram_pos_anchor~TonB_N~G5~Peptidase_M26_N~Peptidase_M26_C |
| 52 | Streptococcus pneumoniae | YSIRK_signal~Gram_pos_anchor~YabE~G5~TonB_N~Treacle~Peptidase_M26_N~Peptidase_M26_C |
| 52 | Streptococcus pneumoniae | YSIRK_signal~Gram_pos_anchor~YabE~G5~TonB_N~2A1904~Peptidase_M26_N~Peptidase_M26_C |
| 45 | Streptococcus | YSIRK_signal~Gram_pos_anchor~PHA03247~G5~Peptidase_M26_N~Peptidase_M26_C |
| 45 | Streptococcus pneumoniae | YSIRK_signal~Gram_pos_anchor~TonB_N~rne~G5~Peptidase_M26_N~Peptidase_M26_C |
| 42 | Streptococcus pneumoniae | YSIRK_signal~Gram_pos_anchor~YabE~G5~DUF4775~Peptidase_M26_N~Glug~Peptidase_M26_C |
| 41 | Streptococcus pneumoniae | YSIRK_signal~Gram_pos_anchor~YabE~G5~DUF4775~Metaviral_G~Peptidase_M26_N~Peptidase_M26_C |
| 35 | Streptococcus | YSIRK_signal~Gram_pos_anchor~G5~rne~Peptidase_M26_N~Peptidase_M26_C |
| 32 | Streptococcus pneumoniae | YSIRK_signal~Gram_pos_anchor~YabE~G5~BASP1~Peptidase_M26_N~Peptidase_M26_C |
| 30 | Streptococcus | YSIRK_signal~Gram_pos_anchor~PHA03169~G5~Peptidase_M26_N~Peptidase_M26_C |
| 28 | Streptococcus | YSIRK_signal~Gram_pos_anchor~G5~PRK10263~Peptidase_M26_N~Glug~Peptidase_M26_C |
| 28 | Streptococcus pneumoniae | YSIRK_signal~Gram_pos_anchor~racA~G5~Herpes_BLLF1~Peptidase_M26_N~Peptidase_M26_C |
| 26 | Streptococcus pneumoniae | YSIRK_signal~Gram_pos_anchor~YabE~G5~TonB_N~PHA03273~Peptidase_M26_N~Peptidase_M26_C |
| 23 | Streptococcus | YSIRK_signal~Gram_pos_anchor~G5~PRK10263~Peptidase_M26_N~Peptidase_M26_C |
| 21 | Streptococcus | YSIRK_signal~Gram_pos_anchor~G5~rne~Peptidase_M26_N~Glug~Peptidase_M26_C |
| 21 | Streptococcus pneumoniae | YSIRK_signal~Gram_pos_anchor~Aim21~Peptidase_M26_N~Glug~Peptidase_M26_C |
| 20 | Streptococcus suis | YSIRK_signal~Gram_pos_anchor~G5~Peptidase_M26_N~PRK14708~Peptidase_M26_C |
| 20 | Streptococcus pneumoniae | YSIRK_signal~Gram_pos_anchor~YabE~G5~TonB_N~PRK08691~Peptidase_M26_N~Glug~Peptidase_M26_C |
| 19 | Streptococcus pneumoniae | YSIRK_signal~Gram_pos_anchor~YabE~G5~TonB_N~Mycoplas_LppA~Peptidase_M26_N~Glug~Peptidase_M26_C |
| 16 | Streptococcus pneumoniae | YSIRK_signal~Gram_pos_anchor~rne~G5~Peptidase_M26_N~Glug~Peptidase_M26_C |
| 16 | Streptococcus pneumoniae | YSIRK_signal~Gram_pos_anchor~YabE~G5~TonB_N~PRK08581~Peptidase_M26_N~Peptidase_M26_C |
| 15 | Streptococcus | YSIRK_signal~Gram_pos_anchor~G5~PHA03247~G5~YabE~G5~YabE~G5~Peptidase_M26_C |
| 14 | Streptococcus pneumoniae | YSIRK_signal~Gram_pos_anchor~YabE~G5~Herpes_BLLF1~Peptidase_M26_N~Peptidase_M26_C |
| 14 | Streptococcus pneumoniae | YSIRK_signal~Gram_pos_anchor~G5~PRK10263~G5~Peptidase_M26_C |
| 13 | Streptococcus pneumoniae | YSIRK_signal~Gram_pos_anchor~YabE~G5~rne~Tryp_SPc~Peptidase_M26_N |
| 12 | Streptococcus pneumoniae | YSIRK_signal~Gram_pos_anchor~racA~G5~TonB_N~PTZ00441~Peptidase_M26_N~Peptidase_M26_C |
| 11 | Streptococcus pneumoniae | YSIRK_signal~Gram_pos_anchor~YabE~G5~SucB_Actino~Peptidase_M26_N~Peptidase_M26_C |
| 11 | Streptococcus pneumoniae | YSIRK_signal~Gram_pos_anchor~YabE~rne~G5~Tryp_SPc~Peptidase_M26_N~Peptidase_M26_C |
| 11 | Streptococcus | YSIRK_signal~Gram_pos_anchor~rne~Peptidase_M26_N~Peptidase_M26_C |
| 10 | Streptococcus pneumoniae | YSIRK_signal~Gram_pos_anchor~PHA03169~Peptidase_M26_N~Glug~Peptidase_M26_C |
| 10 | Streptococcus | YSIRK_signal~Gram_pos_anchor~G5~Peptidase_M26_C |
| 10 | Streptococcus pneumoniae | YSIRK_signal~Gram_pos_anchor~YabE~G5~TonB_N~Mycoplas_LppA~Peptidase_M26_N~Peptidase_M26_C |
| 10 | Streptococcus pneumoniae | YSIRK_signal~Gram_pos_anchor~YabE~G5~Peptidase_M26_N~Glug~Peptidase_M26_C |
| 9 | Streptococcus pneumoniae | YSIRK_signal~Gram_pos_anchor~racA~G5~TonB_N~Peptidase_M26_N~Peptidase_M26_C |
| 9 | Streptococcus | YSIRK_signal~Gram_pos_anchor~PHA03247~Peptidase_M26_N~Peptidase_M26_C |
| 8 | Streptococcus pneumoniae | YSIRK_signal~Gram_pos_anchor~YabE~G5~PRK10819~PTZ00441~Peptidase_M26_N |
| 8 | Streptococcus pneumoniae | YSIRK_signal~Gram_pos_anchor~YabE~G5~BASP1~PRK09418~Peptidase_M26_N~Glug~Peptidase_M26_C |
| 7 | Streptococcus pneumoniae | YSIRK_signal~Gram_pos_anchor~YabE~G5~PRK10819~Peptidase_M26_N~Peptidase_M26_C |
| 7 | Streptococcus pneumoniae | YSIRK_signal~Gram_pos_anchor~G5~PRK10263~G5~YabE~G5~Peptidase_M26_C |
| 7 | Streptococcus pneumoniae | YSIRK_signal~FimV~Gram_pos_anchor~NESP55~G5~Peptidase_M26_N |
| 7 | Streptococcus pneumoniae | YSIRK_signal~Gram_pos_anchor~G5~2A1904~Peptidase_M26_N |
| 7 | Streptococcus | YSIRK_signal~Gram_pos_anchor~PTZ00121~Peptidase_M26_N~Glug~Peptidase_M26_C |
| 6 | Streptococcus pneumoniae | YSIRK_signal~Gram_pos_anchor~YabE~G5~2A1904~Peptidase_M26_N~Peptidase_M26_C |
| 6 | Streptococcus pneumoniae | YSIRK_signal~Gram_pos_anchor~YabE~G5~2A1904~Peptidase_M26_N |
| 6 | Streptococcus pneumoniae | YSIRK_signal~Gram_pos_anchor~G5~MARCKS~PRK03427~G5~YabE~Peptidase_M26_C |
| 6 | Streptococcus pneumoniae | YSIRK_signal~Gram_pos_anchor~YabE~G5~rne~Peptidase_M26_N |
| 6 | Streptococcus pneumoniae | YSIRK_signal~Gram_pos_anchor~YabE~G5~TonB_N~termin_org_DnaJ~Peptidase_M26_N~Peptidase_M26_C |
| 6 | Streptococcus pneumoniae | YSIRK_signal~Gram_pos_anchor~YabE~G5~TonB_N~PTZ00441~Peptidase_M26_N~Glug~Peptidase_M26_C |
| 6 | Streptococcus | YSIRK_signal~Gram_pos_anchor~G5~PRK10263~G5~YabE~Peptidase_M26_C |
| 6 | Streptococcus pneumoniae | YSIRK_signal~Gram_pos_anchor~YabE~G5~PRK13108~Peptidase_M26_N~Peptidase_M26_C |
| 6 | Streptococcus | YSIRK_signal~Gram_pos_anchor~G5~Peptidase_M26_N |
| 5 | Streptococcus | YSIRK_signal~Gram_pos_anchor~BASP1~G5~Peptidase_M26_N~Peptidase_M26_C |
| 5 | Streptococcus | YSIRK_signal~Gram_pos_anchor~G5~PHA03379~Peptidase_M26_N~Peptidase_M26_C |
| 5 | Streptococcus pneumoniae | YSIRK_signal~Gram_pos_anchor~YabE~G5~PRK10263~Peptidase_M26_N~Glug |
| 5 | Streptococcus pneumoniae | YSIRK_signal~Gram_pos_anchor~Aim21~Peptidase_M26_N~Peptidase_M26_C |
| 5 | Streptococcus pneumoniae | YSIRK_signal~Gram_pos_anchor~rne~G5~Tryp_SPc~Peptidase_M26_N~Peptidase_M26_C |
| 5 | Streptococcus pneumoniae | YSIRK_signal~Gram_pos_anchor~YabE~G5~DUF4775~TonB_N~Peptidase_M26_N~Peptidase_M26_C |
| 5 | Streptococcus pneumoniae | YSIRK_signal~Gram_pos_anchor~YabE~G5~PRK10263~TonB_N~Peptidase_M26_N~Peptidase_M26_C |
| 4 | Streptococcus | YSIRK_signal~MSCRAMM_SdrD~Gram_pos_anchor~G5~Peptidase_M26_C |
| 4 | Streptococcus pneumoniae | YSIRK_signal~Gram_pos_anchor~YabE~G5~PRK10263~Peptidase_M26_N |
| 4 | Streptococcus suis | YSIRK_signal~Gram_pos_anchor~G5~Peptidase_M26_N~PRK12806~Peptidase_M26_C |
| 4 | Streptococcus pneumoniae | YSIRK_signal~Gram_pos_anchor~TonB_N~rne~G5~Peptidase_M26_N |
| 4 | Streptococcus pneumoniae | YSIRK_signal~Gram_pos_anchor~DUF4775~G5~Peptidase_M26_C |
| 4 | Streptococcus | YSIRK_signal~Gram_pos_anchor~G5~PTZ00121~PHA03247~G5~Peptidase_M26_C |
| 4 | Streptococcus | YSIRK_signal~Gram_pos_anchor~PHA03379~G5~Peptidase_M26_N~Peptidase_M26_C |
| 3 | Streptococcus pneumoniae | YSIRK_signal~Gram_pos_anchor~YabE~G5~rne~Peptidase_M26_N~Glug |
| 3 | Streptococcus pneumoniae | YSIRK_signal~Gram_pos_anchor~MDN1~G5~Peptidase_M26_N |
| 3 | Streptococcus pneumoniae | YSIRK_signal~Gram_pos_anchor~YabE~G5~PRK10819~PTZ00441~Peptidase_M26_N~Peptidase_M26_C |
| 3 | Streptococcus pneumoniae | YSIRK_signal~Gram_pos_anchor~PTZ00121~G5~Peptidase_M26_N |
| 3 | Streptococcus pneumoniae | YSIRK_signal~Gram_pos_anchor~PTZ00121~Peptidase_M26_N |
| 3 | Streptococcus pneumoniae | YSIRK_signal~Gram_pos_anchor~YabE~G5~DedD~TonB_N~Peptidase_M26_N~Glug~Peptidase_M26_C |
| 3 | Streptococcus pneumoniae | YSIRK_signal~Gram_pos_anchor~termin_org_DnaJ~TonB_N~G5~Peptidase_M26_N~Peptidase_M26_C |
| 3 | Streptococcus pneumoniae | YSIRK_signal~Gram_pos_anchor~YabE~G5~PRK10819~MSA_2~Peptidase_M26_N~Peptidase_M26_C |
| 3 | Streptococcus pneumoniae | YSIRK_signal~Gram_pos_anchor~YabE~G5~rne~PRK08691~Peptidase_M26_N |
| 3 | Streptococcus pneumoniae | YSIRK_signal~Gram_pos_anchor~Aim21~Peptidase_M26_N |
| 3 | Streptococcus pneumoniae | YSIRK_signal~Gram_pos_anchor~PHA03169~G5~Peptidase_M26_N |
| 2 | Streptococcus pneumoniae | YSIRK_signal~Gram_pos_anchor~YabE~G5~PRK10263~G5~Peptidase_M26_N~PRK12758~Peptidase_M26_C |
| 2 | Streptococcus pneumoniae | YSIRK_signal~Gram_pos_anchor~YabE~G5~BASP1~PRK09418~Peptidase_M26_N~Peptidase_M26_C |
| 2 | Streptococcus pneumoniae | YSIRK_signal~Gram_pos_anchor~YabE~G5~PRK10819~termin_org_DnaJ~Peptidase_M26_N~Peptidase_M26_C |
| 2 | Streptococcus pneumoniae | YSIRK_signal~Gram_pos_anchor~TonB_N~G5~Peptidase_M26_N~PRK12758~Peptidase_M26_C |
| 2 | Streptococcus pneumoniae | YSIRK_signal~Gram_pos_anchor~YabE~G5~PRK10819~2A1904~Peptidase_M26_N~Peptidase_M26_C |
| 2 | Streptococcus | YSIRK_signal~Gram_pos_anchor~rne~Peptidase_M26_N~Glug~Peptidase_M26_C |
| 2 | Streptococcus oralis | YSIRK_signal~Gram_pos_anchor~YabE~PHA03379~G5~Peptidase_M26_N~Peptidase_M26_C |
| 2 | Streptococcus | YSIRK_signal~Gram_pos_anchor~G5~PHA03169~G5~Peptidase_M26_C |
| 2 | Streptococcus pneumoniae | YSIRK_signal~Gram_pos_anchor~Peptidase_M26_C |
| 2 | Streptococcus pneumoniae | YSIRK_signal~Gram_pos_anchor~G5~PHA03247~PTZ00449~Peptidase_M26_C |
| 2 | Bacilli | YSIRK_signal~Gram_pos_anchor~G5~FIVAR~Peptidase_M26_N~Peptidase_M26_C |
| 2 | Streptococcus pneumoniae | YSIRK_signal~Gram_pos_anchor~YabE~G5~DUF612~Peptidase_M26_N~Peptidase_M26_C |
| 2 | Streptococcus pneumoniae | YSIRK_signal~Gram_pos_anchor~YabE~G5~rne~PTZ00441~Peptidase_M26_N |
| 2 | Streptococcus pneumoniae | YSIRK_signal~Gram_pos_anchor~G5~PHA03247~PTZ00121~G5~Peptidase_M26_C |
| 2 | Streptococcus pneumoniae | YSIRK_signal~Gram_pos_anchor~PHA03169~Peptidase_M26_N~Peptidase_M26_C |
| 2 | Streptococcus suis | YSIRK_signal~Gram_pos_anchor~G5~Peptidase_M26_N~Nudix_Hydrolase |
| 2 | Terrabacteria group | YSIRK_signal~Gram_pos_anchor~MSA_2~G5~Peptidase_M26_N~Peptidase_M26_C |
| 2 | Streptococcus mitis | YSIRK_signal~Gram_pos_anchor~YabE~G5~PHA03247~Peptidase_M26_N~Glug~Peptidase_M26_C |
| 1 | Streptococcus pneumoniae | YSIRK_signal~Gram_pos_anchor~Peptidase_M26_N~Glug~Peptidase_M26_C |
| 71 | Streptococcus | YSIRK_signal~Gram_pos_anchor~YabE~G5 |
| 36 | Streptococcus suis | YSIRK_signal~Gram_pos_anchor~PTZ00449~G5~CAP |
| 24 | Streptococcus pneumoniae | YSIRK_signal~Gram_pos_anchor~YabE~G5~rne |
| 24 | Lactobacillales | YSIRK_signal~Gram_pos_anchor~G5 |
| 23 | Streptococcus suis | YSIRK_signal~Gram_pos_anchor~PRK04335~G5~CAP |
| 17 | Streptococcus | YSIRK_signal~Gram_pos_anchor~rne~G5 |
| 16 | Streptococcus | YSIRK_signal~Gram_pos_anchor~rne |
| 9 | Streptococcus pneumoniae | YSIRK_signal~Gram_pos_anchor~G5~PRK10819 |
| 9 | Streptococcus suis | YSIRK_signal~Gram_pos_anchor~PTZ00449~G5~YkwD |
| 8 | Streptococcus pneumoniae | YSIRK_signal~Gram_pos_anchor~G5~2A1904 |
| 7 | Streptococcus pneumoniae | YSIRK_signal~Gram_pos_anchor~YabE~G5~PRK10819 |
| 6 | Streptococcus pneumoniae | YSIRK_signal~Gram_pos_anchor~G5~rne |
| 5 | Streptococcus pneumoniae | YSIRK_signal~Gram_pos_anchor~TonB_N |
| 5 | Streptococcus | YSIRK_signal~Gram_pos_anchor~YabE~G5~rne~Tryp_SPc |
| 5 | Streptococcus pneumoniae | YSIRK_signal~Gram_pos_anchor~PTZ00121 |
| 4 | Streptococcus suis | YSIRK_signal~Gram_pos_anchor~PRK13108~G5~CAP |
| 4 | Streptococcus pneumoniae | YSIRK_signal~MSCRAMM_SdrC~Gram_pos_anchor~G5 |
| 4 | Streptococcus pneumoniae | YSIRK_signal~Gram_pos_anchor~G5~PHA03247~G5 |
| 3 | Streptococcus pneumoniae | YSIRK_signal~MSCRAMM_SdrD~Gram_pos_anchor~G5 |
| 3 | Streptococcus suis | YSIRK_signal~Gram_pos_anchor~DUF4775~G5~YkwD |
| 2 | Streptococcus pseudopneumoniae | YSIRK_signal~Gram_pos_anchor~PRK13108 |
| 2 | Streptococcus pneumoniae | YSIRK_signal~Gram_pos_anchor~G5~PRK10263 |
| 2 | Streptococcus pneumoniae | YSIRK_signal~Gram_pos_anchor~PHA03169~G5 |
| 2 | Streptococcus suis | YSIRK_signal~Gram_pos_anchor~TonB~G5~CAP |
| 2 | Streptococcus pseudopneumoniae | YSIRK_signal~Gram_pos_anchor~G5~PRK13108 |
| 2 | Streptococcus pneumoniae | YSIRK_signal~Gram_pos_anchor~PTZ00121~rne |
|  |  |  |
| 92 | Streptococcus | MSCRAMM_SdrC~YSIRK_signal~Chb~G5~Gram_pos_anchor |
| 81 | Streptococcus pneumoniae | IsdB~YSIRK_signal~LacZ~Big_4~YabE~G5~Gram_pos_anchor |
| 50 | Streptococcus | MSCRAMM_SdrC~YSIRK_signal~CshA_fibril_rpt~Gram_pos_anchor |
| 35 | Bacilli | MSCRAMM_SdrC~YSIRK_signal~Gram_pos_anchor |
| 27 | Streptococcus | IsdB~YSIRK_signal~COG4932~FctA~Gram_pos_anchor |
| 21 | Terrabacteria group | MSCRAMM_SdrC~YSIRK_signal~CshA_fibril_rpt~repeat_SSSPR51~Gram_pos_anchor |
| 21 | Streptococcus | MSCRAMM_SdrC~YSIRK_signal~pullulan_Gpos~AmyAc_family~pullulan_Gpos~Gram_pos_anchor |
| 20 | Streptococcus | MSCRAMM_SdrC~YSIRK_signal~LacZ~Big_4~G5~Gram_pos_anchor |
| 16 | Lactobacillus | MSCRAMM_SdrC~YSIRK_signal~PHA03247~Gram_pos_anchor |
| 15 | Streptococcus | MSCRAMM_SdrC~YSIRK_signal~LamG~Sialidase~Gram_pos_anchor |
| 14 | Lactobacillales | MSCRAMM_SdrC~YSIRK_signal~Rib~Gram_pos_anchor |
| 13 | Streptococcus | MSCRAMM_SdrC~YSIRK_signal~hyperosmo_Ebh~Rib~He_PIG~Rib~Gram_pos_anchor |
| 13 | Staphylococcus aureus | MSCRAMM_SdrC~YSIRK_signal~AlphaC_N~Gram_pos_anchor |
| 13 | Bacilli | MSCRAMM_SdrC~YSIRK_signal~PTZ00449~Gram_pos_anchor |
| 13 | Bacilli | MSCRAMM_SdrC~YSIRK_signal~hyperosmo_Ebh~Rib~Gram_pos_anchor |
| 11 | Staphylococcus | MSCRAMM_SdrC~YSIRK_signal~PTZ00121~Gram_pos_anchor |
| 10 | Streptococcus | MSCRAMM_SdrC~YSIRK_signal~G5~Collagen~RPT_S_cricet~PTZ00449~Gram_pos_anchor |
| 10 | Streptococcus | IsdB~YSIRK_signal~Glyco_hydro_20b~NAGidase~F5_F8_type_C~Gram_pos_anchor |
| 8 | Streptococcus | MSCRAMM_SdrC~YSIRK_signal~Peptidases_S8_S53~PA~fn3_5~FlgD_ig~PRK11907~Gram_pos_anchor |
| 8 | Lactobacillus gasseri | MSCRAMM_SdrD~YSIRK_signal~Rib~Gram_pos_anchor |
| 7 | Streptococcus agalactiae | MSCRAMM_SdrC~YSIRK_signal~He_PIG~Rib~Gram_pos_anchor |
| 7 | Streptococcus | IsdH_HarA~YSIRK_signal~Glyco_hyd_65N_2~ATH1~FN3~Gram_pos_anchor |
| 6 | Streptococcus cristatus | IsdB~YSIRK_signal~LacZ~Big_4~G5~Gram_pos_anchor |
| 6 | Staphylococcus lugdunensis | MSCRAMM_SdrC~YSIRK_signal~hyperosmo_Ebh~Gram_pos_anchor |
| 5 | Staphylococcus | MSCRAMM_SdrC~YSIRK_signal~COG5651~G5~DUF2460~G5~PHA03247~Gram_pos_anchor |
| 5 | Staphylococcus chromogenes | MSCRAMM_SdrC~YSIRK_signal~rne~Rib~PRK15316~Gram_pos_anchor |
| 5 | Lactobacillus reuteri | MSCRAMM_SdrC~YSIRK_signal~COG4251~PHA03247~Gram_pos_anchor |
| 4 | Staphylococcus sciuri | MSCRAMM_SdrC~YSIRK_signal~NEAT~IsdB~NEAT~NEAT~Gram_pos_anchor |
| 4 | Staphylococcus | MSCRAMM_SdrC~YSIRK_signal~NEAT~IsdH_HarA~NEAT~TonB~Gram_pos_anchor |
| 4 | Staphylococcus sciuri | MSCRAMM_SdrC~YSIRK_signal~NEAT~IsdH_HarA~NEAT~NEAT~Gram_pos_anchor |
| 4 | Staphylococcus | MSCRAMM_SdrC~YSIRK_signal~SasC_Mrp_aggreg~DUF1542~Smc~Gram_pos_anchor |
| 4 | Streptococcus | MSCRAMM_SdrC~YSIRK_signal~LacZ~Big_4~YabE~G5~Gram_pos_anchor |
| 4 | Streptococcus | MSCRAMM_SdrC~YSIRK_signal~FctA~Antigen_C~FctA~Antigen_C~FctA~Antigen_C~FctA~Antigen_C~FctA~Antigen_C~FctA~Gram_pos_anchor |
| 4 | Staphylococcus carnosus | MSCRAMM_SdrD~YSIRK_signal~PRK14949~PTZ00121~Gram_pos_anchor |
| 4 | Streptococcus mitis | MSCRAMM_SdrC~YSIRK_signal~PRK08026~Rib~repeat_SSSPR51~Gram_pos_anchor |
| 4 | Lactobacillus gasseri | MSCRAMM_SdrC~YSIRK_signal~PHA03369~MISS~Herpes_BLLF1~Gram_pos_anchor |
| 4 | Lactobacillus johnsonii | MSCRAMM_SdrC~YSIRK_signal~MucBP~APC_basic~Gram_pos_anchor |
| 3 | Streptococcus infantis | MSCRAMM_SdrC~YSIRK_signal~FctA~Antigen_C~FctA~Antigen_C~FctA~Antigen_C~FctA~Antigen_C~FctA~Gram_pos_anchor |
| 3 | Streptococcus | MSCRAMM_SdrC~YSIRK_signal~Peptidases_S8_S53~PA~fn3_5~FlgD_ig~FIVAR~pullulan_Gpos~Gram_pos_anchor |
| 3 | Lactobacillus | MSCRAMM_SdrC~YSIRK_signal~TonB~Gram_pos_anchor |
| 3 | Lactobacillales | MSCRAMM_SdrC~YSIRK_signal~MucBP~PTZ00449~Gram_pos_anchor |
| 3 | Lactobacillus gasseri | MSCRAMM_SdrC~YSIRK_signal~PHA03369~PTZ00449~MISS~Herpes_BLLF1~TonB_N~Gram_pos_anchor |
| 3 | Staphylococcus chromogenes | MSCRAMM_SdrC~YSIRK_signal~rne~Rib~Herpes_BLLF1~Gram_pos_anchor |
| 3 | Staphylococcus chromogenes | MSCRAMM_SdrC~YSIRK_signal~rne~Rib~Gram_pos_anchor |
| 3 | Streptococcus | PRK11907~YSIRK_signal~Glyco_hyd_65N_2~ATH1~FN3~Gram_pos_anchor |
| 3 | Lactobacillus | MSCRAMM_SdrC~YSIRK_signal~MucBP~Gram_pos_anchor |
| 3 | Streptococcus sobrinus | MSCRAMM_SdrC~YSIRK_signal~GbpC~PHA03247~Gram_pos_anchor |
| 3 | Streptococcus oralis | PTZ00121~YSIRK_signal~CshA_fibril_rpt~repeat_SSSPR51~Gram_pos_anchor |
| 3 | Streptococcus suis | MSCRAMM_SdrC~YSIRK_signal~MucBP~PRK15313~repeat_SSSPR51~Gram_pos_anchor |
| 3 | Streptococcus suis | MSCRAMM_SdrC~YSIRK_signal~PRK08026~Gram_pos_anchor |
| 2 | Lactobacillus gasseri | MSCRAMM_SdrC~YSIRK_signal~PHA03369~PTZ00449~MISS~Herpes_BLLF1~Gram_pos_anchor |
| 2 | Streptococcus suis | MSCRAMM_SdrC~YSIRK_signal~YfdX~MucBP~PRK15313~MucBP~repeat_SSSPR51~Gram_pos_anchor |
| 2 | Streptococcus oralis | PRK11907~YSIRK_signal~Glyco_hyd_65N_2~ATH1~FN3~PRK13108~Gram_pos_anchor |
| 2 | Streptococcus | PRK11907~YSIRK_signal~Glyco_hyd_65N_2~ATH1~rne~Gram_pos_anchor |
| 2 | Streptococcus | MSCRAMM_SdrC~YSIRK_signal~LacZ~Big_4~Gram_pos_anchor |
| 2 | Streptococcus mitis | MSCRAMM_SdrD~YSIRK_signal~LamG~Sialidase~PHA03169~Gram_pos_anchor |
| 2 | Streptococcus oralis | IsdB~YSIRK_signal~Glyco_hyd_65N_2~ATH1~FN3~PRK10263~Gram_pos_anchor |
| 2 | Streptococcus oralis | IsdH_HarA~YSIRK_signal~Glyco_hyd_65N_2~ATH1~FN3~rne~Gram_pos_anchor |
| 2 | Streptococcus | IsdB~YSIRK_signal~F5_F8_type_C~Glyco_hydro_20b~GH20_hexosaminidase~F5_F8_type_C~Glyco_hydro_20b~NAGidase~F5_F8_type_C~Gram_pos_anchor |
| 2 | Streptococcus oralis | IsdB~YSIRK_signal~CshA_fibril_rpt~repeat_SSSPR51~Gram_pos_anchor |
| 2 | Streptococcus agalactiae | MSCRAMM_SdrC~YSIRK_signal~hyperosmo_Ebh~Rib~He_PIG~Gram_pos_anchor |
| 2 | Streptococcus oralis | IsdH_HarA~YSIRK_signal~PRK03918~GAGBD~Pro-rich~Gram_pos_anchor |
| 2 | Streptococcus oralis | MSCRAMM_SdrD~YSIRK_signal~LamG~Sialidase~Gram_pos_anchor |
| 2 | Lactobacillus iners | MSCRAMM_SdrC~YSIRK_signal~Flg_new~MucBP~Gram_pos_anchor |
| 2 | Lactobacillus gasseri | MSCRAMM_SdrD~YSIRK_signal~Hia~PHA03369~PTZ00449~PRK00708~Gram_pos_anchor |
| 2 | Bacilli | MSCRAMM_SdrC~YSIRK_signal~BASP1~YncE~Gram_pos_anchor |
| 2 | Lactobacillus hamsteri | MSCRAMM_SdrD~YSIRK_signal~SMC_prok_A~GbpC~PRK03427~Gram_pos_anchor |
| 2 | Streptococcus suis | MSCRAMM_SdrC~YSIRK_signal~He_PIG~Gram_pos_anchor |
| 2 | Lactobacillus acidophilus | MSCRAMM_SdrD~YSIRK_signal~5~He_PIG~Rib~Gram_pos_anchor |
| 2 | Streptococcus suis | MSCRAMM_SdrC~YSIRK_signal~MucBP~PHA03247~MucBP~repeat_SSSPR51~Gram_pos_anchor |
| 2 | Lactobacillus johnsonii | MSCRAMM_SdrC~YSIRK_signal~COG5099~He_PIG~Rib~Gram_pos_anchor |
| 2 | Staphylococcus cohnii | MSCRAMM_SdrC~YSIRK_signal~PTZ00449~PTZ00441~Gram_pos_anchor |
| 2 | Staphylococcus sciuri | MSCRAMM_SdrC~YSIRK_signal~NEAT~IsdH_HarA~NEAT~pullulan_Gpos~Gram_pos_anchor |
| 2 | Lactobacillus acidophilus | MSCRAMM_SdrD~YSIRK_signal~MISS~PTZ00449~PHA03247~Gram_pos_anchor |
| 2 | Staphylococcus simulans | MSCRAMM_SdrD~YSIRK_signal~COG5651~G5~PHA03247~Gram_pos_anchor |
| 2 | Lactobacillus gasseri | MSCRAMM_SdrC~YSIRK_signal~Hia~PRK00708~Gram_pos_anchor |
| 2 | Terrabacteria group | MSCRAMM_SdrC~YSIRK_signal~YncE~Gram_pos_anchor |
| 2 | Staphylococcus hominis | MSCRAMM_SdrC~YSIRK_signal~Herpes_BLLF1~YncE~Gram_pos_anchor |
| 2 | Streptococcus pyogenes | HTH~PRD_Mga~YSIRK_signal~PTZ00121~Gram_pos_anchor |
| 2 | Streptococcus | IsdB~YSIRK_signal~Glyco_hyd_65N_2~ATH1~PHA03378~Gram_pos_anchor |
