# Supplementary material for: YSIRK-G/S-directed translocation is required for Streptococcus suis to deliver diverse cell wall anchoring effectors contributing to bacterial pathogenicity
Source: Virulence. 2020 Nov 2;11(1):1539–56. doi: 10.1080/21505594.2020.1838740 (PMC7644249; doi:10.1080/21505594.2020.1838740)
Supplement: Supplemental Material [file KVIR_A_1838740_SM7169.zip › Supplementary Table S5.docx]

| **Table S5** Proteins with an N-terminal YSIRK motif but lacking LPxTG motif screened by domain-architecture retrieval. | | |
| --- | --- | --- |
| Sequences | Taxonomy | Domain Names |
| 1900 | Streptococcus pneumoniae | YSIRK_signal~RICH~PTZ00121~COG5263 |
| 1786 | Streptococcus | IsdB~YSIRK_signal~LacZ~Big_4~YabE~G5 |
| 1731 | Bacilli | YSIRK_signal~pullulan_Gpos |
| 1636 | Lactobacillales | YSIRK_signal~LamG~Sialidase |
| 1556 | cellular organisms | YSIRK_signal |
| 1424 | Bacteria | YSIRK_signal~Pgu1 |
| 877 | Staphylococcus | YSIRK_signal~MSCRAMM_SdrC~SasC_Mrp_aggreg~DUF1542~Smc~DUF1542 |
| 771 | Staphylococcus | YSIRK_signal~B~InfB~LysM |
| 571 | Bacteria | YSIRK_signal~MSCRAMM_SdrC |
| 568 | Firmicutes | YSIRK_signal~Rib |
| 563 | Staphylococcus | YSIRK_signal~B |
| 552 | Terrabacteria group | MSCRAMM_SdrC~YSIRK_signal |
| 480 | Streptococcus pneumoniae | YSIRK_signal~RICH~COG5263 |
| 453 | Staphylococcus | YSIRK_signal~B~LysM |
| 326 | Streptococcus | IsdB~YSIRK_signal~LacZ~Big_4~G5 |
| 325 | Staphylococcus | YSIRK_signal~PRK07764~SasC_Mrp_aggreg~DUF1542~PTZ00121~DUF1542~PRK08581 |
| 313 | Streptococcus | YSIRK_signal~Peptidases_S8_S53~PA~fn3_5 |
| 270 | Streptococcus pneumoniae | YSIRK_signal~PTZ00121~RICH~COG5263 |
| 247 | Streptococcus | YSIRK_signal~AlphaC_N~AlphaC_C~Rib |
| 237 | Streptococcus | YSIRK_signal~PRK11907~pullulan_Gpos~AmyAc_family~pullulan_Gpos~G5 |
| 233 | Staphylococcus | YSIRK_signal~B~PTZ00449~LysM |
| 231 | Streptococcus pneumoniae | YSIRK_signal~RICH~PTZ00121~RICH~COG5263 |
| 224 | Terrabacteria group | YSIRK_signal~MSCRAMM_SdrC~PRK08581~Abhydrolase |
| 223 | Terrabacteria group | YSIRK_signal~MSCRAMM_SdrC~SasC_Mrp_aggreg~DUF1542 |
| 196 | Bacillales | YSIRK_signal~PRK06347~LysM~NLPC_P60 |
| 190 | Staphylococcus | YSIRK_signal~Med15~SasC_Mrp_aggreg~DUF1542~PTZ00121~DUF1542~PRK08581 |
| 185 | Streptococcus pneumoniae | YSIRK_signal~RICH |
| 183 | Streptococcus pneumoniae | YSIRK_signal~PTZ00121~RICH~PTZ00449~COG5263 |
| 181 | Bacteria | YSIRK_signal~MSCRAMM_SdrD |
| 178 | Streptococcus pneumoniae | YSIRK_signal~RICH~PRK05035~COG5263 |
| 163 | Terrabacteria group | YSIRK_signal~PRK08581~Abhydrolase |
| 155 | Streptococcus | YSIRK_signal~GH_101_like~Glyco_hyd_101C~F5_F8_type_C |
| 150 | Streptococcus pneumoniae | YSIRK_signal~MSCRAMM_SdrD~LamG~Sialidase |
| 140 | Streptococcus pneumoniae | YSIRK_signal~RICH~PTZ00121~COG5263~glucan_65_rpt |
| 136 | Streptococcus pneumoniae | YSIRK_signal~PTZ00121~COG5263 |
| 134 | Staphylococcus | YSIRK_signal~MSCRAMM_SdrC~MDN1~Abhydrolase |
| 132 | Staphylococcus | YSIRK_signal~Med15~SasC_Mrp_aggreg~DUF1542~PRK08581 |
| 121 | Staphylococcus | YSIRK_signal~B~InfB~B~LysM |
| 120 | Lactobacillales | YSIRK_signal~G5~Peptidase_M26_N~Peptidase_M26_C |
| 120 | Streptococcus | MSCRAMM_SdrC~YSIRK_signal~LacZ~Big_4~G5 |
| 117 | Firmicutes | YSIRK_signal~PTZ00121 |
| 106 | Staphylococcus | YSIRK_signal~SasC_Mrp_aggreg~DUF1542~Smc |
| 105 | Bacilli | YSIRK_signal~SMC_prok_B |
| 102 | Staphylococcus | YSIRK_signal~MDN1~Abhydrolase |
| 102 | Streptococcus pneumoniae | YSIRK_signal~LamG~Sialidase~Trypan_PARP |
| 100 | Streptococcus pneumoniae | YSIRK_signal~RICH~YqiK~COG5263 |
| 99 | Streptococcus | YSIRK_signal~MSCRAMM_SdrC~GH18_chitinase-like |
| 96 | Firmicutes | YSIRK_signal~MucBP |
| 91 | Streptococcus | YSIRK_signal~glucan_65_rpt~COG4099 |
| 91 | Bacilli | YSIRK_signal~MSCRAMM_SdrC~PTZ00121~Abhydrolase |
| 90 | Streptococcus | MSCRAMM_SdrC~YSIRK_signal~Peptidases_S8_S53~PA~fn3_5~COG5022~FIVAR |
| 90 | Streptococcus pneumoniae | YSIRK_signal~hyperosmo_Ebh~DUF1542~Smc |
| 89 | Streptococcus pneumoniae | YSIRK_signal~RICH~PTZ00121~COG5263~CW_binding_1 |
| 86 | Streptococcus suis | YSIRK_signal~pullulan_Gpos~AmyAc_family~pullulan_Gpos~G5 |
| 85 | Streptococcus pneumoniae | YSIRK_signal~DUF4775~G5~RPT_S_cricet~Collagen~Glutenin_hmw~Collagen~RPT_S_cricet |
| 84 | Streptococcus | YSIRK_signal~MSCRAMM_SdrC~Peptidases_S8_S53~PA~fn3_5 |
| 83 | Streptococcus pneumoniae | YSIRK_signal~RICH~PTZ00121~RICH~PTZ00449~COG5263 |
| 79 | Streptococcus pneumoniae | YSIRK_signal~RICH~Smc~SMC_prok_A~RICH~PTZ00449~COG5263 |
| 78 | Streptococcus suis | YSIRK_signal~Mac-1~rne |
| 78 | Streptococcus | YSIRK_signal~PTZ00121~LacZ~Big_4~G5 |
| 77 | Bacilli | YSIRK_signal~MDN1 |
| 77 | Bacilli | YSIRK_signal~Smc |
| 75 | Staphylococcus | YSIRK_signal~MSCRAMM_SdrC~PTZ00482~Abhydrolase |
| 75 | Streptococcus | YSIRK_signal~lectin_L-type |
| 75 | Streptococcus pneumoniae | YSIRK_signal~PTZ00121~Chb |
| 75 | Staphylococcus | YSIRK_signal~B~PTZ00449 |
| 74 | Staphylococcus aureus | YSIRK_signal~PRK07764~SasC_Mrp_aggreg~DUF1542 |
| 74 | Staphylococcus | YSIRK_signal~B~LysM~LysM |
| 72 | Streptococcus | YSIRK_signal~rne~G5~Peptidase_M26_N~Peptidase_M26_C |
| 70 | Streptococcus pneumoniae | YSIRK_signal~RICH~PRK10819 |
| 70 | Streptococcus | YSIRK_signal~AlphaC_N~AlphaC_C |
| 69 | Streptococcus pneumoniae | YSIRK_signal~RICH~DUF5401~COG5263 |
| 69 | Terrabacteria group | YSIRK_signal~MSCRAMM_SdrC~Abhydrolase |
| 68 | Streptococcus suis | YSIRK_signal~GH18_chitinase-like~F5_F8_type_C~Big_3~TBPIP~FIVAR~G5 |
| 67 | Firmicutes | YSIRK_signal~hyperosmo_Ebh |
| 67 | Streptococcus pneumoniae | YSIRK_signal~RICH~PTZ00121 |
| 67 | Streptococcus pneumoniae | YSIRK_signal~termin_org_DnaJ~G5~Peptidase_M26_N~Glug~Peptidase_M26_C |
| 66 | Bacteria | YSIRK_signal~rne |
| 65 | Streptococcus pneumoniae | YSIRK_signal~PTZ00121~G5~Glutenin_hmw~Collagen~RPT_S_cricet~Collagen~RPT_S_cricet~Herpes_BLLF1~RPT_S_cricet |
| 64 | Staphylococcus | YSIRK_signal~PTZ00121~Abhydrolase |
| 63 | Streptococcus pneumoniae | YSIRK_signal~PTZ00121~G5~Glutenin_hmw~Collagen~RPT_S_cricet |
| 63 | Streptococcus | YSIRK_signal~MSCRAMM_SdrC~GH18_chitinase-like~F5_F8_type_C~Big_3~FIVAR |
| 60 | Streptococcus | YSIRK_signal~Abhydrolase~F5_F8_type_C~Thiol_cytolys_C |
| 60 | Streptococcus pneumoniae | YSIRK_signal~RICH~SMC_prok_A~COG5263 |
| 58 | Streptococcus pneumoniae | IsdB~YSIRK_signal~LacZ~Big_4 |
| 56 | Streptococcus | IsdB~YSIRK_signal~LacZ |
| 56 | Streptococcus pneumoniae | YSIRK_signal~RICH~PTZ00121~RICH~CW_binding_1~COG5263 |
| 56 | Streptococcus pneumoniae | YSIRK_signal~DUF5401~GAGBD~COG5263 |
| 56 | Firmicutes | YSIRK_signal~G5 |
| 56 | Streptococcus | YSIRK_signal~hyperosmo_Ebh~DUF1542 |
| 55 | Streptococcus pneumoniae | YSIRK_signal~RICH~Smc~COG5263~CW_binding_1 |
| 55 | Streptococcus pneumoniae | YSIRK_signal~PTZ00121~Chb~G5 |
| 54 | Streptococcus pneumoniae | YSIRK_signal~RICH~COG5263~glucan_65_rpt |
| 54 | Lactobacillales | IsdB~YSIRK_signal |
| 53 | Bacilli | YSIRK_signal~MSCRAMM_SdrC~Herpes_BLLF1 |
| 52 | Firmicutes | YSIRK_signal~DUF1542 |
| 52 | Firmicutes | YSIRK_signal~Rib~hyperosmo_Ebh~Rib |
| 52 | Streptococcus pneumoniae | YSIRK_signal~RICH~Smc~COG5263~glucan_65_rpt |
| 52 | Staphylococcus aureus | YSIRK_signal~PRK07764~SasC_Mrp_aggreg~DUF1542~PTZ00121~DUF1542 |
| 52 | Terrabacteria group | YSIRK_signal~MSCRAMM_SdrC~SasC_Mrp_aggreg |
| 51 | Streptococcus pneumoniae | YSIRK_signal~DUF4775~Glutenin_hmw~Collagen~RPT_S_cricet |
| 50 | Streptococcus | MSCRAMM_SdrC~YSIRK_signal~pullulan_Gpos |
| 49 | Streptococcus | MSCRAMM_SdrC~YSIRK_signal~SH3~GBS_Bsp-like~SH3~GBS_Bsp-like~SH3 |
| 49 | Streptococcus pneumoniae | YSIRK_signal~PTZ00121~RICH |
| 49 | Streptococcus | YSIRK_signal~SH3~GBS_Bsp-like~SH3 |
| 48 | Streptococcus pneumoniae | YSIRK_signal~ATP-synt_B~LacZ~Big_4~YabE~G5 |
| 47 | Bacilli | MSCRAMM_SdrD~YSIRK_signal |
| 47 | Streptococcus pneumoniae | YSIRK_signal~RICH~PRK14948~RICH |
| 46 | Bacilli | YSIRK_signal~PRK07764~Herpes_BLLF1 |
| 46 | Staphylococcus | YSIRK_signal~MSCRAMM_SdrC~GET2~Abhydrolase |
| 46 | Streptococcus | MSCRAMM_SdrC~YSIRK_signal~SH3~GBS_Bsp-like~SH3 |
| 45 | Streptococcus pneumoniae | YSIRK_signal~PTZ00121~G5~Glutenin_hmw~Collagen~RPT_S_cricet~Collagen~RPT_S_cricet~Cornifin |
| 45 | Streptococcus pneumoniae | YSIRK_signal~RICH~Smc~RICH~PTZ00449~COG5263 |
| 44 | Streptococcus suis | YSIRK_signal~INTAP~GAG_Lyase |
| 43 | Staphylococcus aureus | YSIRK_signal~Med15~SasC_Mrp_aggreg~DUF1542~PTZ00121~DUF1542~Herpes_BLLF1 |
| 43 | Streptococcus pneumoniae | YSIRK_signal~DUF4775~Glutenin_hmw~Collagen~RPT_S_cricet~Collagen~RPT_S_cricet |
| 43 | Streptococcus | YSIRK_signal~lectin_L-type~FIVAR |
| 43 | Staphylococcus aureus | YSIRK_signal~PRK07764~SasC_Mrp_aggreg |
| 42 | Streptococcus | YSIRK_signal~2A1904~G5~Peptidase_M26_N~Peptidase_M26_C |
| 42 | Streptococcus agalactiae | MSCRAMM_SdrC~YSIRK_signal~hyperosmo_Ebh~Rib~He_PIG~Rib |
| 42 | Streptococcus pneumoniae | YSIRK_signal~RICH~PRK10263 |
| 41 | Streptococcus | YSIRK_signal~GA-like~IgG_binding_B |
| 41 | Streptococcus | YSIRK_signal~PRK11907~pullulan_Gpos |
| 41 | Streptococcus | YSIRK_signal~PHA03255~pullulan_Gpos |
| 41 | Staphylococcus aureus | YSIRK_signal~B~Trypan_PARP |
| 39 | Streptococcus pneumoniae | YSIRK_signal~RICH~Smc~RICH~PTZ00449 |
| 39 | Lactobacillales | YSIRK_signal~IsdB |
| 39 | Streptococcus | MSCRAMM_SdrC~YSIRK_signal~CshA_fibril_rpt |
| 39 | Firmicutes | YSIRK_signal~hyperosmo_Ebh~Rib |
| 39 | Bacilli | YSIRK_signal~MSCRAMM_SdrC~Rib |
| 38 | Staphylococcus | YSIRK_signal~PRK08581~SasC_Mrp_aggreg~DUF1542 |
| 38 | Streptococcus pneumoniae | YSIRK_signal~G5~Peptidase_M26_N~Glug~Peptidase_M26_C |
| 37 | Staphylococcus haemolyticus | YSIRK_signal~MSCRAMM_SdrD~MG1~Herpes_BLLF1~MG1 |
| 37 | Streptococcus pneumoniae | YSIRK_signal~DUF4775~G5~Glutenin_hmw~Collagen~RPT_S_cricet |
| 37 | Streptococcus suis | YSIRK_signal~rne~Peptidase_M26_C |
| 37 | Streptococcus | YSIRK_signal~MSCRAMM_SdrC~SH3~GBS_Bsp-like~Peptidase_M15~SH3 |
| 37 | Streptococcus | YSIRK_signal~G5~zmp_18_rpt~G5~FIVAR~Peptidase_M26_N~Peptidase_M26_C |
| 37 | Bacilli | YSIRK_signal~Abhydrolase |
| 37 | Streptococcus suis | YSIRK_signal~predic_Ig_block~PHA03247~Peptidase_M26_N~FhaB~Peptidase_M26_C |
| 36 | Staphylococcus | YSIRK_signal~SasC_Mrp_aggreg~DUF1542~PRK05035~DUF1542~PRK08581 |
| 36 | Streptococcus pneumoniae | YSIRK_signal~PTZ00121~G5~Collagen~RPT_S_cricet~Glutenin_hmw~Collagen~RPT_S_cricet |
| 36 | Streptococcus pneumoniae | YSIRK_signal~MSCRAMM_SdrC~GH18_chitinase-like~F5_F8_type_C~Big_3 |
| 36 | Streptococcus pneumoniae | YSIRK_signal~PTZ00121~G5~Glutenin_hmw~Collagen~RPT_S_cricet~Collagen~RPT_S_cricet |
| 36 | Streptococcus pneumoniae | YSIRK_signal~DUF4775~G5~RPT_S_cricet~Glutenin_hmw~Collagen~RPT_S_cricet~PHA03247 |
| 35 | Terrabacteria group | YSIRK_signal~MSCRAMM_SdrC~hyperosmo_Ebh |
| 35 | Staphylococcus | YSIRK_signal~MSCRAMM_SdrC~235kDa-fam~Abhydrolase |
| 34 | Staphylococcus | YSIRK_signal~Med15~SasC_Mrp_aggreg~DUF1542~PTZ00121~PRK05035~DUF1542~PRK08581 |
| 34 | Streptococcus | MSCRAMM_SdrC~YSIRK_signal~SH3 |
| 33 | Streptococcus | YSIRK_signal~MSCRAMM_SdrC~Peptidases_S8_S53~PA~fn3_5~FlgD_ig~FIVAR |
| 32 | Streptococcus suis | IsdB~YSIRK_signal~Peptidases_S8_S53~PA~fn3_5 |
| 32 | Streptococcus pneumoniae | YSIRK_signal~RICH~PTZ00121~RICH |
| 32 | Streptococcus | YSIRK_signal~IsdB~pullulan_Gpos |
| 32 | Streptococcus equi | YSIRK_signal~SMC_prok_B~GA-like |
| 31 | Streptococcus pneumoniae | YSIRK_signal~hyperosmo_Ebh~Periplasmic_Binding_Protein_Type_1~hyperosmo_Ebh~PTZ00121~SbcC |
| 31 | Streptococcus suis | YSIRK_signal~GAGBD~PHA03247~G5~COG4932 |
| 31 | Bacilli | YSIRK_signal~SSURE |
| 31 | Streptococcus pneumoniae | YSIRK_signal~hyperosmo_Ebh~Periplasmic_Binding_Protein_Type_1~hyperosmo_Ebh~DUF1542 |
| 31 | Streptococcus pneumoniae | YSIRK_signal~CDC27~G5~Collagen~Glutenin_hmw~Collagen~RPT_S_cricet |
| 30 | Staphylococcus | YSIRK_signal~B~MSCRAMM_SdrC~LysM |
| 30 | Streptococcus | YSIRK_signal~SucB_Actino~Trypan_PARP |
| 30 | Streptococcus | YSIRK_signal~MSCRAMM_SdrC~COG3942~SH3~GBS_Bsp-like~SH3 |
| 30 | Streptococcus | YSIRK_signal~Mac-1 |
| 29 | Staphylococcus | YSIRK_signal~MSCRAMM_SdrC~SasC_Mrp_aggreg~DUF1542~PTZ00121~DUF1542 |
| 29 | Staphylococcus | YSIRK_signal~MSCRAMM_SdrC~SasC_Mrp_aggreg~DUF1542~Smc |
| 29 | Streptococcus suis | YSIRK_signal~GAGBD~PHA03247~G5 |
| 29 | Streptococcus pneumoniae | YSIRK_signal~PTZ00121~G5~Glutenin_hmw~Collagen~RPT_S_cricet~Herpes_BLLF1~RPT_S_cricet |
| 29 | Staphylococcus pseudintermedius | YSIRK_signal~Herpes_BLLF1~GH_101_like~Glyco_hyd_101C |
| 28 | Streptococcus pneumoniae | YSIRK_signal~DUF4775~G5~Glutenin_hmw~Collagen~RPT_S_cricet~Collagen~RPT_S_cricet~Herpes_BLLF1~RPT_S_cricet |
| 28 | Streptococcus | YSIRK_signal~MSCRAMM_SdrC~AfuC~Calx-beta~G5 |
| 28 | Bacilli | YSIRK_signal~MSCRAMM_SdrC~PTZ00449 |
| 28 | Lactobacillales | YSIRK_signal~IsdB~hyperosmo_Ebh |
| 28 | Staphylococcus | MSCRAMM_SdrC~YSIRK_signal~FctA~Antigen_C~FctA~MSCRAMM_SdrC |
| 28 | Lactobacillales | YSIRK_signal~PTZ00121~DUF1542 |
| 28 | Bacilli | YSIRK_signal~IsdH_HarA |
| 28 | Streptococcus pneumoniae | YSIRK_signal~hyperosmo_Ebh~PTZ00121~DUF1542 |
| 27 | Staphylococcus | YSIRK_signal~PRK07764~SasC_Mrp_aggreg~DUF1542~PRK08581 |
| 27 | Staphylococcus pseudintermedius | YSIRK_signal~PRK14949~Abhydrolase |
| 27 | Streptococcus suis | YSIRK_signal~GH18_chitinase-like~F5_F8_type_C~Big_3~TBPIP~G5 |
| 27 | Streptococcus pneumoniae | YSIRK_signal~RICH~Smc~PRK00409~RICH~PTZ00449~COG5263 |
| 27 | Streptococcus pneumoniae | YSIRK_signal~DUF4775~Collagen~Glutenin_hmw~Collagen~RPT_S_cricet |
| 26 | Streptococcus suis | YSIRK_signal~IsdB~pullulan_Gpos~AmyAc_family~pullulan_Gpos~G5 |
| 26 | Streptococcus pneumoniae | YSIRK_signal~rne~hyperosmo_Ebh |
| 26 | Streptococcus suis | YSIRK_signal~G5~Peptidase_M26_N~FhaB~Peptidase_M26_C |
| 26 | Staphylococcus | YSIRK_signal~B~2A1904~LysM |
| 26 | Lactobacillales | YSIRK_signal~MSCRAMM_SdrC~CshA_fibril_rpt |
| 26 | Streptococcus | YSIRK_signal~F5_F8_type_C~Thiol_cytolysin~Thiol_cytolys_C |
| 26 | Streptococcus | YSIRK_signal~MSCRAMM_SdrC~LacZ~Big_4~G5 |
| 26 | Streptococcus | YSIRK_signal~YabE~G5~FIVAR~DUF745~Peptidase_M26_N~Peptidase_M26_C |
| 26 | Enterococcus | YSIRK_signal~Rib~CshA_fibril_rpt |
| 25 | Staphylococcus aureus | MSCRAMM_SdrC~YSIRK_signal~AlphaC_N |
| 25 | Streptococcus | YSIRK_signal~PRK11633~F5_F8_type_C~Glyco_hydro_20b~GH20_hexosaminidase~F5_F8_type_C~Glyco_hydro_20b~NAGidase~F5_F8_type_C |
| 25 | Bacilli | YSIRK_signal~MSCRAMM_SdrC~MucBP~PTZ00449 |
| 24 | Staphylococcaceae | YSIRK_signal~MDN1~MSCRAMM_SdrD~He_PIG |
| 24 | Streptococcus | IsdB~YSIRK_signal~pullulan_Gpos |
| 24 | Staphylococcus | YSIRK_signal~SasC_Mrp_aggreg~DUF1542 |
| 24 | Bacteria | YSIRK_signal~Peptidases_S8_S53 |
| 24 | Streptococcus equi | YSIRK_signal~CBM_4_9~LamG~Sialidase |
| 24 | Streptococcus suis | YSIRK_signal~GH18_chitinase-like~F5_F8_type_C~Big_3~FIVAR~G5 |
| 24 | Bacilli | MSCRAMM_SdrC~YSIRK_signal~hyperosmo_Ebh |
| 24 | Streptococcus | YSIRK_signal~PRK10263~G5~Peptidase_M26_N~Peptidase_M26_C |
| 24 | Firmicutes | YSIRK_signal~MSCRAMM_SdrC~pullulan_Gpos |
| 22 | Staphylococcus aureus | YSIRK_signal~MSCRAMM_SdrC~DUF612~Abhydrolase |
| 22 | Streptococcus pneumoniae | YSIRK_signal~DUF4775~G5~RPT_S_cricet~Collagen~Glutenin_hmw~Collagen~RPT_S_cricet~PHA03247 |
| 22 | Staphylococcus | YSIRK_signal~MSCRAMM_SdrC~FctA~Antigen_C~FctA~MSCRAMM_SdrC |
| 22 | Staphylococcus epidermidis | YSIRK_signal~MSCRAMM_SdrC~Herpes_BLLF1~MDN1 |
| 21 | Streptococcus pneumoniae | YSIRK_signal~PTZ00121~G5~Glutenin_hmw~RPT_S_cricet~Glutenin_hmw~Collagen~RPT_S_cricet~Herpes_BLLF1~RPT_S_cricet |
| 21 | Staphylococcus epidermidis | YSIRK_signal~SasC_Mrp_aggreg~DUF1542~Smc~DUF1542 |
| 21 | Streptococcus pneumoniae | YSIRK_signal~RICH~PTZ00121~RICH~Spc7~COG5263 |
| 21 | Terrabacteria group | YSIRK_signal~MSCRAMM_SdrC~SasC_Mrp_aggreg~DUF1542~PTZ00121 |
| 21 | Streptococcus pneumoniae | YSIRK_signal~PRK11633~PTZ00121~Chb |
| 21 | Streptococcus pneumoniae | YSIRK_signal~GH_101_like~Glyco_hyd_101C |
| 21 | Streptococcus | YSIRK_signal~LacZ~Big_4~G5 |
| 21 | Firmicutes | YSIRK_signal~MSCRAMM_SdrC~GH18_chitinase-like~F5_F8_type_C~Big_3~FIVAR~G5 |
| 20 | Lactobacillales | YSIRK_signal~PRK13108 |
| 20 | Staphylococcus haemolyticus | YSIRK_signal~PRK08581~SasC_Mrp_aggreg~DUF1542~PTZ00121 |
| 20 | Staphylococcus aureus | YSIRK_signal~SasC_Mrp_aggreg~DUF1542~PTZ00121~DUF1542~PRK08581 |
| 20 | Streptococcus pneumoniae | YSIRK_signal~IsdB~hyperosmo_Ebh~DUF1542~Smc |
| 20 | Streptococcus pneumoniae | YSIRK_signal~PRK14949~hyperosmo_Ebh~Periplasmic_Binding_Protein_Type_1~hyperosmo_Ebh~DUF1542 |
| 20 | Streptococcus | YSIRK_signal~rne~GH43_62_32_68_117_130~PTZ00449 |
| 20 | Streptococcus sanguinis | YSIRK_signal~PRK07003~Peptidases_S8_S53~PA~fn3_5~FlgD_ig~FIVAR |
| 19 | Streptococcus | YSIRK_signal~LamG |
| 19 | Streptococcus suis | YSIRK_signal~Mac-1~DUF4775 |
| 19 | Streptococcus | MSCRAMM_SdrC~YSIRK_signal~Peptidases_S8_S53 |
| 19 | Streptococcus | YSIRK_signal~rne~vWFA |
| 19 | Streptococcus pneumoniae | YSIRK_signal~PTZ00121~G5~Glutenin_hmw~RPT_S_cricet |
| 19 | Staphylococcus | YSIRK_signal~MSCRAMM_SdrD~MG1 |
| 19 | Streptococcus pneumoniae | YSIRK_signal~MSCRAMM_SdrC~GH18_chitinase-like~F5_F8_type_C |
| 19 | Streptococcus pneumoniae | YSIRK_signal~PHA03291 |
| 19 | Staphylococcus aureus | YSIRK_signal~PRK07764~SasC_Mrp_aggreg~DUF1542~PRK05035~DUF1542~PRK08581 |
| 19 | Streptococcus | YSIRK_signal~PHA03169~G5~Peptidase_M26_N~Peptidase_M26_C |
| 19 | Bacilli | YSIRK_signal~MSCRAMM_SdrC~MucBP |
| 19 | Streptococcus | YSIRK_signal~PRK07764~Peptidases_S8_S53~PA~fn3_5~FlgD_ig~FIVAR |
| 18 | Streptococcus | YSIRK_signal~rne~CshA_fibril_rpt~repeat_SSSPR51 |
| 18 | Streptococcus pneumoniae | YSIRK_signal~MSCRAMM_SdrC~GH18_chitinase-like~Big_3~FIVAR |
| 18 | Streptococcus pneumoniae | YSIRK_signal~RICH~PTZ00121~CW_binding_1~COG5263 |
| 18 | Staphylococcus simulans | MSCRAMM_SdrC~YSIRK_signal~COG5651~G5~DUF2460~G5 |
| 18 | Staphylococcus chromogenes | YSIRK_signal~PTZ00121~He_PIG~MSCRAMM_SdrC |
| 18 | Bacilli | YSIRK_signal~vWFA |
| 18 | Streptococcus | YSIRK_signal~Glutenin_hmw~G5~Peptidase_M26_N~Peptidase_M26_C |
| 18 | Streptococcus | MSCRAMM_SdrC~YSIRK_signal~SH3~GBS_Bsp-like |
| 18 | Terrabacteria group | YSIRK_signal~DUF1542~Smc~DUF1542 |
| 18 | Streptococcus | YSIRK_signal~MSCRAMM_SdrC~Glyco_hyd_65N_2~ATH1 |
| 18 | Streptococcus | YSIRK_signal~Tryp_SPc~GH_101_like~Glyco_hyd_101C~F5_F8_type_C |
| 18 | Staphylococcus epidermidis | YSIRK_signal~PRK07764~Herpes_BLLF1~MDN1 |
| 18 | Streptococcus pneumoniae | YSIRK_signal~RICH~SbcC~RICH~PTZ00449~COG5263 |
| 17 | Bacilli | YSIRK_signal~2A1904 |
| 17 | Streptococcus | YSIRK_signal~MSCRAMM_SdrC~Abhydrolase~G5 |
| 17 | Streptococcus | YSIRK_signal~rne~Peptidases_S8_S53~PA~fn3_5~FIVAR |
| 17 | Streptococcus pneumoniae | YSIRK_signal~DUF4775~G5~Collagen~Glutenin_hmw~Collagen~RPT_S_cricet~Glutenin_hmw~Collagen~RPT_S_cricet |
| 17 | Lactobacillales | YSIRK_signal~PHA03255 |
| 17 | Streptococcus pneumoniae | YSIRK_signal~RICH~COG5263~CW_binding_1 |
| 17 | Streptococcus pneumoniae | YSIRK_signal~RICH~PTZ00121~RICH~Topo_C_assoc~COG5263 |
| 16 | Lactobacillales | YSIRK_signal~PRK11907 |
| 16 | Staphylococcus | YSIRK_signal~MSCRAMM_SdrC~PTZ00112~Abhydrolase |
| 16 | Streptococcus pneumoniae | YSIRK_signal~GH_101_like |
| 16 | Streptococcus | YSIRK_signal~hyperosmo_Ebh~PTZ00121 |
| 16 | Streptococcus | YSIRK_signal~DedD~CBM6-CBM35-CBM36_like~beta_helix_1~Herpes_BLLF1 |
| 16 | Streptococcus | YSIRK_signal~MSCRAMM_SdrC~Peptidases_S8_S53 |
| 16 | Streptococcus suis | YSIRK_signal~PRK11907~GAG_Lyase |
| 16 | Streptococcus pneumoniae | YSIRK_signal~PTZ00121~Collagen~RPT_S_cricet~Collagen~RPT_S_cricet~Glutenin_hmw~Collagen~RPT_S_cricet |
| 16 | Staphylococcus | YSIRK_signal~PRK08581~YncE~MSCRAMM_SdrC~Herpes_LMP1 |
| 16 | Staphylococcus | YSIRK_signal~SasC_Mrp_aggreg~FIVAR~hyperosmo_Ebh |
| 16 | Streptococcus pneumoniae | YSIRK_signal~G5~Glutenin_hmw~Collagen~RPT_S_cricet~Herpes_BLLF1~RPT_S_cricet |
| 16 | Staphylococcus aureus | YSIRK_signal~Med15~SasC_Mrp_aggreg~DUF1542~PRK05035~DUF1542~PRK08581 |
| 16 | Streptococcus pneumoniae | YSIRK_signal~RICH~PTZ00121~RICH~PTZ00449 |
| 16 | Streptococcus pneumoniae | YSIRK_signal~RICH~SbcC~COG5263 |
| 16 | Streptococcus pneumoniae | YSIRK_signal~DUF4775~G5~Glutenin_hmw~Collagen~PHA03247 |
| 16 | Staphylococcus simulans | MSCRAMM_SdrC~YSIRK_signal~rne~lectin_L-type~He_PIG~PRK11281~PTZ00121 |
| 15 | Staphylococcus | YSIRK_signal~PRK07764~PHA03247 |
| 15 | Streptococcus | YSIRK_signal~LamG~Sialidase~COG5263 |
| 15 | Streptococcus | MSCRAMM_SdrC~YSIRK_signal~Peptidases_S8_S53~PA~fn3_5 |
| 15 | Staphylococcus aureus | YSIRK_signal~B~MDN1~B |
| 15 | Staphylococcus | YSIRK_signal~MSCRAMM_SdrC~SasC_Mrp_aggreg~DUF1542~SMC_prok_B~DUF1542 |
| 15 | Streptococcus pneumoniae | YSIRK_signal~DUF4775~G5~Glutenin_hmw~Collagen~RPT_S_cricet~Herpes_BLLF1~RPT_S_cricet |
| 15 | Streptococcus pneumoniae | YSIRK_signal~RICH~PTZ00121~RICH~Spc7~CW_binding_1~COG5263 |
| 15 | Bacilli | MSCRAMM_SdrC~YSIRK_signal~PTZ00121 |
| 15 | Streptococcus pneumoniae | YSIRK_signal~RICH~PTZ00121~COG5263~CW_binding_1~glucan_65_rpt |
| 15 | Streptococcus pneumoniae | YSIRK_signal~DUF4775~G5~Collagen~RPT_S_cricet |
| 15 | Streptococcus | YSIRK_signal~PRK13335~F5_F8_type_C~Glyco_hydro_20b~GH20_hexosaminidase~F5_F8_type_C~Glyco_hydro_20b~NAGidase~F5_F8_type_C |
| 15 | Staphylococcus aureus | YSIRK_signal~TonB~AlphaC_N |
| 15 | Streptococcus pyogenes | YSIRK_signal~APG6 |
| 15 | Streptococcus | YSIRK_signal~PRK07003~pullulan_Gpos |
| 15 | Streptococcus suis | YSIRK_signal~INTAP~Lyase_8_N |
| 15 | Streptococcus | YSIRK_signal~GbpC |
| 15 | Streptococcus | MSCRAMM_SdrC~YSIRK_signal~Sen15~CshA_fibril_rpt |
| 15 | Staphylococcus | YSIRK_signal~PRK07764~SasC_Mrp_aggreg~PTZ00121~DUF1542 |
| 14 | Staphylococcus | YSIRK_signal~PTZ00121~He_PIG |
| 14 | Streptococcus sobrinus | MSCRAMM_SdrC~YSIRK_signal~glucan_65_rpt~COG4099 |
| 14 | Bacilli | YSIRK_signal~PRK08581 |
| 14 | Streptococcus pneumoniae | YSIRK_signal~RICH~SMC_prok_A~COG5263~glucan_65_rpt |
| 14 | Streptococcus pneumoniae | YSIRK_signal~RICH~predic_Ig_block |
| 14 | Streptococcus | YSIRK_signal~GH18_chitinase-like~F5_F8_type_C~Big_3~Amelogenin |
| 14 | Staphylococcus | YSIRK_signal~SasC_Mrp_aggreg~DUF1542~Smc~DUF1542~SbcC~DUF1542 |
| 13 | Staphylococcus | YSIRK_signal~PRK07764~SasC_Mrp_aggreg~DUF1542~Herpes_BLLF1 |
| 13 | Streptococcus | YSIRK_signal~MSCRAMM_SdrC~SH3~GBS_Bsp-like~SH3 |
| 13 | Streptococcus pneumoniae | YSIRK_signal~PTZ00121~RICH~PTZ00449 |
| 13 | Streptococcus | YSIRK_signal~MSCRAMM_SdrC~SH3~GBS_Bsp-like |
| 13 | Streptococcus pneumoniae | YSIRK_signal~DUF4775~G5~Glutenin_hmw~Collagen~RPT_S_cricet~Collagen~RPT_S_cricet |
| 13 | Staphylococcus aureus | YSIRK_signal~Med15~SasC_Mrp_aggreg~DUF1542~PTZ00121~DUF1542 |
| 13 | Streptococcus | YSIRK_signal~G5~termin_org_DnaJ~G5~Peptidase_M26_N~Peptidase_M26_C |
| 13 | Staphylococcus haemolyticus | YSIRK_signal~Mplasa_alph_rch~MG1 |
| 13 | Staphylococcus | YSIRK_signal~PRK07764 |
| 13 | Streptococcus pneumoniae | YSIRK_signal~PRK05035~G5~Glutenin_hmw~Collagen~RPT_S_cricet~Herpes_BLLF1~RPT_S_cricet |
| 12 | Streptococcus pneumoniae | YSIRK_signal~DUF4775~G5~RPT_S_cricet~Glutenin_hmw~Collagen~RPT_S_cricet |
| 12 | Lactobacillales | MSCRAMM_SdrC~YSIRK_signal~pullulan_Gpos~AmyAc_family~pullulan_Gpos |
| 12 | Bacilli | YSIRK_signal~PTZ00121~CshA_fibril_rpt~repeat_SSSPR51 |
| 12 | Streptococcus | YSIRK_signal~Tryp_SPc~Trypan_PARP~GH_101_like~Glyco_hyd_101C~F5_F8_type_C~pullulan_Gpos |
| 12 | Streptococcus | YSIRK_signal~rne~CshA_fibril_rpt |
| 12 | Streptococcus pneumoniae | YSIRK_signal~2A1904~G5~Peptidase_M26_N~Glug~Peptidase_M26_C |
| 12 | Streptococcus pneumoniae | YSIRK_signal~DUF4775~G5~Glutenin_hmw~RPT_S_cricet~2A1904~Glutenin_hmw~Collagen~RPT_S_cricet |
| 12 | Streptococcus suis | YSIRK_signal~PHA03247~Peptidase_M26_N~FhaB~Peptidase_M26_C |
| 12 | Streptococcus | YSIRK_signal~GH18_chitinase-like~F5_F8_type_C~Big_3~PTZ00449~Amelogenin |
| 12 | Streptococcus | YSIRK_signal~AfuC~Calx-beta~G5 |
| 12 | Bacilli | MSCRAMM_SdrC~YSIRK_signal~hyperosmo_Ebh~Rib |
| 12 | Streptococcus pneumoniae | YSIRK_signal~DUF4775~G5~Collagen |
| 12 | Streptococcus agalactiae | MSCRAMM_SdrC~YSIRK_signal~G5~Collagen~RPT_S_cricet |
| 11 | Staphylococcus aureus | YSIRK_signal~B~PRK04195~LysM |
| 11 | Streptococcus pneumoniae | MSCRAMM_SdrC~YSIRK_signal~hyperosmo_Ebh~DUF1542~Smc |
| 11 | Streptococcus pneumoniae | IsdB~YSIRK_signal~LacZ~Big_4~mycoplas_M_dom~YabE~G5 |
| 11 | Staphylococcus | YSIRK_signal~MDN1~DUF2981 |
| 11 | Streptococcus pneumoniae | YSIRK_signal~G5~Glutenin_hmw~Collagen~RPT_S_cricet~Collagen~RPT_S_cricet~Herpes_BLLF1~RPT_S_cricet |
| 11 | Staphylococcus aureus | YSIRK_signal~MSCRAMM_SdrC~FAP~SasC_Mrp_aggreg~DUF1542 |
| 11 | Streptococcus | YSIRK_signal~tolA_full~GH18_chitinase-like~F5_F8_type_C~Big_3~FIVAR |
| 11 | Lactobacillales | YSIRK_signal~PRK08026 |
| 11 | Streptococcus pneumoniae | YSIRK_signal~PTZ00121~RICH~COG5263~CW_binding_1 |
| 11 | Streptococcus pneumoniae | YSIRK_signal~RICH~Smc~MIP-T3~COG5263 |
| 11 | Staphylococcus | YSIRK_signal~PRK08581~YncE |
| 11 | Staphylococcus epidermidis | YSIRK_signal~PHA03247~MDN1 |
| 11 | Staphylococcus aureus | YSIRK_signal~PRK07764~SasC_Mrp_aggreg~DUF1542~PTZ00121~DUF1542~Herpes_BLLF1 |
| 11 | Streptococcus | YSIRK_signal~Neuromodulin~pullulan_Gpos |
| 11 | Streptococcus | YSIRK_signal~MSCRAMM_SdrC~LamG~Sialidase |
| 11 | Streptococcus | MSCRAMM_SdrC~YSIRK_signal~LacZ |
| 11 | Streptococcus | YSIRK_signal~G5~Herpes_BLLF1~G5~FIVAR~Peptidase_M26_N~Peptidase_M26_C |
| 11 | Lactobacillales | YSIRK_signal~GA-like |
| 11 | Streptococcus pneumoniae | YSIRK_signal~GREB1~hyperosmo_Ebh~Periplasmic_Binding_Protein_Type_1~hyperosmo_Ebh~DUF1542 |
| 11 | Streptococcus suis | YSIRK_signal~Mac-1~AIF-MLS |
| 11 | Bacilli | YSIRK_signal~DUF5011 |
| 11 | Lactobacillales | YSIRK_signal~Peptidase_M26_C |
| 10 | Staphylococcus | YSIRK_signal~PRK08581~YncE~PHA03169~Herpes_LMP1 |
| 10 | Streptococcus gordonii | YSIRK_signal~YabE~G5~zmp_18_rpt~G5~FIVAR~Peptidase_M26_N~Peptidase_M26_C |
| 10 | Bacteria | YSIRK_signal~MSCRAMM_SdrC~DUF1542 |
| 10 | Streptococcus oralis | YSIRK_signal~YabE~G5~FIVAR~Peptidase_M26_N~Peptidase_M26_C |
| 10 | Streptococcus pneumoniae | YSIRK_signal~DUF4775~G5~Glutenin_hmw~Collagen |
| 10 | Streptococcus pneumoniae | MSCRAMM_SdrC~YSIRK_signal~LacZ~Big_4~YabE~G5 |
| 10 | Staphylococcus haemolyticus | YSIRK_signal~PRK08581~MSCRAMM_SdrC~SdrG_C_C |
| 10 | Streptococcus | MSCRAMM_SdrC~YSIRK_signal~SH3~GBS_Bsp-like~Peptidase_M15~SH3 |
| 10 | Streptococcus | YSIRK_signal~PRK07003~GBS_Bsp-like~Peptidase_C39_like |
| 10 | Streptococcus pneumoniae | YSIRK_signal~DUF4775~G5~Glutenin_hmw~RPT_S_cricet |
| 10 | Lactobacillales | YSIRK_signal~PHA03247 |
| 10 | Lactobacillus acidophilus | MSCRAMM_SdrD~YSIRK_signal~Herpes_BLLF1 |
| 10 | Streptococcus | YSIRK_signal~G5~FIVAR~DUF745~Peptidase_M26_N~Peptidase_M26_C |
| 10 | Streptococcus | YSIRK_signal~CBM6-CBM35-CBM36_like~beta_helix_1~Herpes_BLLF1 |
| 10 | Streptococcus pneumoniae | YSIRK_signal~MSCRAMM_SdrD~LamG |
| 10 | Streptococcus | YSIRK_signal~FctA~Antigen_C~FctA~Antigen_C~FctA~Antigen_C~FctA~Antigen_C~FctA~Antigen_C~FctA~Antigen_C~FctA |
| 10 | Staphylococcus aureus | YSIRK_signal~B~CobT |
| 9 | Staphylococcus aureus | YSIRK_signal~MSCRAMM_SdrC~GET2 |
| 9 | Streptococcus | YSIRK_signal~pullulan_Gpos~COG5263 |
| 9 | Streptococcus mitis | YSIRK_signal~DUF4775~Chb~G5~COG5263 |
| 9 | Streptococcus | YSIRK_signal~G5~PRK08691~G5~Peptidase_M26_N~Peptidase_M26_C |
| 9 | Lactobacillales | YSIRK_signal~MSCRAMM_SdrC~PTZ00121 |
| 9 | Streptococcus | YSIRK_signal~PHA03255~Peptidases_S8_S53~PA~fn3_5~FlgD_ig~FIVAR |
| 9 | Streptococcus | YSIRK_signal~G5~rne~G5~Peptidase_M26_N~Peptidase_M26_C |
| 9 | Streptococcus gordonii | YSIRK_signal~GH18_chitinase-like~F5_F8_type_C~Big_3~FIVAR |
| 9 | Streptococcus pneumoniae | YSIRK_signal~PTZ00121~GH18_chitinase-like~F5_F8_type_C~Big_3 |
| 9 | Streptococcus pneumoniae | YSIRK_signal~PRK13108~hyperosmo_Ebh~Periplasmic_Binding_Protein_Type_1~hyperosmo_Ebh~DUF1542 |
| 9 | Streptococcus pneumoniae | YSIRK_signal~RICH~Smc~CW_binding_1~COG5263 |
| 9 | Bacilli | YSIRK_signal~CshA_fibril_rpt~repeat_SSSPR51 |
| 9 | Streptococcus pneumoniae | YSIRK_signal~RICH~PTZ00108~COG5263 |
| 9 | Streptococcus pneumoniae | YSIRK_signal~DUF4775~Collagen~RPT_S_cricet~Glutenin_hmw~Collagen~RPT_S_cricet |
| 9 | Staphylococcus | YSIRK_signal~MSCRAMM_SdrC~SasC_Mrp_aggreg~DUF1542~SMC_N~DUF1542 |
| 9 | Streptococcus | YSIRK_signal~G5~zmp_18_rpt~G5~FIVAR~Peptidase_M26_N~FhaB~Peptidase_M26_C |
| 9 | Bacilli | YSIRK_signal~PTZ00121~CshA_fibril_rpt |
| 9 | Streptococcus | PRK06347~YSIRK_signal~pullulan_Gpos |
| 9 | Streptococcus | YSIRK_signal~G5~zmp_18_rpt~G5~FIVAR~Peptidase_M26_N~Glug~Peptidase_M26_C |
| 9 | Staphylococcus aureus | YSIRK_signal~MSCRAMM_SdrC~MSCRAMM_SdrD~Abhydrolase |
| 9 | Streptococcus | YSIRK_signal~DUF4775~G5 |
| 9 | Firmicutes | YSIRK_signal~Trypan_PARP~F5_F8_type_C~Glyco_hydro_20b~GH20_hexosaminidase~F5_F8_type_C~Glyco_hydro_20b~NAGidase~F5_F8_type_C |
| 9 | Streptococcus | YSIRK_signal~SMC_prok_A |
| 9 | Streptococcus pneumoniae | YSIRK_signal~RICH~PTZ00121~RICH~CW_binding_1 |
| 9 | Staphylococcus | YSIRK_signal~IsdH_HarA~MSCRAMM_SdrC |
| 9 | Aerococcus | YSIRK_signal~lectin_L-type~G5 |
| 9 | Streptococcus | YSIRK_signal~MSCRAMM_SdrC~Chb |
| 8 | Streptococcus pneumoniae | YSIRK_signal~GREB1~hyperosmo_Ebh~DUF1542 |
| 8 | Streptococcus | YSIRK_signal~Endomucin~pullulan_Gpos |
| 8 | Bacilli | YSIRK_signal~Tryp_SPc |
| 8 | Streptococcus pneumoniae | YSIRK_signal~DUF4775~G5~RPT_S_cricet~Collagen~Glutenin_hmw~RPT_S_cricet~Collagen~RPT_S_cricet |
| 8 | Streptococcus | YSIRK_signal~MSCRAMM_SdrC~F5_F8_type_C~Glyco_hydro_20b~GH20_hexosaminidase~F5_F8_type_C~Glyco_hydro_20b~NAGidase~F5_F8_type_C |
| 8 | Streptococcus | MSCRAMM_SdrC~YSIRK_signal~SH3~GBS_Bsp-like~SH3~GBS_Bsp-like~SH3~GBS_Bsp-like~SH3 |
| 8 | Lactobacillales | YSIRK_signal~PTZ00449 |
| 8 | Lactobacillales | YSIRK_signal~PRK13108~CshA_fibril_rpt |
| 8 | Bacilli | YSIRK_signal~MSCRAMM_SdrC~SSURE |
| 8 | Staphylococcus | YSIRK_signal~NEAT~NEAT |
| 8 | Staphylococcus xylosus | PTZ00341~YSIRK_signal~Smc |
| 8 | Lactobacillus iners | YSIRK_signal~MSCRAMM_SdrC~SdrG_C_C~MucBP |
| 8 | Streptococcus | YSIRK_signal~PRK10118~pullulan_Gpos |
| 8 | Firmicutes | YSIRK_signal~Peptidase_M26_C~G5 |
| 8 | Staphylococcus | YSIRK_signal~MSCRAMM_SdrC~PTZ00108~Abhydrolase |
| 8 | Streptococcus | YSIRK_signal~PHA03269~pullulan_Gpos |
| 8 | Streptococcus suis | YSIRK_signal~GAGBD~PTZ00121~G5~COG4932 |
| 8 | Streptococcus suis | YSIRK_signal~GAGBD~PRK10263~G5~COG4932 |
| 8 | Streptococcus pneumoniae | YSIRK_signal~PTZ00121~GH20_hexosaminidase |
| 8 | Streptococcus | YSIRK_signal~PTZ00121~LacZ~Big_4~YabE~G5 |
| 8 | Streptococcus pneumoniae | YSIRK_signal~PRK05035~G5~Glutenin_hmw~Collagen~RPT_S_cricet~Collagen~RPT_S_cricet~Herpes_BLLF1~RPT_S_cricet |
| 8 | Streptococcus | YSIRK_signal~DUF1542~PTZ00121~DUF1542 |
| 8 | Streptococcus | MSCRAMM_SdrC~YSIRK_signal~LacZ~Big_4~DUF5401~G5 |
| 8 | Streptococcus | YSIRK_signal~MSCRAMM_SdrC~FctA~Antigen_C~FctA~Antigen_C~FctA~Antigen_C~FctA~Antigen_C~FctA~Antigen_C~FctA~Antigen_C~FctA~Antigen_C~FctA~Antigen_C~FctA |
| 8 | Streptococcus | YSIRK_signal~PRK08119~SSURE |
| 8 | Streptococcus suis | YSIRK_signal~Peptidase_M26_C~PL-6~G5 |
| 8 | Staphylococcus | YSIRK_signal~FTZ~Abhydrolase |
| 8 | Streptococcus agalactiae | MSCRAMM_SdrC~YSIRK_signal~He_PIG~Rib |
| 8 | Staphylococcus pseudintermedius | YSIRK_signal~B~MSCRAMM_SdrC~B~LysM |
| 8 | Streptococcus | YSIRK_signal~MARCKS~LacZ~Big_4~G5 |
| 8 | Streptococcus | YSIRK_signal~rne~Peptidases_S8_S53~PA~fn3_5 |
| 8 | Staphylococcus | MSCRAMM_SdrC~YSIRK_signal~NEAT~IsdB |
| 8 | Lactobacillales | MSCRAMM_SdrC~YSIRK_signal~PHA03247 |
| 8 | Lactobacillus reuteri | YSIRK_signal~PRK10856~Rib |
| 8 | Bacilli | YSIRK_signal~DUF4775 |
| 8 | Staphylococcus | YSIRK_signal~MSCRAMM_SdrC~DUF4887~YncE~MSCRAMM_SdrC~Herpes_LMP1 |
| 8 | Streptococcus suis | IsdB~YSIRK_signal~pullulan_Gpos~AmyAc_family~pullulan_Gpos~G5 |
| 8 | Streptococcus suis | YSIRK_signal~MSCRAMM_SdrC~Peptidases_S8_S53~PA |
| 8 | Streptococcus pneumoniae | YSIRK_signal~MSCRAMM_SdrC~GH18_chitinase-like~F5_F8_type_C~Big_3~CALCOCO1~FIVAR |
| 8 | Streptococcus pneumoniae | YSIRK_signal~PTZ00121~RICH~CW_binding_1~COG5263 |
| 8 | Streptococcus pneumoniae | YSIRK_signal~G5~Collagen~Glutenin_hmw~Collagen~RPT_S_cricet~Herpes_BLLF1~RPT_S_cricet |
| 8 | Streptococcus pneumoniae | YSIRK_signal~DUF4775~G5~Collagen~Glutenin_hmw~RPT_S_cricet~Collagen |
| 8 | Bacteria | YSIRK_signal~MIP-T3~lectin_L-type~MucBP |
| 8 | Lactobacillales | YSIRK_signal~MucBP~PHA03247 |
| 8 | Lactobacillus johnsonii | YSIRK_signal~PRK08581~Peptidase_M26_C |
| 8 | Staphylococcus | YSIRK_signal~MSCRAMM_SdrC~Herpes_BLLF1~PRK12678 |
| 8 | Staphylococcus hominis | YSIRK_signal~MSCRAMM_SdrC~hyperosmo_Ebh~vATP-synt_E |
| 8 | Streptococcus pneumoniae | YSIRK_signal~DUF4775~Collagen~Glutenin_hmw~RPT_S_cricet~Collagen~RPT_S_cricet |
| 8 | Streptococcus | YSIRK_signal~INTAP~pullulan_Gpos |
| 8 | Streptococcus | YSIRK_signal~MSCRAMM_SdrC~Chb~G5 |
| 7 | Streptococcus | MSCRAMM_SdrC~YSIRK_signal~LamG~Sialidase |
| 7 | Streptococcus agalactiae | MSCRAMM_SdrC~YSIRK_signal~Peptidases_S8_S53~PA |
| 7 | Bacilli | MSCRAMM_SdrC~YSIRK_signal~Smc |
| 7 | Staphylococcus | YSIRK_signal~MSCRAMM_SdrC~Aim21~Abhydrolase |
| 7 | Streptococcus pneumoniae | YSIRK_signal~PRK05035~G5~Collagen~Glutenin_hmw~Collagen~RPT_S_cricet~Herpes_BLLF1~RPT_S_cricet |
| 7 | Streptococcus pneumoniae | YSIRK_signal~CDC27~G5~Glutenin_hmw~Collagen~RPT_S_cricet |
| 7 | Lactobacillales | YSIRK_signal~MucBP~PRK15313 |
| 7 | Lactobacillales | YSIRK_signal~MSCRAMM_SdrC~He_PIG~Rib |
| 7 | Streptococcus | YSIRK_signal~Glyco_hyd_65N_2~ATH1 |
| 7 | Staphylococcus aureus | YSIRK_signal~PRK07764~SasC_Mrp_aggreg~DUF1542~PRK05035~DUF1542 |
| 7 | Streptococcus suis | YSIRK_signal~PHA03247~Peptidase_M26_N~Peptidase_M26_C |
| 7 | Streptococcus suis | YSIRK_signal~rne~Peptidase_M26_C~MDR |
| 7 | Staphylococcus | MSCRAMM_SdrC~YSIRK_signal~rne~PTZ00121 |
| 7 | Staphylococcus epidermidis | YSIRK_signal~PHA03169~MSCRAMM_SdrC |
| 7 | Dolosigranulum pigrum | YSIRK_signal~GA~G5~Peptidase_M26_N~Peptidase_M26_C |
| 7 | Streptococcus pneumoniae | YSIRK_signal~PTZ00121~G5~Collagen~RPT_S_cricet~Glutenin_hmw~Collagen~RPT_S_cricet~Herpes_BLLF1~RPT_S_cricet |
| 7 | Streptococcus suis | YSIRK_signal~PRK08026~MucBP |
| 7 | Streptococcus pneumoniae | YSIRK_signal~RICH~DUF390~COG5263 |
| 7 | Bacilli | YSIRK_signal~Mplasa_alph_rch~SasC_Mrp_aggreg~rne~hyperosmo_Ebh~TonB_C |
| 7 | Streptococcus | YSIRK_signal~G5~PRK10263~G5~Peptidase_M26_N~Peptidase_M26_C |
| 7 | Bacilli | MSCRAMM_SdrC~YSIRK_signal~PTZ00449 |
| 7 | Streptococcus suis | YSIRK_signal~GAGBD~rne~G5~COG4932 |
| 7 | Streptococcus | YSIRK_signal~rne~GH43_62_32_68_117_130~PTZ00449~TALPID3 |
| 7 | Streptococcus | YSIRK_signal~hyperosmo_Ebh~DUF1542~PTZ00121~DUF1542 |
| 7 | Lactobacillus | YSIRK_signal~PTZ00341~choice_anch_A |
| 7 | Streptococcus | YSIRK_signal~PTZ00121~hyperosmo_Ebh |
| 7 | Streptococcus suis | YSIRK_signal~235kDa-fam~GA~FIVAR~hyperosmo_Ebh~rne~hyperosmo_Ebh |
| 7 | Bacteria | YSIRK_signal~rne~pullulan_Gpos |
| 7 | Lactobacillales | MSCRAMM_SdrC~YSIRK_signal~MucBP |
| 7 | Staphylococcus capitis | YSIRK_signal~SasC_Mrp_aggreg~DUF1542~PTZ00121~DUF1542~DUF3584~DUF1542~SbcC~DUF1542 |
| 7 | Staphylococcus aureus | YSIRK_signal~PRK07764~SasC_Mrp_aggreg~DUF1542~PTZ00121~PRK05035~DUF1542~PRK08581 |
| 7 | Streptococcus | MSCRAMM_SdrC~YSIRK_signal~COG3942~SH3~GBS_Bsp-like~SH3 |
| 7 | Streptococcus | YSIRK_signal~PRK07003 |
| 7 | Staphylococcus epidermidis | YSIRK_signal~PRK07764~Herpes_BLLF1~PRK12678 |
| 7 | Streptococcus | YSIRK_signal~GH18_chitinase-like~F5_F8_type_C~Big_3 |
| 7 | Streptococcus parauberis | YSIRK_signal~GBS_Bsp-like~Transpeptidase |
| 7 | Streptococcus | YSIRK_signal~Peptidases_S8_S53~PA~fn3_5~FlgD_ig |
| 7 | Lactobacillus | YSIRK_signal~DUF285 |
| 7 | Staphylococcus aureus | YSIRK_signal~B~MDN1~B~LysM |
| 7 | Staphylococcus aureus | YSIRK_signal~Med15~SasC_Mrp_aggreg~DUF1542 |
| 7 | Streptococcus | YSIRK_signal~Chb~rne~G5 |
| 6 | Staphylococcus aureus | YSIRK_signal~PRK07764~SasC_Mrp_aggreg~DUF1542~PRK05035~DUF1542~PTZ00121~DUF1542~PRK08581 |
| 6 | Streptococcus pneumoniae | YSIRK_signal~RICH~Smc~COG5263~CW_binding_1~glucan_65_rpt |
| 6 | Streptococcus pneumoniae | YSIRK_signal~hyperosmo_Ebh~FIVAR~Pneumo_att_G |
| 6 | Streptococcus pneumoniae | YSIRK_signal~rne~hyperosmo_Ebh~PTZ00121~DUF1542 |
| 6 | Streptococcus azizii | YSIRK_signal~Rib_recp_KP_reg~LamG~Sialidase~SGNH_hydrolase~F5_F8_type_C~G5 |
| 6 | Streptococcus azizii | YSIRK_signal~Herpes_BLLF1~Rib~He_PIG |
| 6 | Staphylococcus | YSIRK_signal~MSCRAMM_SdrC~SasC_Mrp_aggreg~DUF1542~PTZ00121~DUF1542~PTZ00121 |
| 6 | Streptococcus oralis | YSIRK_signal~G5~FIVAR~Peptidase_M26_N~Peptidase_M26_C |
| 6 | Staphylococcus | MSCRAMM_SdrD~YSIRK_signal~MG1~PHA03255~MG1 |
| 6 | Staphylococcus | MSCRAMM_SdrC~YSIRK_signal~MSCRAMM_SdrC |
| 6 | Lactobacillales | YSIRK_signal~PTZ00121~Rib |
| 6 | Staphylococcus | YSIRK_signal~MSCRAMM_SdrD~YncE~Herpes_LMP1~IsdH_HarA |
| 6 | Lactobacillus | MSCRAMM_SdrC~YSIRK_signal~TonB |
| 6 | Streptococcus pseudopneumoniae | YSIRK_signal~PRK13108~PTZ00121 |
| 6 | Streptococcus gordonii | YSIRK_signal~G5~rne~G5~FIVAR~Peptidase_M26_N~Peptidase_M26_C |
| 6 | Streptococcus pneumoniae | YSIRK_signal~RICH~PTZ00121~CW_binding_1 |
| 6 | Streptococcus | YSIRK_signal~DUF4775~Tryp_SPc~AfuC~Calx-beta |
| 6 | Streptococcus | YSIRK_signal~Tryp_SPc~AfuC~Calx-beta |
| 6 | Streptococcus | YSIRK_signal~MSCRAMM_SdrC~Peptidases_S8_S53~PA~fn3_5~FlgD_ig~PRK11907 |
| 6 | Lactobacillales | YSIRK_signal~MSCRAMM_SdrC~CshA_fibril_rpt~repeat_SSSPR51 |
| 6 | Streptococcus parasanguinis | MSCRAMM_SdrC~YSIRK_signal~termin_org_DnaJ~Herpes_BLLF1 |
| 6 | Streptococcus | IsdB~YSIRK_signal~MucBP |
| 6 | Streptococcus | YSIRK_signal~Tryp_SPc~AfuC~Calx-beta~flagell_flgL |
| 6 | Streptococcus oralis | YSIRK_signal~Tryp_SPc~AfuC~Calx-beta~DUF1542~PHA03247~G5 |
| 6 | Streptococcus | YSIRK_signal~MSCRAMM_SdrC~Glyco_hyd_65N_2 |
| 6 | Streptococcus thermophilus | YSIRK_signal~Trypan_PARP~PTZ00449 |
| 6 | Streptococcus | YSIRK_signal~G5~zmp_18_rpt~rne~zmp_18_rpt~G5~FIVAR~Peptidase_M26_N~Peptidase_M26_C |
| 6 | Streptococcus | YSIRK_signal~Tryp_SPc~AfuC~Calx-beta~PHA03247~PTZ00449 |
| 6 | Streptococcus | YSIRK_signal~Tryp_SPc~AfuC~Calx-beta~DUF1542~PTZ00449~G5 |
| 6 | Staphylococcus | YSIRK_signal~MSCRAMM_SdrC~MDN1 |
| 6 | Streptococcus suis | YSIRK_signal~GAGBD~PTZ00121~G5 |
| 6 | Streptococcus pneumoniae | YSIRK_signal~G5~Glutenin_hmw~Collagen~RPT_S_cricet |
| 6 | Streptococcus suis | YSIRK_signal~PTZ00121~PRK05035~DUF1542 |
| 6 | Staphylococcus aureus | YSIRK_signal~PRK07764~SasC_Mrp_aggreg~DUF1542~PTZ00121~DUF1542~PRK14949~DMP1 |
| 6 | Staphylococcus aureus | YSIRK_signal~MSCRAMM_SdrC~DUF5401~Abhydrolase |
| 6 | Streptococcus | YSIRK_signal~GH43_62_32_68_117_130~PTZ00449 |
| 6 | Streptococcus pseudopneumoniae | YSIRK_signal~PRK13108~DUF1542 |
| 6 | Streptococcus pyogenes | YSIRK_signal~SH3_and_anchor |
| 6 | Lactobacillales | YSIRK_signal~GA |
| 6 | Firmicutes | YSIRK_signal~Flg_new |
| 6 | Streptococcus suis | YSIRK_signal~GAG_Lyase |
| 6 | Streptococcus dysgalactiae | YSIRK_signal~IgG_binding_B |
| 6 | Streptococcus pneumoniae | YSIRK_signal~GREB1~hyperosmo_Ebh |
| 6 | Staphylococcus hominis | YSIRK_signal~MSCRAMM_SdrC~hyperosmo_Ebh~Smc |
| 6 | Lactobacillales | YSIRK_signal~Peptidase_M26_N~Peptidase_M26_C |
| 6 | Lactobacillus taiwanensis | YSIRK_signal~PRK15313~FIVAR~DUF1542~YhgE~DUF1542~PTZ00121~DUF1542 |
| 6 | Streptococcus canis | YSIRK_signal~Peptidases_S8_S53~PA~fn3_5~FlgD_ig~COG4372~FIVAR |
| 6 | Streptococcus pneumoniae | YSIRK_signal~DUF4775~Collagen~Glutenin_hmw~RPT_S_cricet |
| 6 | Staphylococcus pseudintermedius | YSIRK_signal~MSCRAMM_SdrC~termin_org_DnaJ~Abhydrolase |
| 6 | Staphylococcus simulans | MSCRAMM_SdrC~YSIRK_signal~rne~lectin_L-type~He_PIG~PTZ00121 |
| 6 | Staphylococcus chromogenes | MSCRAMM_SdrC~YSIRK_signal~rne~Rib |
| 6 | Staphylococcus | MSCRAMM_SdrC~YSIRK_signal~COG5651~G5 |
| 6 | Dolosigranulum pigrum | YSIRK_signal~SMC_prok_B~Peptidase_M26_N~Peptidase_M26_C |
| 6 | Staphylococcus xylosus | MSCRAMM_SdrC~YSIRK_signal~PTZ00121~Abhydrolase |
| 6 | Streptococcus | YSIRK_signal~PRK13108~pullulan_Gpos |
| 6 | Lactobacillus iners | YSIRK_signal~IsdB~hyperosmo_Ebh~Rib~hyperosmo_Ebh |
| 6 | Lactobacillus | YSIRK_signal~MSCRAMM_SdrC~hyperosmo_Ebh~Rib |
| 6 | Streptococcus agalactiae | MSCRAMM_SdrC~YSIRK_signal~He_PIG |
| 6 | Streptococcus suis | MSCRAMM_SdrC~YSIRK_signal~GAGBD~G5~PTZ00144~G5 |
| 6 | Streptococcus suis | YSIRK_signal~rne~GAGBD~G5~PRK13108~G5~COG4932 |
| 6 | Streptococcus thermophilus | YSIRK_signal~rne~PTZ00449 |
| 6 | Streptococcus pneumoniae | YSIRK_signal~PRK14949~hyperosmo_Ebh~DUF1542 |
| 6 | Lactobacillales | YSIRK_signal~G5~Peptidase_M26_N |
| 6 | Streptococcus pneumoniae | YSIRK_signal~PRK14949~MARCKS~hyperosmo_Ebh~Periplasmic_Binding_Protein_Type_1~hyperosmo_Ebh~DUF1542 |
| 6 | Streptococcus pneumoniae | YSIRK_signal~hyperosmo_Ebh~DUF1542~PDCD7 |
| 6 | Streptococcus pneumoniae | YSIRK_signal~RICH~SMC_prok_B~COG5263 |
| 6 | Bacilli | YSIRK_signal~PRK14949 |
| 6 | Streptococcus suis | YSIRK_signal~rne~Peptidase_M26_C~Rib |
| 6 | Bacilli | YSIRK_signal~PRK10856 |
| 5 | Lactobacillus acidophilus | MSCRAMM_SdrD~YSIRK_signal~MISS~PTZ00449 |
| 5 | Staphylococcus | YSIRK_signal~MSCRAMM_SdrC~SasC_Mrp_aggreg~DUF1542~SMC_prok_B |
| 5 | Streptococcus suis | YSIRK_signal~GAGBD~PLN03209~G5~COG4932 |
| 5 | Streptococcus suis | YSIRK_signal~rne~PTZ00121 |
| 5 | Streptococcus agalactiae | YSIRK_signal~SucB_Actino~PRK10819 |
| 5 | Staphylococcus epidermidis | YSIRK_signal~PRK07764~PHA03247~PRK12678 |
| 5 | Streptococcus agalactiae | YSIRK_signal~SucB_Actino~PRK11633 |
| 5 | Streptococcus pneumoniae | YSIRK_signal~PTZ00121~G5~Glutenin_hmw~RPT_S_cricet~Herpes_BLLF1~RPT_S_cricet |
| 5 | Streptococcus | YSIRK_signal~PRK07994~CshA_fibril_rpt |
| 5 | Streptococcus pneumoniae | YSIRK_signal~DUF4775~G5~Collagen~Glutenin_hmw~Collagen~RPT_S_cricet~Collagen~RPT_S_cricet~Herpes_BLLF1~RPT_S_cricet |
| 5 | Staphylococcus aureus | YSIRK_signal~SasC_Mrp_aggreg~DUF1542~PTZ00121~DUF1542~PRK08581~DMP1 |
| 5 | Streptococcus | YSIRK_signal~GH18_chitinase-like~F5_F8_type_C~Big_3~PTZ00449 |
| 5 | Staphylococcus chromogenes | MSCRAMM_SdrC~YSIRK_signal~rne~Rib~Herpes_BLLF1 |
| 5 | Streptococcus suis | YSIRK_signal~SH3~Peptidase_M15~SH3 |
| 5 | Streptococcus suis | YSIRK_signal~rne~Peptidase_M26_C~NosD_copper_fam |
| 5 | Staphylococcus | YSIRK_signal~MSCRAMM_SdrC~PRK00708~Abhydrolase |
| 5 | Staphylococcus agnetis | YSIRK_signal~DUF3583~ftsN~Abhydrolase |
| 5 | Staphylococcus cohnii | YSIRK_signal~MSCRAMM_SdrC~MucBP~PHA03247 |
| 5 | Bacilli | YSIRK_signal~rne~MSCRAMM_SdrC |
| 5 | Staphylococcus sciuri | YSIRK_signal~MSCRAMM_SdrD~NEAT |
| 5 | Streptococcus pneumoniae | YSIRK_signal~IsdB~hyperosmo_Ebh~DUF1542~PRK03918 |
| 5 | Staphylococcus | YSIRK_signal~Filo_glycop~DUF1542~SMC_N~DUF1542 |
| 5 | Bacilli | YSIRK_signal~He_PIG |
| 5 | Dolosigranulum pigrum | YSIRK_signal~PTZ00121~PRK10819 |
| 5 | Streptococcus pneumoniae | YSIRK_signal~PTZ00121~G5~Collagen~RPT_S_cricet~Glutenin_hmw~Collagen~RPT_S_cricet~Cornifin |
| 5 | Streptococcus pneumoniae | YSIRK_signal~RICH~DUF5401~RICH~COG5263 |
| 5 | Lactobacillus reuteri | YSIRK_signal~COG4934~FIVAR~DUF1542~PRK05035~DUF1542~PTZ00121~DUF1542 |
| 5 | Lactobacillus reuteri | YSIRK_signal~PRK09752~FIVAR~DUF1542~PTZ00121~DUF1542 |
| 5 | Staphylococcus aureus | YSIRK_signal~PRK07764~SasC_Mrp_aggreg~DUF1542~PTZ00121~PRK08581 |
| 5 | Staphylococcus aureus | YSIRK_signal~PRK07764~SasC_Mrp_aggreg~DUF1542~PTZ00121~SbcC~DUF1542~PRK08581 |
| 5 | Streptococcus suis | YSIRK_signal~rne~GAGBD~G5~PTZ00144~G5~COG4932 |
| 5 | Staphylococcus | YSIRK_signal~MSCRAMM_SdrC~MG1 |
| 5 | Staphylococcus haemolyticus | YSIRK_signal~MSCRAMM_SdrD~MG1~Herpes_BLLF1 |
| 5 | Streptococcus | YSIRK_signal~rne~pullulan_Gpos~AmyAc_family~pullulan_Gpos |
| 5 | Streptococcus | YSIRK_signal~Neisseria_TspB~Trypan_PARP |
| 5 | Streptococcus pneumoniae | YSIRK_signal~DUF4775~G5~Collagen~RPT_S_cricet~Collagen~RPT_S_cricet |
| 5 | Streptococcus | YSIRK_signal~Metaviral_G~pullulan_Gpos |
| 5 | Streptococcus salivarius | YSIRK_signal~MSCRAMM_SdrC~FctA~Antigen_C~FctA~Antigen_C~FctA~Antigen_C~FctA~Antigen_C~FctA~Antigen_C~FctA~Antigen_C~FctA~Antigen_C~FctA~Antigen_C~FctA~Antigen_C~FctA |
| 5 | Streptococcus | YSIRK_signal~MSCRAMM_SdrC~FctA~Antigen_C~FctA~Antigen_C~FctA~Antigen_C~FctA~Antigen_C~FctA~Antigen_C~FctA~Antigen_C~FctA~Antigen_C~FctA~Antigen_C~FctA~Antigen_C~FctA~Antigen_C~FctA |
| 5 | Streptococcus pneumoniae | YSIRK_signal~G5~Glutenin_hmw~RPT_S_cricet~2A1904~Glutenin_hmw~Collagen~RPT_S_cricet |
| 5 | Bacilli | YSIRK_signal~MSCRAMM_SdrC~Peptidases_S8_S53~PA~fn3_5~FlgD_ig |
| 5 | Streptococcus pneumoniae | YSIRK_signal~RICH~SMC_prok_A~COG5263~CW_binding_1 |
| 5 | Streptococcus pneumoniae | YSIRK_signal~PTZ00121~G5~Collagen~RPT_S_cricet |
| 5 | Staphylococcus warneri | YSIRK_signal~Mplasa_alph_rch~SasC_Mrp_aggreg~rne~hyperosmo_Ebh |
| 5 | Bacilli | YSIRK_signal~MSCRAMM_SdrC~rne |
| 5 | Staphylococcus | YSIRK_signal~B~PRK13914~LysM |
| 5 | Streptococcus suis | YSIRK_signal~235kDa-fam~FIVAR~hyperosmo_Ebh~rne~hyperosmo_Ebh |
| 5 | Streptococcus gordonii | YSIRK_signal~YabE~G5~zmp_18_rpt~NESP55~zmp_18_rpt~G5~FIVAR~Peptidase_M26_N~Peptidase_M26_C |
| 5 | Streptococcus | MSCRAMM_SdrC~YSIRK_signal~Peptidases_S8_S53~PA~fn3_5~FlgD_ig~PRK11907 |
| 5 | Streptococcus pneumoniae | YSIRK_signal~DUF4775~Collagen~RPT_S_cricet |
| 5 | Staphylococcus agnetis | YSIRK_signal~FliK~Abhydrolase |
| 5 | Streptococcus mitis | YSIRK_signal~hyperosmo_Ebh~DUF1542~PTZ00121 |
| 5 | Streptococcus mitis | YSIRK_signal~G5~PHA03418~G5~Peptidase_M26_N~Peptidase_M26_C |
| 5 | Streptococcus | YSIRK_signal~DUF1542~PTZ00121 |
| 5 | Streptococcus | YSIRK_signal~MSCRAMM_SdrC~F5_F8_type_C~Glyco_hydro_20b~GH20_hexosaminidase~F5_F8_type_C~Glyco_hydro_20b~NAGidase~Glyco_hydro_106~F5_F8_type_C~G5 |
| 5 | Streptococcus | YSIRK_signal~Treacle~pullulan_Gpos |
| 5 | Streptococcus | YSIRK_signal~PRK13335~pullulan_Gpos |
| 5 | Streptococcus pneumoniae | YSIRK_signal~PRK07994~hyperosmo_Ebh~DUF1542 |
| 5 | Lactobacillus gasseri | YSIRK_signal~MSCRAMM_SdrC~Herpes_BLLF1~Rib |
| 5 | Staphylococcus aureus | YSIRK_signal~Med15~SasC_Mrp_aggreg~DUF1542~PRK05035~DUF1542~PTZ00108 |
| 5 | Staphylococcus epidermidis | YSIRK_signal~MSCRAMM_SdrC~Herpes_BLLF1~2A1904 |
| 5 | Staphylococcus aureus | YSIRK_signal~Glutenin_hmw~SasC_Mrp_aggreg~DUF1542~PTZ00121~DUF1542~PRK08581 |
| 5 | Staphylococcus aureus | YSIRK_signal~SasC_Mrp_aggreg~PTZ00121~DUF1542~PRK08581~DMP1 |
| 5 | Streptococcus pneumoniae | YSIRK_signal~PRK05035~G5~Glutenin_hmw~Collagen~RPT_S_cricet~Collagen~RPT_S_cricet |
| 5 | Streptococcus pneumoniae | YSIRK_signal~RICH~tolA_full~COG5263 |
| 5 | Streptococcus | YSIRK_signal~DUF4775~Glyco_hydro_20b~NAGidase~F5_F8_type_C |
| 5 | Streptococcus | YSIRK_signal~Thiol_cytolysin~Thiol_cytolys_C |
| 5 | Firmicutes | YSIRK_signal~PTZ00341 |
| 5 | Staphylococcus aureus | YSIRK_signal~B~PRK04195 |
| 5 | Streptococcus agalactiae | MSCRAMM_SdrC~YSIRK_signal~G5~Collagen~RPT_S_cricet~PTZ00449 |
| 5 | Streptococcus suis | YSIRK_signal~PRK10263~PTZ00449~G5~PRK10263~G5~Peptidase_M26_N~ATP-synt_DE~Peptidase_M26_C |
| 5 | Streptococcus pneumoniae | YSIRK_signal~CDC27~G5~Collagen~Glutenin_hmw~RPT_S_cricet |
| 5 | Lactobacillus | YSIRK_signal~Abhydrolase~PldB~GA~FIVAR~PHA03247 |
| 5 | Streptococcus | YSIRK_signal~Tryp_SPc~DUF4887~GH_101_like~Glyco_hyd_101C~F5_F8_type_C~pullulan_Gpos |
| 5 | Staphylococcus | YSIRK_signal~MSCRAMM_SdrC~PRK00247~Abhydrolase |
| 5 | Staphylococcus | YSIRK_signal~PRK07764~MDN1 |
| 5 | Streptococcus | YSIRK_signal~PTZ00121~GAGBD~RICH~PTZ00449 |
| 5 | Streptococcus | YSIRK_signal~termin_org_DnaJ~G5~Peptidase_M26_N~Peptidase_M26_C |
| 5 | Streptococcus | IsdB~YSIRK_signal~HlyD~CshA_fibril_rpt |
| 5 | Bacilli | YSIRK_signal~hyperosmo_Ebh~Rib~hyperosmo_Ebh~Rib |
| 5 | Streptococcus pneumoniae | YSIRK_signal~hyperosmo_Ebh~Periplasmic_Binding_Protein_Type_1~hyperosmo_Ebh |
| 5 | Streptococcus | YSIRK_signal~FctA~Antigen_C~FctA~Antigen_C~FctA~Antigen_C~FctA~Antigen_C~FctA~Antigen_C~FctA |
| 5 | Streptococcus | YSIRK_signal~SasC_Mrp_aggreg~Rib |
| 5 | Streptococcus pneumoniae | YSIRK_signal~DUF4775~G5~Glutenin_hmw~RPT_S_cricet~Glutenin_hmw~Collagen~RPT_S_cricet |
| 5 | Staphylococcus aureus | YSIRK_signal~MSCRAMM_SdrC~PRK10905~SasC_Mrp_aggreg~DUF1542 |
| 5 | Firmicutes | YSIRK_signal~Trypan_PARP |
| 4 | Streptococcus pneumoniae | YSIRK_signal~PTZ00121~G5~Glutenin_hmw~Collagen~Glutenin_hmw~Collagen~RPT_S_cricet |
| 4 | Streptococcus oralis | YSIRK_signal~COG5665~MucBP |
| 4 | Streptococcus | YSIRK_signal~SH3~GBS_Bsp-like~SH3~GBS_Bsp-like~Peptidase_M15~SH3 |
| 4 | Streptococcus | MSCRAMM_SdrC~YSIRK_signal~LacZ~Big_4~rad2~G5 |
| 4 | Lactobacillus reuteri | YSIRK_signal~FAP~PHA03291 |
| 4 | Streptococcus pneumoniae | YSIRK_signal~RICH~COG5263~CW_binding_1~glucan_65_rpt |
| 4 | Bacilli | YSIRK_signal~PRK05035 |
| 4 | Streptococcus | YSIRK_signal~PTZ00121~LacZ~Big_4~YabE~G5~COG5263 |
| 4 | Streptococcus mitis | YSIRK_signal~Herpes_TAF50~G5~Peptidase_M26_N~Peptidase_M26_C |
| 4 | Bacilli | MSCRAMM_SdrC~YSIRK_signal~Herpes_BLLF1 |
| 4 | Staphylococcus | YSIRK_signal~PTZ00395 |
| 4 | Streptococcus pneumoniae | YSIRK_signal~hyperosmo_Ebh~DUF1542~SbcC |
| 4 | Streptococcus pneumoniae | YSIRK_signal~COG1340~hyperosmo_Ebh~Rib~hyperosmo_Ebh~Rib |
| 4 | Streptococcus pneumoniae | YSIRK_signal~SCP-1~COG1579~GAGBD~COG5263 |
| 4 | Staphylococcus | YSIRK_signal~MSCRAMM_SdrC~SasC_Mrp_aggreg~DUF1542~PRK05035~PTZ00121 |
| 4 | Streptococcus | YSIRK_signal~MSCRAMM_SdrD~hyperosmo_Ebh~DUF1542 |
| 4 | Streptococcus sanguinis | YSIRK_signal~PRK12373~pullulan_Gpos |
| 4 | Streptococcus | YSIRK_signal~SMC_prok_B~CshA_fibril_rpt |
| 4 | Staphylococcus aureus | YSIRK_signal~B~InfB |
| 4 | Streptococcus pneumoniae | YSIRK_signal~rne~hyperosmo_Ebh~DUF1542~PDCD7 |
| 4 | Streptococcus pneumoniae | YSIRK_signal~PRK14949~hyperosmo_Ebh |
| 4 | Streptococcus pneumoniae | YSIRK_signal~hyperosmo_Ebh~PHA03255~Smc |
| 4 | Lactobacillus gasseri | MSCRAMM_SdrC~YSIRK_signal~Amelogenin~pullulan_Gpos |
| 4 | Streptococcus intermedius | YSIRK_signal~Peptidases_S8_S53~PA~fn3_5~FlgD_ig~FIVAR |
| 4 | Streptococcus suis | YSIRK_signal~rne~MucBP~Cornifin~repeat_SSSPR51 |
| 4 | Streptococcus | IsdB~YSIRK_signal~CshA_fibril_rpt |
| 4 | Streptococcus | YSIRK_signal~rplD~pullulan_Gpos |
| 4 | Streptococcus pneumoniae | YSIRK_signal~PTZ00121~Trypan_PARP~Peptidase_M26_N |
| 4 | Streptococcus thermophilus | YSIRK_signal~Trypan_PARP~MucBP~PTZ00449 |
| 4 | Streptococcus cristatus | MSCRAMM_SdrC~YSIRK_signal~LacZ~Big_4~G5~pullulan_Gpos |
| 4 | Streptococcus | YSIRK_signal~SMC_prok_B~CshA_fibril_rpt~repeat_SSSPR51 |
| 4 | Staphylococcus | YSIRK_signal~B~Trypan_PARP~LysM |
| 4 | Streptococcus pneumoniae | YSIRK_signal~PRK05035~Collagen~Glutenin_hmw~Collagen~RPT_S_cricet |
| 4 | Streptococcus pneumoniae | YSIRK_signal~DUF4775~G5~RPT_S_cricet~Collagen |
| 4 | Streptococcus pneumoniae | YSIRK_signal~hyperosmo_Ebh~Periplasmic_Binding_Protein_Type_1~hyperosmo_Ebh~PTZ00121 |
| 4 | Lactobacillales | YSIRK_signal~Rib~hyperosmo_Ebh |
| 4 | Streptococcus agalactiae | YSIRK_signal~hyperosmo_Ebh~Rib~He_PIG |
| 4 | Gemella haemolysans | MSCRAMM_SdrC~YSIRK_signal~SSURE |
| 4 | Streptococcus suis | YSIRK_signal~Mac-1~PHA03247 |
| 4 | Streptococcus suis | YSIRK_signal~GAGBD~PTZ00121~COG4932 |
| 4 | Staphylococcus epidermidis | YSIRK_signal~MSCRAMM_SdrC~PRK07764~MDN1 |
| 4 | Lactobacillus | YSIRK_signal~MSCRAMM_SdrC~FIVAR~Rib |
| 4 | Streptococcus suis | YSIRK_signal~MSCRAMM_SdrC~MucBP~Cornifin~repeat_SSSPR51 |
| 4 | Streptococcus | YSIRK_signal~zmp_18_rpt~G5~FIVAR~Peptidase_M26_N~Peptidase_M26_C |
| 4 | Streptococcus pneumoniae | YSIRK_signal~DUF4775~G5~Collagen~Glutenin_hmw~Collagen~RPT_S_cricet |
| 4 | Streptococcus suis | YSIRK_signal~predic_Ig_block~PHA03247~Peptidase_M26_N~Peptidase_M26_C |
| 4 | Streptococcus | YSIRK_signal~COG3942~SH3~GBS_Bsp-like~SH3 |
| 4 | Streptococcus mitis | YSIRK_signal~DUF390~hyperosmo_Ebh~Rib~repeat_SSSPR51 |
| 4 | Streptococcus | YSIRK_signal~DUF4775~Tryp_SPc~AfuC~Calx-beta~DUF1542 |
| 4 | Streptococcus | YSIRK_signal~MSCRAMM_SdrC~CBM6-CBM35-CBM36_like~beta_helix_1~Herpes_BLLF1 |
| 4 | Streptococcus | YSIRK_signal~MSCRAMM_SdrC~LamG~Sialidase~2A1904 |
| 4 | Staphylococcus | YSIRK_signal~MSCRAMM_SdrC~hyperosmo_Ebh~DUF4407 |
| 4 | Streptococcus | YSIRK_signal~PTZ00121~IG~GAGBD~RICH~PTZ00449 |
| 4 | Streptococcus | YSIRK_signal~PRK13914~pullulan_Gpos |
| 4 | Streptococcus suis | YSIRK_signal~PTZ00121~G5~YabE~G5~PRK03427~Peptidase_M26_N~Peptidase_M26_C |
| 4 | Streptococcus | YSIRK_signal~PRK08691~G5~Peptidase_M26_N~Peptidase_M26_C |
| 4 | Streptococcus pneumoniae | YSIRK_signal~RICH~PTZ00121~COG5263~glucan_65_rpt~CW_binding_1 |
| 4 | Streptococcus pneumoniae | YSIRK_signal~RICH~PTZ00121~RICH~APG6 |
| 4 | Streptococcus pneumoniae | YSIRK_signal~MARCKS~hyperosmo_Ebh |
| 4 | Streptococcus pneumoniae | YSIRK_signal~RICH~PTZ00121~RICH~APG6~CW_binding_1 |
| 4 | Streptococcus pneumoniae | YSIRK_signal~rne~hyperosmo_Ebh~DUF1542 |
| 4 | Streptococcus suis | YSIRK_signal~PTZ00341~G5~YabE~G5~Peptidase_M26_N~Peptidase_M26_C |
| 4 | Staphylococcus condimenti | YSIRK_signal~PRK08581~MSCRAMM_SdrD |
| 4 | Streptococcus | YSIRK_signal~MSCRAMM_SdrC~FctA~Antigen_C~FctA~Antigen_C~FctA~Antigen_C~FctA~Antigen_C~FctA~Antigen_C~FctA~Antigen_C~FctA~Antigen_C~FctA~Antigen_C~FctA~Antigen_C~FctA~Antigen_C~FctA~Antigen_C~FctA~Antigen_C~FctA |
| 4 | Streptococcus suis | YSIRK_signal~G5~rne~Herpes_BLLF1~G5~Peptidase_M26_N~Peptidase_M26_C |
| 4 | Lactobacillus agilis | YSIRK_signal~SerAla_Lrha_rpt~MucBP |
| 4 | Lactobacillus johnsonii | YSIRK_signal~FIVAR~DUF1542~MDN1~PTZ00121~DUF1542 |
| 4 | Lactobacillus reuteri | YSIRK_signal~MISS |
| 4 | Lactobacillus reuteri | YSIRK_signal~COG4934~FIVAR~DUF1542~PTZ00121~DUF1542 |
| 4 | Lactobacillus | YSIRK_signal~FIVAR~DUF1542~PTZ00121~DUF1542 |
| 4 | Streptococcus salivarius | YSIRK_signal~MSCRAMM_SdrC~FctA~Antigen_C~FctA~Antigen_C~FctA~Antigen_C~FctA~Antigen_C |
| 4 | Streptococcus suis | YSIRK_signal~GAGBD~PTZ00121 |
| 4 | Streptococcus suis | YSIRK_signal~rne~LRR_5~Abhydrolase~Flg_new~G5 |
| 4 | Streptococcus suis | YSIRK_signal~rne~pullulan_Gpos~AmyAc_family~pullulan_Gpos~G5 |
| 4 | Streptococcus suis | YSIRK_signal~MSCRAMM_SdrC~Peptidase_M26_C~G5 |
| 4 | Streptococcus sobrinus | YSIRK_signal~MSCRAMM_SdrC~PTZ00121~GbpC |
| 4 | Streptococcus dysgalactiae | YSIRK_signal~Smc~PLN03237 |
| 4 | Lactobacillales | YSIRK_signal~BASP1 |
| 4 | Lactobacillus acidophilus | YSIRK_signal~MSCRAMM_SdrC~GGN |
| 4 | Streptococcus sobrinus | YSIRK_signal~MSCRAMM_SdrC~PRK05035~PTZ00121~GbpC |
| 4 | Streptococcus | YSIRK_signal~rne~MucBP |
| 4 | Streptococcus | YSIRK_signal~NtpH~SSURE |
| 4 | Staphylococcus aureus | YSIRK_signal~SWI-SNF_Ssr4~SasC_Mrp_aggreg~DUF1542~PTZ00121~DUF1542~PRK08581 |
| 4 | Staphylococcus aureus | YSIRK_signal~PTZ00121~lectin_L-type~G5~SasG_E |
| 4 | Lactobacillales | YSIRK_signal~choice_anch_A |
| 4 | Streptococcus | YSIRK_signal~GH18_chitinase-like |
| 4 | Staphylococcus | YSIRK_signal~DMP1~Pneumo_att_G~Abhydrolase |
| 4 | Streptococcus | YSIRK_signal~Peptidases_S8_S53~PA |
| 4 | Streptococcus pneumoniae | YSIRK_signal~hyperosmo_Ebh~Periplasmic_Binding_Protein_Type_1 |
| 4 | Lactobacillus animalis | YSIRK_signal~AIF-MLS~NESP55~hyperosmo_Ebh~Rib |
| 4 | Staphylococcus aureus | YSIRK_signal~PRK07764~SasC_Mrp_aggreg~DUF1542~PTZ00121~DUF1542~DMP1 |
| 4 | Streptococcus | MSCRAMM_SdrC~YSIRK_signal~LacZ~Big_4 |
| 4 | Lactobacillales | YSIRK_signal~FIVAR |
| 4 | Lactobacillales | YSIRK_signal~G5~Glutenin_hmw~Collagen~RPT_S_cricet~Cornifin |
| 4 | Aerococcus urinae | YSIRK_signal~G5~PHA03378 |
| 4 | Dolosigranulum pigrum | YSIRK_signal~MSCRAMM_SdrC~LamG~Sialidase~Trypan_PARP |
| 4 | Dolosigranulum pigrum | YSIRK_signal~GA~PTZ00121 |
| 4 | Staphylococcus aureus | YSIRK_signal~DUF5401~MSCRAMM_SdrC |
| 4 | Staphylococcus arlettae | MSCRAMM_SdrC~YSIRK_signal~PRK08581~Abhydrolase |
| 4 | Staphylococcus chromogenes | MSCRAMM_SdrC~YSIRK_signal~DUF4775~Rib |
| 4 | Staphylococcus cohnii | YSIRK_signal~MSCRAMM_SdrC~SMC_prok_B |
| 4 | Streptococcus suis | YSIRK_signal~DUF4775~G5~YabE~G5~Peptidase_M26_N~Peptidase_M26_C |
| 4 | Finegoldia magna | YSIRK_signal~PTZ00121~PTZ00449 |
| 4 | Streptococcus vestibularis | YSIRK_signal~PRK11907~Rib~SH3~GBS_Bsp-like~Peptidase_M15~SH3 |
| 4 | Streptococcus | YSIRK_signal~PRK12495~CshA_fibril_rpt |
| 4 | Lactobacillus | MSCRAMM_SdrC~YSIRK_signal~DUF285 |
| 4 | Streptococcus | YSIRK_signal~G5~zmp_18_rpt~G5 |
| 4 | Streptococcus parasanguinis | YSIRK_signal~GH18_chitinase-like~F5_F8_type_C~Big_3~FIVAR~Amelogenin |
| 4 | Streptococcus suis | MSCRAMM_SdrC~YSIRK_signal~GAGBD~G5~PTZ00144~G5~COG4932 |
| 3 | Lactobacillales | YSIRK_signal~MSCRAMM_SdrC~PHA03247 |
| 3 | Streptococcus | MSCRAMM_SdrC~YSIRK_signal~Thiol_cytolysin~Thiol_cytolys_C |
| 3 | Streptococcus suis | MSCRAMM_SdrD~YSIRK_signal~GH18_chitinase-like~F5_F8_type_C~Big_3~TBPIP~FIVAR~G5 |
| 3 | Streptococcus suis | YSIRK_signal~Mac-1~DUF4775~rne |
| 3 | Bacilli | YSIRK_signal~kgd |
| 3 | Staphylococcus | YSIRK_signal~NEAT~NEAT~PTZ00121 |
| 3 | Streptococcus gallolyticus | YSIRK_signal~rne~Peptidases_S8_S53~PA~fn3_5~FlgD_ig~TIGR02680~FIVAR |
| 3 | Staphylococcus | YSIRK_signal~MSCRAMM_SdrC~SasC_Mrp_aggreg~PTZ00121~DUF1542 |
| 3 | Staphylococcus | YSIRK_signal~DUF4813~SasC_Mrp_aggreg~DUF1542~PTZ00121~DUF1542~PRK05035~DUF1542~PHA03255~PRK08581 |
| 3 | Streptococcus suis | YSIRK_signal~Mac-1~rne~PHA03247 |
| 3 | Staphylococcus aureus | YSIRK_signal~SasC_Mrp_aggreg~DUF1542~PRK05035~DUF1542~DMP1 |
| 3 | Streptococcus suis | YSIRK_signal~predic_Ig_block~PHA03247~Peptidase_M26_N~Hia~Peptidase_M26_C |
| 3 | Streptococcus | YSIRK_signal~GH18_chitinase-like~F5_F8_type_C~Big_3~Amelogenin |
| 3 | Streptococcus | YSIRK_signal~PTZ00121~GAGBD |
| 3 | Streptococcus | YSIRK_signal~PRK10856~Chb~G5 |
| 3 | Streptococcus parasanguinis | YSIRK_signal~GH18_chitinase-like~F5_F8_type_C~Big_3~Amelogenin~PHA03247 |
| 3 | Streptococcus pneumoniae | YSIRK_signal~ATP-synt_B~LacZ~Big_4~mycoplas_M_dom~YabE~G5 |
| 3 | Bacilli | YSIRK_signal~PRK05035~PTZ00449~MucBP~Amelogenin |
| 3 | Streptococcus equi | YSIRK_signal~pullulan_Gpos~LPXTG_double |
| 3 | Staphylococcus cohnii | YSIRK_signal~MDN1~SMC_prok_B |
| 3 | Staphylococcus | YSIRK_signal~MARCKS~MSCRAMM_SdrC |
| 3 | Staphylococcus hominis | YSIRK_signal~MSCRAMM_SdrC~hyperosmo_Ebh~DUF4407~Smc |
| 3 | Staphylococcus chromogenes | MSCRAMM_SdrC~YSIRK_signal~rne~Rib~PRK15316 |
| 3 | Staphylococcus | MSCRAMM_SdrC~YSIRK_signal~NEAT~NEAT~IsdB |
| 3 | Lactobacillus gasseri | YSIRK_signal~MSCRAMM_SdrC~PTZ00108~PTZ00449 |
| 3 | Streptococcus | YSIRK_signal~PHA03247~MucBP |
| 3 | Streptococcus suis | MSCRAMM_SdrC~YSIRK_signal~DUF1542~Peptidase_M26_N~Peptidase_M26_C |
| 3 | Streptococcus suis | YSIRK_signal~Mac-1~PRK14949~rne |
| 3 | Streptococcus suis | YSIRK_signal~Mac-1~rne~omega_3_PfaA |
| 3 | Streptococcus suis | YSIRK_signal~PTZ00441~G5~CAP |
| 3 | Dolosigranulum pigrum | MSCRAMM_SdrC~YSIRK_signal~Glyco_hydro_20b~NAGidase~F5_F8_type_C~Glyco_hydro_20b~GH20_hexosaminidase~F5_F8_type_C |
| 3 | Staphylococcus aureus | YSIRK_signal~SasC_Mrp_aggreg~DUF1542~PTZ00121~PRK05035~DUF1542~PRK08581 |
| 3 | Staphylococcus agnetis | YSIRK_signal~choice_anch_A~PTZ00449 |
| 3 | Staphylococcus | YSIRK_signal~rne~MSCRAMM_SdrC~SdrG_C_C |
| 3 | Lactobacillus reuteri | YSIRK_signal~MSCRAMM_SdrC~FIVAR |
| 3 | Streptococcus suis | YSIRK_signal~Mac-1~termin_org_DnaJ~rne |
| 3 | Streptococcus suis | YSIRK_signal~rne~GAGBD~G5~DedD~G5~COG4932 |
| 3 | Streptococcus suis | YSIRK_signal~PRK10263~PRK14960~G5~rne~Peptidase_M26_N~Peptidase_M26_C |
| 3 | Streptococcus suis | MSCRAMM_SdrC~YSIRK_signal~COG3942~SH3 |
| 3 | Staphylococcus xylosus | PTZ00341~YSIRK_signal~SMC_N~Smc |
| 3 | Staphylococcus xylosus | PTZ00341~YSIRK_signal |
| 3 | Staphylococcus | YSIRK_signal~MSCRAMM_SdrD~SdrD_B~MSCRAMM_SdrD |
| 3 | Staphylococcus capitis | YSIRK_signal~PRK07764~PHA03247~Neisseria_TspB |
| 3 | Staphylococcus capitis | YSIRK_signal~PHA03247~Neisseria_TspB |
| 3 | Lactobacillus | YSIRK_signal~PRK11281~GbpC~PTZ00449 |
| 3 | Lactobacillales | YSIRK_signal~PRK05035~Rib |
| 3 | Staphylococcus | YSIRK_signal~Mplasa_alph_rch~SasC_Mrp_aggreg |
| 3 | Streptococcus pneumoniae | YSIRK_signal~PTZ00121~RICH~PTZ00449~CW_binding_1 |
| 3 | Streptococcus mitis | MSCRAMM_SdrC~YSIRK_signal~LamG~Sialidase~LGIC_ECD~G5 |
| 3 | Streptococcus suis | YSIRK_signal~rne~GAGBD~G5 |
| 3 | Streptococcus oralis | YSIRK_signal~Tryp_SPc~AfuC~Calx-beta~NESP55~PTZ00449~G5 |
| 3 | Staphylococcus aureus | YSIRK_signal~MSCRAMM_SdrC~SasC_Mrp_aggreg~DUF1542~sbcc~DUF1542 |
| 3 | Dolosigranulum pigrum | YSIRK_signal~CobT~LacZ |
| 3 | Aerococcus urinae | YSIRK_signal~PRK13108~G5~Glutenin_hmw~Collagen~RPT_S_cricet~Cornifin |
| 3 | Aerococcus urinae | YSIRK_signal~PRK07003~SdrD_B~Collagen~Glutenin_hmw~RPT_S_cricet~Glutenin_hmw~Collagen~Glutenin_hmw~2A1904~RPT_S_cricet~PTZ00449 |
| 3 | Aerococcus urinae | YSIRK_signal~G5~Glutenin_hmw~Collagen~2A1904~RPT_S_cricet~Cornifin |
| 3 | Aerococcus urinae | YSIRK_signal~PTZ00121~Rib~hyperosmo_Ebh |
| 3 | Bacilli | YSIRK_signal~MSCRAMM_SdrC~DUF5011 |
| 3 | Streptococcus | YSIRK_signal~CYK3~PTZ00121 |
| 3 | Streptococcus | MSCRAMM_SdrC~YSIRK_signal~Sialidase |
| 3 | Aerococcus | YSIRK_signal~G5~Glutenin_hmw~Collagen~2A1904 |
| 3 | Streptococcus | Endomucin~YSIRK_signal~pullulan_Gpos |
| 3 | Streptococcus oralis | IsdB~YSIRK_signal~DUF1542 |
| 3 | Lactobacillus | YSIRK_signal~CBP_CCPA |
| 3 | Streptococcus | YSIRK_signal~PTZ00449~PHA03247~PTZ00449 |
| 3 | Streptococcus suis | YSIRK_signal~DUF4775~G5~Peptidase_M26_N~Peptidase_M26_C |
| 3 | Streptococcus oralis | YSIRK_signal~PRK11633~F5_F8_type_C~Glyco_hydro_20b~GH20_hexosaminidase~F5_F8_type_C~Glyco_hydro_20b~NAGidase~Glyco_hydro_106~F5_F8_type_C |
| 3 | Lactobacillus iners | MSCRAMM_SdrC~YSIRK_signal~EmrA |
| 3 | Firmicutes | MSCRAMM_SdrC~YSIRK_signal~Flg_new |
| 3 | Streptococcus | YSIRK_signal~SH3~GBS_Bsp-like~SH3~GBS_Bsp-like~SH3 |
| 3 | Streptococcus pneumoniae | YSIRK_signal~RICH~DNA_S_dndD |
| 3 | Bacilli | YSIRK_signal~Macoilin~SasC_Mrp_aggreg |
| 3 | Streptococcus pneumoniae | YSIRK_signal~CDC27~Glutenin_hmw~Collagen~RPT_S_cricet~Cornifin |
| 3 | Streptococcus pneumoniae | YSIRK_signal~PTZ00441 |
| 3 | Streptococcus pyogenes | YSIRK_signal~RP1-2~vWFA |
| 3 | Streptococcus agalactiae | YSIRK_signal~SH3~GBS_Bsp-like |
| 3 | Staphylococcus capitis | YSIRK_signal~SasC_Mrp_aggreg~hyperosmo_Ebh |
| 3 | Streptococcus suis | YSIRK_signal~rne~GAGBD~G5~PRK10819~G5 |
| 3 | Streptococcus | YSIRK_signal~G5~zmp_18_rpt |
| 3 | Streptococcus infantis | YSIRK_signal~MSCRAMM_SdrC~LamG |
| 3 | Streptococcus | YSIRK_signal~marine_srt_targ~vWFA |
| 3 | Streptococcus australis | YSIRK_signal~Peptidases_S8_S53~PA~fn3_5~flgD |
| 3 | Streptococcus pneumoniae | YSIRK_signal~PTZ00121~G5~Collagen~Glutenin_hmw~Collagen~RPT_S_cricet |
| 3 | Streptococcus | YSIRK_signal~MSCRAMM_SdrD~GH18_chitinase-like |
| 3 | Streptococcus pneumoniae | YSIRK_signal~PRK11633~PTZ00121~Chb~G5 |
| 3 | Lactobacillus jensenii | YSIRK_signal~pip_yhgE_Nterm |
| 3 | Streptococcus suis | YSIRK_signal~NLPC_P60~SH3 |
| 3 | Staphylococcus | YSIRK_signal~DUF4678~Abhydrolase |
| 3 | Streptococcus | YSIRK_signal~MSCRAMM_SdrC~G5 |
| 3 | Streptococcus parasanguinis | YSIRK_signal~GH18_chitinase-like~F5_F8_type_C~Big_3~PAT1~Amelogenin |
| 3 | Streptococcus | YSIRK_signal~DUF1633~G5~Peptidase_M26_N~Peptidase_M26_C |
| 3 | Streptococcus oralis | YSIRK_signal~Tryp_SPc~AfuC~Calx-beta~PHA03247~G5 |
| 3 | Streptococcus mitis | YSIRK_signal~PTZ00121~hyperosmo_Ebh~Rib~PHA03247 |
| 3 | Streptococcus pneumoniae | YSIRK_signal~RICH~YkuD_like |
| 3 | Staphylococcus | YSIRK_signal~DUF4775~Abhydrolase |
| 3 | Terrabacteria group | YSIRK_signal~MSCRAMM_SdrC~FctA |
| 3 | Streptococcus | YSIRK_signal~Tryp_SPc~AfuC~Calx-beta~PTZ00121~PTZ00449 |
| 3 | Staphylococcus haemolyticus | YSIRK_signal~Mplasa_alph_rch~MG1~Herpes_BLLF1~MG1 |
| 3 | Staphylococcus haemolyticus | YSIRK_signal~MSCRAMM_SdrD~MG1~Herpes_BLLF1~MG1~PARM~MG1 |
| 3 | Lactobacillus fermentum | YSIRK_signal~MucBP~PRK06130 |
| 3 | Lactobacillus reuteri | YSIRK_signal~PRK10856~MucBP~PHA03247 |
| 3 | Streptococcus sp. I-P16 | MSCRAMM_SdrC~YSIRK_signal~GH18_chitinase-like~F5_F8_type_C~Big_3~PTZ00449~Amelogenin |
| 3 | Streptococcus pneumoniae | YSIRK_signal~PTZ00121~GH18_chitinase-like~F5_F8_type_C~Big_3~FIVAR |
| 3 | Streptococcus pneumoniae | YSIRK_signal~Atrophin-1~Smc~DUF3584 |
| 3 | Streptococcus pneumoniae | YSIRK_signal~motB |
| 3 | Streptococcus pneumoniae | YSIRK_signal~RICH~Smc~SMC_prok_A~RICH~COG5263 |
| 3 | Streptococcus pneumoniae | YSIRK_signal~RICH~CAF-1_p150~COG5263 |
| 3 | Streptococcus pneumoniae | YSIRK_signal~DUF4775~G5~Collagen~RPT_S_cricet~Cornifin |
| 3 | Staphylococcus | YSIRK_signal~MSCRAMM_SdrC~PRK07764~Herpes_BLLF1 |
| 3 | Staphylococcus | YSIRK_signal~NEAT~NEAT~MSCRAMM_SdrD |
| 3 | Staphylococcus capitis | YSIRK_signal~NEAT~NEAT~PRK05901 |
| 3 | Streptococcus pneumoniae | YSIRK_signal~PRK12704~RICH |
| 3 | Streptococcus pneumoniae | YSIRK_signal~PRK13914~G5~Collagen |
| 3 | Streptococcus pneumoniae | YSIRK_signal~DUF4775~G5~Glutenin_hmw~Collagen~Glutenin_hmw~Collagen~RPT_S_cricet |
| 3 | Lactobacillales | YSIRK_signal~PRK14971 |
| 3 | Streptococcus | PTZ00121~YSIRK_signal~CshA_fibril_rpt~repeat_SSSPR51 |
| 3 | Aerococcaceae | YSIRK_signal~G5~YabE~G5~PTZ00449 |
| 3 | Streptococcus azizii | YSIRK_signal~MSCRAMM_SdrC~Rib~He_PIG |
| 3 | Streptococcus mitis | YSIRK_signal~PRK05035~CshA_fibril_rpt |
| 3 | Staphylococcus aureus | YSIRK_signal~MSCRAMM_SdrC~NRIP1_repr_2~Abhydrolase |
| 3 | Streptococcus pseudopneumoniae | YSIRK_signal~MSCRAMM_SdrC~Mitofilin~FIVAR |
| 3 | Streptococcus pneumoniae | YSIRK_signal~RICH~PTZ00121~RICH~APG6~COG5263 |
| 3 | Streptococcus pneumoniae | YSIRK_signal~RICH~Smc~SMC_prok_B~RICH~PTZ00449~COG5263 |
| 3 | Streptococcus | YSIRK_signal~MSCRAMM_SdrC~AfuC~Calx-beta~DUF499~G5 |
| 3 | Streptococcus salivarius | YSIRK_signal~IsdH_HarA~lectin_L-type |
| 3 | Streptococcus | YSIRK_signal~PTZ00449~PHA03247 |
| 3 | Streptococcus | IsdB~YSIRK_signal~PTZ00121 |
| 3 | Streptococcus | YSIRK_signal~PARM~pullulan_Gpos |
| 3 | Staphylococcus cohnii | YSIRK_signal~MDN1~SMC_prok_B~PRK14949~PTZ00121 |
| 3 | Streptococcus pneumoniae | YSIRK_signal~hyperosmo_Ebh~Periplasmic_Binding_Protein_Type_1~hyperosmo_Ebh~PTZ00121~V_Alix_like |
| 3 | Streptococcus pneumoniae | YSIRK_signal~hyperosmo_Ebh~DUF1542~Alpha-E |
| 3 | Staphylococcus | YSIRK_signal~MSCRAMM_SdrC~SasC_Mrp_aggreg~DUF1542~235kDa-fam~DUF1542 |
| 3 | Streptococcus suis | YSIRK_signal~Mac-1~termin_org_DnaJ |
| 2 | Streptococcus suis | YSIRK_signal~MucBP~Cornifin |
| 2 | Streptococcus | YSIRK_signal~G5~NESP55~G5~FIVAR~Peptidase_M26_N~Peptidase_M26_C |
| 2 | Bacilli | YSIRK_signal~MSCRAMM_SdrC~PRK13914~Abhydrolase |
| 2 | Staphylococcus saprophyticus | YSIRK_signal~Red1 |
| 2 | Streptococcus salivarius | YSIRK_signal~PRK11907~GH43_62_32_68_117_130~PTZ00449 |
| 2 | Streptococcus suis | YSIRK_signal~PRK13914~GAGBD~G5~PRK10819~G5 |
| 2 | Streptococcus | YSIRK_signal~PRK08691~LacZ~Big_4~G5 |
| 2 | Streptococcus | YSIRK_signal~Chb~G5~TonB |
| 2 | Streptococcus | YSIRK_signal~PTZ00121~LacZ~Big_4~G5~rne |
| 2 | Aerococcus | YSIRK_signal~PRK13108~G5~Glutenin_hmw~Collagen |
| 2 | Streptococcus | MSCRAMM_SdrC~YSIRK_signal~GH18_chitinase-like~F5_F8_type_C~Big_3~FIVAR~TroA-like |
| 2 | Streptococcus | YSIRK_signal~PRK05035~PRK08026~CshA_fibril_rpt |
| 2 | Staphylococcus | MSCRAMM_SdrC~YSIRK_signal~rne~MARTX_Nterm~PTZ00121~PTZ00108 |
| 2 | Streptococcus | YSIRK_signal~PRK13108~FctA~Antigen_C~FctA~Antigen_C~FctA~Antigen_C |
| 2 | Streptococcus | IsdH_HarA~YSIRK_signal~AfuC~Calx-beta~G5 |
| 2 | Streptococcus | YSIRK_signal~Tryp_SPc~AfuC~Calx-beta~flagell_flgL~DUF1542 |
| 2 | Streptococcus | YSIRK_signal~GH18_chitinase-like~F5_F8_type_C~Big_3~FIVAR~PTZ00449~Amelogenin |
| 2 | Streptococcus pneumoniae | YSIRK_signal~MSCRAMM_SdrD~LamG~Sialidase~Trypan_PARP |
| 2 | Streptococcus cristatus | YSIRK_signal~G5~zmp_18_rpt~PHA03379~G5~FIVAR~Peptidase_M26_N~Peptidase_M26_C |
| 2 | Lactobacillus salivarius | YSIRK_signal~SMC_prok_B~Surf_Exclu_PgrA |
| 2 | Staphylococcus | YSIRK_signal~MDN1~MSCRAMM_SdrC~Drf_FH1 |
| 2 | Gemella haemolysans | YSIRK_signal~Smc~MucBP~PTZ00449 |
| 2 | Streptococcus pneumoniae | YSIRK_signal~IsdB~LacZ~Big_4~YabE~G5 |
| 2 | Facklamia hominis | YSIRK_signal~AIF-MLS~Flg_new~Rib |
| 2 | Streptococcus pneumoniae | YSIRK_signal~RICH~PRK11633 |
| 2 | Abiotrophia defectiva | YSIRK_signal~Surf_Exclu_PgrA |
| 2 | Aerococcus | YSIRK_signal~PRK10856~G5~YabE~G5~YabE~G5~IMCp~PTZ00449 |
| 2 | Streptococcus pneumoniae | YSIRK_signal~hyperosmo_Ebh~Mitofilin~DUF1542 |
| 2 | Streptococcus pyogenes | YSIRK_signal~Fmp27_WPPW |
| 2 | Streptococcus pneumoniae | YSIRK_signal~TIGR03809~hyperosmo_Ebh |
| 2 | Streptococcus henryi | YSIRK_signal~MSCRAMM_SdrC~GbpC |
| 2 | Aerococcus | YSIRK_signal~G5~Pro-rich |
| 2 | Staphylococcus | YSIRK_signal~NEAT~NEAT~MSCRAMM_SdrC |
| 2 | Streptococcus | YSIRK_signal~DedD~CBM6-CBM35-CBM36_like~beta_helix_1 |
| 2 | Lactobacillales | YSIRK_signal~235kDa-fam |
| 2 | Streptococcus suis | YSIRK_signal~MSCRAMM_SdrC~MucBP~Cornifin |
| 2 | Streptococcus suis | YSIRK_signal~MSCRAMM_SdrC~GH18_chitinase-like~F5_F8_type_C~Big_3~TBPIP~FIVAR~G5 |
| 2 | Streptococcus oralis | YSIRK_signal~Tryp_SPc~AfuC~Calx-beta~FtsN~PTZ00449 |
| 2 | Streptococcus | YSIRK_signal~PRK05035~DUF1542 |
| 2 | Streptococcus pneumoniae | YSIRK_signal~G5~Glutenin_hmw~G5~Peptidase_M26_N~Peptidase_M26_C |
| 2 | Streptococcus suis | YSIRK_signal~rne~MucBP~Cornifin~repeat_SSSPR51~MucBP |
| 2 | Streptococcus parasanguinis | YSIRK_signal~MSCRAMM_SdrC~Peptidases_S8_S53~PA~fn3_5~FlgD_ig~LPXTG_double |
| 2 | Streptococcus gordonii | YSIRK_signal~MSCRAMM_SdrC~DUF3350~Peptidases_S8_S53~PA~fn3_5~FlgD_ig~PRK11907 |
| 2 | Streptococcus gordonii | MSCRAMM_SdrC~YSIRK_signal~LacZ~Big_4~G5~rRNA_processing |
| 2 | Streptococcus | YSIRK_signal~G5~NESP55~zmp_18_rpt~G5~FIVAR~Peptidase_M26_N~Peptidase_M26_C |
| 2 | Streptococcus pneumoniae | YSIRK_signal~DUF4775~G5~RPT_S_cricet~Collagen~Glutenin_hmw~Collagen~RPT_S_cricet~Cornifin |
| 2 | Streptococcus | YSIRK_signal~COG4372 |
| 2 | Finegoldia magna | MSCRAMM_SdrC~YSIRK_signal~Flg_new~Rib |
| 2 | Staphylococcus haemolyticus | YSIRK_signal~MSCRAMM_SdrC~PRK10927~ftsN~YncE~MSCRAMM_SdrC~Herpes_LMP1 |
| 2 | Staphylococcus epidermidis | YSIRK_signal~PRK07764~Herpes_BLLF1~SWI-SNF_Ssr4 |
| 2 | Streptococcus thermophilus | YSIRK_signal~rne~MucBP~PTZ00449 |
| 2 | Streptococcus | YSIRK_signal~PRK11907~GH43_62_32_68_117_130 |
| 2 | Streptococcus | YSIRK_signal~PHA03255~GH43_62_32_68_117_130 |
| 2 | Staphylococcus haemolyticus | YSIRK_signal~PRK10927~ftsN~YncE~MSCRAMM_SdrC~Herpes_LMP1 |
| 2 | Streptococcus pneumoniae | YSIRK_signal~hyperosmo_Ebh~DUF1542~Pneumo_att_G~PRK03918 |
| 2 | Streptococcus pneumoniae | YSIRK_signal~PTZ00121~RICH~CW_binding_1 |
| 2 | Staphylococcus aureus | YSIRK_signal~PTZ00121~Rib~hyperosmo_Ebh~Rib~MSCRAMM_SdrC |
| 2 | Streptococcus pneumoniae | YSIRK_signal~DUF4775~G5~Glutenin_hmw~RPT_S_cricet~Collagen~RPT_S_cricet~Collagen~RPT_S_cricet |
| 2 | Lactobacillus jensenii | YSIRK_signal~MSCRAMM_SdrC~FIVAR~GA |
| 2 | Bacillales | YSIRK_signal~MSCRAMM_SdrC~Abhydrolase~Rib |
| 2 | Streptococcus mitis | IsdB~YSIRK_signal~CshA_fibril_rpt~repeat_SSSPR51~Amelogenin |
| 2 | Streptococcus pseudopneumoniae | YSIRK_signal~PTZ00121~hyperosmo_Ebh~Rib |
| 2 | Streptococcus parasanguinis | YSIRK_signal~AfuC~Calx-beta~DUF499~G5 |
| 2 | Streptococcus | YSIRK_signal~pylS~LacZ~Big_4~G5~rne |
| 2 | Streptococcus equinus | YSIRK_signal~MSCRAMM_SdrC~Peptidases_S8_S53~PA~fn3_5~FlgD_ig~FIVAR~PRK10476~FIVAR |
| 2 | Streptococcus equinus | YSIRK_signal~rne~Peptidases_S8_S53~PA~fn3_5~Smc |
| 2 | Staphylococcus | YSIRK_signal~MSCRAMM_SdrC~hyperosmo_Ebh~SMC_prok_B~vATP-synt_E |
| 2 | Streptococcus pneumoniae | YSIRK_signal~RICH~Smc~RICH~PRK14948~RICH |
| 2 | Streptococcus pneumoniae | YSIRK_signal~DUF4775~G5~Collagen~Glutenin_hmw~Collagen~RPT_S_cricet~Collagen~RPT_S_cricet~PHA03247 |
| 2 | Streptococcus suis | YSIRK_signal~Mac-1~PARM |
| 2 | Streptococcus pneumoniae | YSIRK_signal~DUF4775~G5~Collagen~Glutenin_hmw~RPT_S_cricet~Collagen~RPT_S_cricet |
| 2 | Streptococcus suis | YSIRK_signal~MSCRAMM_SdrC~YhaN |
| 2 | Aerococcus christensenii | YSIRK_signal~SMC_prok_B~FIVAR |
| 2 | Streptococcus salivarius | YSIRK_signal~DedD~CBM6-CBM35-CBM36_like~beta_helix_1~EBP50_C |
| 2 | Streptococcus pneumoniae | YSIRK_signal~RICH~SMC_N~COG5263 |
| 2 | Staphylococcus aureus | YSIRK_signal~PRK10905~SasC_Mrp_aggreg~DUF1542 |
| 2 | Staphylococcus aureus | YSIRK_signal~PRK10905~SasC_Mrp_aggreg~DUF1542~Smc~DUF1542 |
| 2 | Streptococcus suis | YSIRK_signal~rne~MucBP~MISS~repeat_SSSPR51~MucBP |
| 2 | Lactobacillus iners | MSCRAMM_SdrC~YSIRK_signal~DUF515~Rib |
| 2 | Streptococcus pneumoniae | YSIRK_signal~RICH~PTZ00121~COG5263~glucan_65_rpt~CW_binding_1~glucan_65_rpt |
| 2 | Streptococcus | YSIRK_signal~G5~Peptidase_M26_C |
| 2 | Streptococcus pneumoniae | YSIRK_signal~rne~G5~Peptidase_M26_N~PRK12758~Peptidase_M26_C |
| 2 | Bacillales | YSIRK_signal~TonB_N~G5~Peptidase_M26_C |
| 2 | Streptococcus pneumoniae | YSIRK_signal~hyperosmo_Ebh~DUF1542~PRK03918 |
| 2 | Lactobacillus iners | MSCRAMM_SdrC~YSIRK_signal~Flg_new~MucBP |
| 2 | Streptococcus pneumoniae | YSIRK_signal~RICH~Smc |
| 2 | Streptococcus pneumoniae | YSIRK_signal~MARCKS~hyperosmo_Ebh~DUF1542~PDCD7 |
| 2 | Streptococcus pneumoniae | YSIRK_signal~RICH~Pneumo_att_G |
| 2 | Streptococcus pneumoniae | YSIRK_signal~DUF4775~G5~Glutenin_hmw~Collagen~RPT_S_cricet~Glutenin_hmw~Collagen~RPT_S_cricet |
| 2 | Streptococcus pneumoniae | YSIRK_signal~MSCRAMM_SdrD~Smc~DUF3584 |
| 2 | Streptococcus pneumoniae | YSIRK_signal~Pectate_lyase_3 |
| 2 | Staphylococcus aureus | YSIRK_signal~PRK07764~SasC_Mrp_aggreg~Mitofilin~DUF1542 |
| 2 | Lactobacillus | YSIRK_signal~PRK15313 |
| 2 | Streptococcus | YSIRK_signal~Tryp_SPc~AfuC~Calx-beta~PRK10263~PTZ00449 |
| 2 | Streptococcus thermophilus | YSIRK_signal~MSCRAMM_SdrD~Trypan_PARP |
| 2 | Streptococcus suis | YSIRK_signal~MSCRAMM_SdrC~GAGBD~G5~PRK13108~G5~COG4932 |
| 2 | Streptococcus pseudopneumoniae | YSIRK_signal~MSCRAMM_SdrC~tolA_full~PTZ00449 |
| 2 | Streptococcus sanguinis | YSIRK_signal~COG5665~CshA_fibril_rpt |
| 2 | Lactobacillus | MSCRAMM_SdrC~YSIRK_signal~SMC_prok_B~Smc~PTZ00121~SLAP |
| 2 | Bacilli | YSIRK_signal~AlphaC_N |
| 2 | Granulicatella | YSIRK_signal~GbpC~repeat_SSSPR51 |
| 2 | Streptococcus oralis | YSIRK_signal~WEMBL~CshA_fibril_rpt~repeat_SSSPR51 |
| 2 | Streptococcus suis | YSIRK_signal~IsdB~Peptidases_S8_S53~PA~fn3_5 |
| 2 | Streptococcus suis | YSIRK_signal~rne~GAGBD~G5~SucB_Actino~G5 |
| 2 | Streptococcus suis | MSCRAMM_SdrC~YSIRK_signal~GAGBD~G5~SucB_Actino~G5 |
| 2 | Streptococcus | YSIRK_signal~MSCRAMM_SdrD~GH18_chitinase-like~F5_F8_type_C~Big_3~FIVAR~G5 |
| 2 | Streptococcus | YSIRK_signal~MAP2_projctn~GH18_chitinase-like~F5_F8_type_C~Big_3~FIVAR~G5 |
| 2 | Streptococcus pneumoniae | YSIRK_signal~DUF1542~III |
| 2 | Streptococcus pneumoniae | YSIRK_signal~DUF1542~alaS |
| 2 | Streptococcus | MSCRAMM_SdrC~YSIRK_signal~FctA~Antigen_C~FctA |
| 2 | Streptococcus infantis | YSIRK_signal~rne~Tryp_SPc~AfuC~Calx-beta~SMC_prok_B~DUF1542~G5 |
| 2 | Streptococcus mitis | YSIRK_signal~MSCRAMM_SdrC~Borrelia_P83~PTZ00449~MucBP |
| 2 | Staphylococcus | YSIRK_signal~Herpes_BLLF1~SasC_Mrp_aggreg~DUF1542 |
| 2 | Abiotrophia defectiva | YSIRK_signal~PRK12372~PRK10819~CshA_fibril_rpt |
| 2 | Streptococcus | YSIRK_signal~MSCRAMM_SdrC~COG3942~SH3 |
| 2 | Streptococcus oralis | YSIRK_signal~PRK12373~vWFA |
| 2 | Streptococcus mitis | YSIRK_signal~rne~G5~termin_org_DnaJ~G5~Peptidase_M26_N~Peptidase_M26_C |
| 2 | Streptococcus mitis | YSIRK_signal~G5~PTZ00121~G5~Peptidase_M26_N~Peptidase_M26_C |
| 2 | Lactobacillus jensenii | YSIRK_signal~Liste_lipo_26~DUF285~EmrA~GA~FIVAR~GA~PTZ00121~FIVAR |
| 2 | Streptococcus mitis | YSIRK_signal~MSCRAMM_SdrC~LacZ~Big_4~G5~DUF4775 |
| 2 | Streptococcus salivarius | YSIRK_signal~MSCRAMM_SdrC~FctA~Antigen_C |
| 2 | Staphylococcus epidermidis | YSIRK_signal~SasC_Mrp_aggreg~PTZ00121~DUF1542 |
| 2 | Bacilli | YSIRK_signal~SMC_N |
| 2 | Staphylococcus capitis | YSIRK_signal~PRK07764~SWI-SNF_Ssr4 |
| 2 | Streptococcus australis | MSCRAMM_SdrC~YSIRK_signal~PRK14950 |
| 2 | Lactobacillus iners | YSIRK_signal~PRK02224~PTZ00121 |
| 2 | Staphylococcus | YSIRK_signal~SMC_N~Abhydrolase |
| 2 | Streptococcus sobrinus | YSIRK_signal~Smc~PTZ00121~GbpC |
| 2 | Streptococcus | IsdB~YSIRK_signal~SasC_Mrp_aggreg~DUF1542 |
| 2 | Lactobacillus acidophilus | YSIRK_signal~MSCRAMM_SdrC~PRK11633 |
| 2 | Streptococcus suis | YSIRK_signal~RNase_Y~PLN02226~PTZ00449 |
| 2 | Streptococcus agalactiae | YSIRK_signal~SMC_N~GAGBD~RICH~PTZ00449 |
| 2 | Streptococcus | YSIRK_signal~MSCRAMM_SdrC~CBM6-CBM35-CBM36_like~beta_helix_1 |
| 2 | Streptococcus criceti | YSIRK_signal~rne~Flg_new |
| 2 | Gemella bergeri | YSIRK_signal~PRK03918~PTZ00449~repeat_SSSPR51 |
| 2 | Streptococcus pneumoniae | YSIRK_signal~PRK13108~PRK14949~hyperosmo_Ebh |
| 2 | Streptococcus pneumoniae | YSIRK_signal~Pgu1~COG3464 |
| 2 | Staphylococcus aureus | YSIRK_signal~PTZ00121~lectin_L-type~Rib~MSCRAMM_SdrC |
| 2 | Streptococcus pneumoniae | YSIRK_signal~Pgu1~Tra8 |
| 2 | Streptococcus pneumoniae | YSIRK_signal~Pgu1~DUF4096 |
| 2 | Streptococcus mitis | YSIRK_signal~Abhydrolase~repeat_SSSPR51 |
| 2 | Streptococcus | YSIRK_signal~PHA02053 |
| 2 | Streptococcus agalactiae | YSIRK_signal~SH3 |
| 2 | Streptococcus salivarius | MSCRAMM_SdrC~YSIRK_signal~hyperosmo_Ebh~COG4932~Herpes_BLLF1~PRK08026~COG4932 |
| 2 | Streptococcus canis | YSIRK_signal~AlphaC_N~Rib |
| 2 | Streptococcus suis | YSIRK_signal~Atrophin-1~PRK10263~PTZ00449~Herpes_BLLF1~Peptidase_M26_N~Peptidase_M26_C |
| 2 | Streptococcus uberis | YSIRK_signal~MSCRAMM_SdrC~lectin_L-type |
| 2 | Streptococcus suis | YSIRK_signal~DUF4775~G5~YabE~G5~PRK03427~Peptidase_M26_N~Peptidase_M26_C |
| 2 | Streptococcus pneumoniae | YSIRK_signal~RNase_Y~RICH~Trypan_PARP |
| 2 | Streptococcus pneumoniae | YSIRK_signal~hyperosmo_Ebh~DUF1542~TIGR02680~DUF1542 |
| 2 | Streptococcus pneumoniae | YSIRK_signal~PTZ00121~hyperosmo_Ebh~DUF1542 |
| 2 | Streptococcus suis | YSIRK_signal~MucBP~PRK08026~PHA03247 |
| 2 | Streptococcus | YSIRK_signal~PRK11907~pullulan_Gpos~AmyAc_family~pullulan_Gpos |
| 2 | Streptococcus suis | YSIRK_signal~Atrophin-1~PRK10263~G5~PRK10263~Trypan_PARP~Peptidase_M26_N~Peptidase_M26_C |
| 2 | Streptococcus suis | YSIRK_signal~G5~YabE~G5~PRK03427~Peptidase_M26_N~Peptidase_M26_C |
| 2 | Streptococcus suis | YSIRK_signal~Atrophin-1~G5~rne~G5~PRK10263~G5~Peptidase_M26_N~Peptidase_M26_C |
| 2 | Streptococcus suis | YSIRK_signal~PRK08026~DUF1542 |
| 2 | Staphylococcus | YSIRK_signal~IsdH_HarA~pullulan_Gpos |
| 2 | Streptococcus pneumoniae | YSIRK_signal~MSCRAMM_SdrC~InfB |
| 2 | Staphylococcus cohnii | YSIRK_signal~2A1904~Abhydrolase |
| 2 | Streptococcus pneumoniae | YSIRK_signal~MARCKS~hyperosmo_Ebh~PTZ00121~DUF1542 |
| 2 | Streptococcus | MSCRAMM_SdrC~YSIRK_signal~hyperosmo_Ebh~DUF1542 |
| 2 | Streptococcus | YSIRK_signal~SucB_Actino |
| 2 | Streptococcus mitis | IsdB~YSIRK_signal~rne~CshA_fibril_rpt~repeat_SSSPR51~PHA03247 |
| 2 | Streptococcus suis | YSIRK_signal~GAGBD~PRK12323~PRK13108~G5 |
| 2 | Streptococcus oralis | IsdH_HarA~YSIRK_signal~Tryp_SPc~AfuC~Calx-beta~NESP55~PTZ00449~G5 |
| 2 | Streptococcus azizii | YSIRK_signal~BASP1~GAG_Lyase |
| 2 | Streptococcus gallolyticus | YSIRK_signal~MSCRAMM_SdrC~SH3~GBS_Bsp-like~SH3~GBS_Bsp-like~SH3 |
| 2 | Staphylococcus hominis | YSIRK_signal~MSCRAMM_SdrC~hyperosmo_Ebh~mukB |
| 2 | Dolosigranulum pigrum | YSIRK_signal~MSCRAMM_SdrC~LamG~Sialidase~Trypan_PARP~NDPk |
| 2 | Streptococcus mitis | IsdB~YSIRK_signal~rne~CshA_fibril_rpt~repeat_SSSPR51~PHA03247~repeat_SSSPR51 |
| 2 | Streptococcus mitis | YSIRK_signal~PTZ00121~SMC_prok_B~CshA_fibril_rpt |
| 2 | Enterococcus hirae | YSIRK_signal~G5~MAD~Peptidase_M26_N~Peptidase_M26_C |
| 2 | Streptococcus | YSIRK_signal~MSCRAMM_SdrD~COG3942~SH3~GBS_Bsp-like~SH3 |
| 2 | Staphylococcus haemolyticus | YSIRK_signal~Mplasa_alph_rch~MG1~PARM~MG1 |
| 2 | Streptococcus agalactiae | YSIRK_signal~G5~Collagen~PRK14959~Collagen~Glutenin_hmw~PTZ00449 |
| 2 | Streptococcus suis | YSIRK_signal~Atrophin-1~PHA03247~G5~Peptidase_M26_N~Peptidase_M26_C |
| 2 | Streptococcus suis | YSIRK_signal~Retinal~G5~PRK04335~PARM~G5~PRK10263~G5~Peptidase_M26_N~Peptidase_M26_C |
| 2 | Streptococcus suis | YSIRK_signal~Atrophin-1~rne~PRK14960~G5~Peptidase_M26_N~Peptidase_M26_C |
| 2 | Streptococcus mitis | YSIRK_signal~ftsN~Chb~G5~COG5263 |
| 2 | Streptococcus | YSIRK_signal~MSCRAMM_SdrC~Glyco_hydro_20b~NAGidase~F5_F8_type_C |
| 2 | Aerococcus sp. HMSC23C02 | YSIRK_signal~G5~PRK12678~Glutenin_hmw~Collagen |
| 2 | Lactobacillus gallinarum | YSIRK_signal~Alanine_zipper |
| 2 | Lactobacillus | YSIRK_signal~PRK10856~MucBP |
| 2 | Aerococcus urinae | YSIRK_signal~rne~G5~Collagen~Glutenin_hmw~Collagen |
| 2 | Streptococcus acidominimus | YSIRK_signal~PRK14951~Peptidases_S8_S53~PA~fn3_5 |
| 2 | Streptococcus cuniculi | YSIRK_signal~Herpes_BLLF1~He_PIG~pullulan_Gpos |
| 2 | Streptococcus | YSIRK_signal~COG5665~MucBP~PHA03247 |
| 2 | Streptococcus oralis | YSIRK_signal~PRK08581~SbcC~GAGBD~PHA03247 |
| 2 | Staphylococcus aureus | YSIRK_signal~MSCRAMM_SdrC~FAP~SasC_Mrp_aggreg~DUF1542~Smc~DUF1542 |
| 2 | Globicatella sp. HMSC072A10 | YSIRK_signal~PTZ00341~GH_101_like~Glyco_hyd_101C~F5_F8_type_C |
| 2 | Streptococcus oralis | YSIRK_signal~Tryp_SPc~AfuC~Calx-beta~SbcC~DUF1542~PHA03247~G5 |
| 2 | Lactobacillus melliventris | YSIRK_signal~5~MDN1~YhgE~Smc~SLAP |
| 2 | Gemella bergeri | YSIRK_signal~MSCRAMM_SdrC~DUF5401~SerS |
| 2 | Streptococcus | YSIRK_signal~Tryp_SPc~AfuC~Calx-beta~ftsN~PHA03247~PTZ00449 |
| 2 | Streptococcus | IsdB~YSIRK_signal~hyperosmo_Ebh~DUF1542 |
| 2 | Streptococcus | YSIRK_signal~PRK05035~CshA_fibril_rpt~PTZ00449 |
| 2 | Streptococcus sp. HMSC066F01 | YSIRK_signal~MSCRAMM_SdrC~Glyco_hyd_65N_2~ATH1~FN3 |
| 2 | Streptococcus | YSIRK_signal~LamG~Sialidase~GH_101_like~Glyco_hyd_101C~F5_F8_type_C |
| 2 | Streptococcus | YSIRK_signal~PHA03247~FIVAR~Peptidase_M26_N~Peptidase_M26_C |
| 2 | Staphylococcus pseudintermedius | YSIRK_signal~B~PTZ00121~LysM |
| 2 | Staphylococcus aureus | YSIRK_signal~SMC_prok_B~PTZ00440 |
| 2 | Streptococcus porcinus | MSCRAMM_SdrC~YSIRK_signal~FliA~Collagen~RPT_S_cricet~PTZ00449 |
| 2 | Streptococcus pneumoniae | YSIRK_signal~DUF4775~G5~Collagen~Glutenin_hmw~Collagen~RPT_S_cricet~Collagen~RPT_S_cricet |
| 2 | Streptococcus pneumoniae | YSIRK_signal~PRK05035~G5~Collagen~RPT_S_cricet~2A1904~Glutenin_hmw~Collagen~RPT_S_cricet |
| 2 | Staphylococcus pettenkoferi | YSIRK_signal~PRK11907~MSCRAMM_SdrD~Abhydrolase |
| 2 | Lactobacillus reuteri | YSIRK_signal~DUF1542~MDN1~DUF1542 |
| 2 | Streptococcus | YSIRK_signal~TonB_N |
| 2 | Streptococcus mitis | MSCRAMM_SdrC~YSIRK_signal~G5~repeat_SSSPR51 |
| 2 | Streptococcus mitis | YSIRK_signal~PRK12495~Rib |
| 2 | Streptococcus mitis | IsdB~YSIRK_signal~F5_F8_type_C~Glyco_hydro_20b~GH20_hexosaminidase~F5_F8_type_C~Glyco_hydro_20b~NAGidase~F5_F8_type_C |
| 2 | Streptococcus | YSIRK_signal~rne~G5~2A1904~G5~Peptidase_M26_N~Peptidase_M26_C |
| 2 | Streptococcus | YSIRK_signal~Mitofilin~Rib~PHA03247 |
| 2 | Streptococcus mitis | YSIRK_signal~rne~G5~YabE~G5~Peptidase_M26_N~Peptidase_M26_C |
| 2 | Streptococcus oralis | YSIRK_signal~IsdB~Thiol_cytolysin~Thiol_cytolys_C |
| 2 | Streptococcus salivarius | YSIRK_signal~PRK10856~hyperosmo_Ebh |
| 2 | Staphylococcus | YSIRK_signal~PRK08581~SasC_Mrp_aggreg |
| 2 | Lactobacillus agilis | YSIRK_signal~PRK12495~MucBP |
| 2 | Finegoldia magna | YSIRK_signal~Flg_new~Rib~Trypan_PARP |
| 2 | Lactobacillus | YSIRK_signal~PHA03255~DUF1542 |
| 2 | Lactobacillus | YSIRK_signal~FIVAR~DUF1542~PTZ00121~DUF1542~PTZ00121~DUF1542 |
| 2 | Lactobacillus reuteri | YSIRK_signal~PRK10856~He_PIG~Rib |
| 2 | Lactobacillus reuteri | YSIRK_signal~FAP~PTZ00449~PRK15313 |
| 2 | Staphylococcus delphini | MSCRAMM_SdrC~YSIRK_signal~rne~GH_101_like~Glyco_hyd_101C~Herpes_BLLF1 |
| 2 | Staphylococcus | YSIRK_signal~B~LysM~PRK13914 |
| 2 | Lactobacillus johnsonii | MSCRAMM_SdrC~YSIRK_signal~DUF5011 |
| 2 | Lactobacillus johnsonii | YSIRK_signal~Ebola_NP |
| 2 | Streptococcus | YSIRK_signal~DUF5011~hyperosmo_Ebh |
| 2 | Streptococcus parasanguinis | MSCRAMM_SdrC~YSIRK_signal~PHA03247~Herpes_BLLF1 |
| 2 | Streptococcus | YSIRK_signal~HMMR_N~hyperosmo_Ebh~PHA03247 |
| 2 | Staphylococcus hominis | YSIRK_signal~MSCRAMM_SdrC~hyperosmo_Ebh~CCDC158 |
| 2 | Streptococcus mitis | MSCRAMM_SdrC~YSIRK_signal~hyperosmo_Ebh~PHA03247 |
| 2 | Lactobacillus | YSIRK_signal~PTZ00121~FIVAR~SLAP |
| 2 | Lactobacillus | YSIRK_signal~COG4932 |
| 2 | Streptococcus suis | YSIRK_signal~PTZ00121~GAGBD~G5 |
| 2 | Streptococcus suis | YSIRK_signal~GAGBD~rne~PHA03247~G5 |
| 2 | Lactobacillales | YSIRK_signal~PRK05035~PTZ00121 |
| 2 | Streptococcus suis | YSIRK_signal~Mac-1~rne~PRK12495 |
| 2 | Peptoniphilaceae | YSIRK_signal~PTZ00121~Flg_new~hyperosmo_Ebh |
| 2 | Streptococcus pneumoniae | YSIRK_signal~DRE_TIM_metallolyase |
| 2 | Staphylococcus cohnii | YSIRK_signal~MSCRAMM_SdrC~SMC_prok_B~PTZ00121 |
| 2 | Staphylococcus | YSIRK_signal~MSCRAMM_SdrC~DUF1542~YhgE~DUF1542~Smc |
| 2 | Staphylococcus agnetis | YSIRK_signal~Endomucin~ftsN~Abhydrolase |
| 2 | Staphylococcus auricularis | MSCRAMM_SdrC~YSIRK_signal~hyperosmo_Ebh~Rib~hyperosmo_Ebh~Rib~MSCRAMM_SdrC |
| 2 | Staphylococcus auricularis | MSCRAMM_SdrC~YSIRK_signal~PRK14949~PHA03247~Glutenin_hmw |
| 2 | Staphylococcus capitis | YSIRK_signal~NEAT~MSCRAMM_SdrC |
| 2 | Staphylococcus chromogenes | MSCRAMM_SdrC~YSIRK_signal~PRK14949~SPATA3~Rib |
| 2 | Lactobacillus iners | MSCRAMM_SdrD~YSIRK_signal~tolA |
| 2 | Staphylococcus equorum | YSIRK_signal~MSCRAMM_SdrC~PRK08581~YncE |
| 2 | Streptococcus suis | YSIRK_signal~rne~WD40 |
| 2 | Staphylococcus sciuri | YSIRK_signal~DUF4775~lectin_L-type~MucBP |
| 2 | Lactobacillus salivarius | YSIRK_signal~Abhydrolase~PldB~GA~FIVAR~PTZ00449 |
| 2 | Lactobacillus salivarius | YSIRK_signal~SMC_prok_B~PTZ00121 |
| 2 | Streptococcus | YSIRK_signal~hyperosmo_Ebh~DUF1542~PRK05901~DUF1542~PRK05035~DUF1542 |
| 2 | Streptococcus suis | YSIRK_signal~BASP1~termin_org_DnaJ~WD40 |
| 2 | Streptococcus suis | YSIRK_signal~rne~GAGBD~G5~DedD~G5 |
| 2 | Staphylococcus simulans | YSIRK_signal~IsdH_HarA~MSCRAMM_SdrD |
| 2 | Streptococcus | YSIRK_signal~MSCRAMM_SdrC~PRK08026~Rib |
| 2 | Streptococcus | YSIRK_signal~Tryp_SPc~Trypan_PARP~GH_101_like~Glyco_hyd_101C~F5_F8_type_C~PRK09418 |
| 2 | Streptococcus | YSIRK_signal~Abhydrolase~F5_F8_type_C |
| 2 | Streptococcus | YSIRK_signal~SbcC~hyperosmo_Ebh |
| 2 | Bacilli | YSIRK_signal~predic_Ig_block~PRK11633 |
| 2 | Streptococcus | YSIRK_signal~PTZ00121~PTZ00441 |
| 2 | Gemella sanguinis | YSIRK_signal~G5~SbcC~Peptidase_M26_N~Peptidase_M26_C |
| 2 | Streptococcus agalactiae | MSCRAMM_SdrC~YSIRK_signal~Peptidases_S8_S53~PA~fn3_5~SbcC~FIVAR |
| 2 | Lactobacillus panis | YSIRK_signal~PRK08691~choice_anch_A |
| 2 | Streptococcus anginosus | YSIRK_signal~MSCRAMM_SdrC~rne~SH3~GBS_Bsp-like~SH3~GBS_Bsp-like~Peptidase_M15~SH3 |
| 2 | Lactobacillus hamsteri | YSIRK_signal~MSCRAMM_SdrC~PHA03247~Herpes_BLLF1 |
| 2 | Lactobacillus kitasatonis | YSIRK_signal~MSCRAMM_SdrC~TonB |
| 2 | Lactobacillus iners | YSIRK_signal~IsdB~hyperosmo_Ebh~MG1 |
| 2 | Lactobacillus antri | YSIRK_signal~FIVAR~DUF1542 |
| 2 | Lactobacillus iners | YSIRK_signal~SMC_N~DUF4988 |
| 2 | Lactobacillus intestinalis | YSIRK_signal~tolA |
| 2 | Staphylococcus epidermidis | YSIRK_signal~PRK08581~MSCRAMM_SdrC~MSCRAMM_SdrD |
| 2 | Staphylococcus haemolyticus | YSIRK_signal~PRK08581~YncE~Herpes_LMP1~MSCRAMM_SdrC |
| 2 | Streptococcus pneumoniae | YSIRK_signal~RICH~Smc~RICH |
| 2 | Streptococcus suis | YSIRK_signal~GAGBD~rne~PRK12323~G5~COG4932 |
| 2 | Streptococcus suis | YSIRK_signal~Rib_recp_KP_reg~rne~PRK10263~Peptidase_M26_N~FhaB~Peptidase_M26_C |
| 2 | Streptococcus suis | YSIRK_signal~MSCRAMM_SdrC~Peptidase_M26_C~PL-6~G5 |
| 2 | Streptococcus suis | YSIRK_signal~PTZ00341~G5~YabE~G5~PRK03427~Peptidase_M26_N~Peptidase_M26_C |
| 2 | Streptococcus suis | YSIRK_signal~GH18_chitinase-like~F5_F8_type_C~Big_3~G5 |
| 2 | Streptococcus suis | YSIRK_signal~GAGBD~PTZ00449~G5 |
| 2 | Streptococcus suis | YSIRK_signal~SbcC~GA~FIVAR~hyperosmo_Ebh~rne~hyperosmo_Ebh~rne~MSCRAMM_SdrC~hyperosmo_Ebh |
| 2 | Streptococcus suis | YSIRK_signal~Aim21~G5~CAP |
| 2 | Streptococcus suis | YSIRK_signal~PTZ00121~PRK05901~PTZ00121 |
| 2 | Streptococcus suis | YSIRK_signal~PRK13914~GAGBD~G5~SucB_Actino~G5 |
| 2 | Streptococcus suis | YSIRK_signal~Mac-1~termin_org_DnaJ~rne~PRK10263 |
| 2 | Streptococcus suis | YSIRK_signal~PTZ00341~G5~YabE~G5~Neisseria_TspB~GGN~Peptidase_M26_N~Peptidase_M26_C |
| 2 | Streptococcus suis | YSIRK_signal~rne~F5_F8_type_C~GH_D~SWM_repeat~F5_F8_type_C |
| 2 | Staphylococcus haemolyticus | YSIRK_signal~MSCRAMM_SdrC~YncE~Herpes_LMP1~PTZ00441 |
| 2 | Dolosigranulum pigrum | YSIRK_signal~rne~MSCRAMM_SdrD~MSCRAMM_SdrC |
| 2 | Staphylococcus gallinarum | YSIRK_signal~PRK08581~bacteriocin_acc~Abhydrolase |
| 2 | Lactobacillus salivarius | YSIRK_signal~PHA03255~MucBP |
| 2 | Lactobacillus salivarius | YSIRK_signal~PRK13914~MucBP |
| 2 | Streptococcus thermophilus | YSIRK_signal~MSCRAMM_SdrD~Trypan_PARP~MucBP |
| 2 | Streptococcus suis | YSIRK_signal~Mac-1~PHA03247~rne |
| 2 | Lactobacillales | YSIRK_signal~Rib~He_PIG |
| 2 | Dolosigranulum pigrum | YSIRK_signal~Glyco_hydro_20b~NAGidase~F5_F8_type_C~Glyco_hydro_20b~GH20_hexosaminidase~F5_F8_type_C |
| 2 | Dolosigranulum pigrum | MSCRAMM_SdrC~YSIRK_signal~LacZ~Big_4~GH20_hexosaminidase~Trypan_PARP |
| 2 | Lactobacillus | MSCRAMM_SdrD~YSIRK_signal~PTZ00121~SLAP |
| 2 | Staphylococcus aureus | YSIRK_signal~Med15~SasC_Mrp_aggreg~DUF1542~PTZ00121~DUF1542~PTZ00108~DUF1542~PRK08581 |
| 2 | Staphylococcus | YSIRK_signal~Med15~SasC_Mrp_aggreg~PTZ00121~DUF1542~PRK08581 |
| 2 | Dolosigranulum pigrum | YSIRK_signal~PTZ00341~Peptidase_M14_like~PRK10199 |
| 2 | Streptococcus suis | YSIRK_signal~PRK13914~GAGBD~G5~PTZ00144~G5 |
| 2 | Dolosigranulum pigrum | YSIRK_signal~PTZ00121~MDN1~GA |
| 2 | Staphylococcus | YSIRK_signal~PRK13108~MSCRAMM_SdrC |
| 2 | Firmicutes | YSIRK_signal~MDN1~Rib |
| 2 | Staphylococcus | MSCRAMM_SdrC~YSIRK_signal~MG1~Herpes_BLLF1~MG1 |
| 2 | Staphylococcus haemolyticus | MSCRAMM_SdrC~YSIRK_signal~PTZ00121~YncE~MSCRAMM_SdrC |
| 2 | Staphylococcus carnosus | YSIRK_signal~DUF2121 |
| 2 | Staphylococcus microti | MSCRAMM_SdrC~YSIRK_signal~MucBP~PTZ00449 |
| 2 | Staphylococcus condimenti | MSCRAMM_SdrC~YSIRK_signal~hyperosmo_Ebh~COG1470 |
| 2 | Staphylococcus capitis | YSIRK_signal~SasC_Mrp_aggreg~DUF1542~PTZ00121~DUF1542~DUF3584~DUF1542~CCDC158~DUF1542 |
| 2 | Lactobacillales | MSCRAMM_SdrD~YSIRK_signal~Herpes_BLLF1~Rib |
| 2 | Staphylococcus | YSIRK_signal~MDN1~LysM |
| 2 | Aerococcus urinae | YSIRK_signal~Glutenin_hmw~RPT_S_cricet~Cornifin |
| 2 | Aerococcus urinae | YSIRK_signal~PRK12323~SdrD_B~Collagen~Glutenin_hmw~RPT_S_cricet~Glutenin_hmw~RPT_S_cricet |
| 2 | Aerococcus urinae | YSIRK_signal~G5~PHA03307~PHA03418 |
| 2 | Aerococcus urinae | YSIRK_signal~SdrD_B~Glutenin_hmw~Collagen~Glutenin_hmw~Collagen~2A1904~Collagen~Glutenin_hmw~Collagen~RPT_S_cricet~Glutenin_hmw~RPT_S_cricet~PTZ00449 |
| 2 | Streptococcus | YSIRK_signal~MSCRAMM_SdrC~FctA~Antigen_C~FctA~Antigen_C~FctA~Antigen_C~FctA |
| 2 | Streptococcus salivarius | YSIRK_signal~MSCRAMM_SdrC~FctA~Antigen_C~FctA~Antigen_C |
| 2 | Streptococcus dysgalactiae | YSIRK_signal~PTZ00341~COG3942~SH3 |
| 2 | Gemella morbillorum | YSIRK_signal~PRK13881 |
| 1 | Streptococcus pneumoniae | YSIRK_signal~Sialidase |
| 1 | Streptococcus sp. C150 | YSIRK_signal~FctA~Antigen_C~FctA~Antigen_C~FctA~Antigen_C~FctA~Antigen_C~FctA~Antigen_C~FctA~Antigen_C~FctA~Antigen_C~FctA~Antigen_C~FctA~Antigen_C~FctA~Antigen_C~FctA~Antigen_C~FctA |
| 1 | Streptococcus pneumoniae | YSIRK_signal~GREB1 |
| 1 | Streptococcus pneumoniae | YSIRK_signal~hyperosmo_Ebh~Pneumo_att_G |
| 1 | Streptococcus mitis | YSIRK_signal~PTZ00121~hyperosmo_Ebh~Rib~PHA03247~repeat_SSSPR51 |
| 1 | Streptococcus salivarius | YSIRK_signal~FctA |
| 1 | Streptococcus uberis | YSIRK_signal~Collagen |
| 1 | Streptococcus suis | YSIRK_signal~GH18_chitinase-like~F5_F8_type_C |
| 1 | Streptococcus sp. DD12 | YSIRK_signal~GH43_62_32_68_117_130 |
| 1 | Streptococcus mitis | YSIRK_signal~Rib~repeat_SSSPR51 |
| 1 | Streptococcus suis | YSIRK_signal~MucBP~repeat_SSSPR51 |
| 1 | Lactobacillus crispatus | YSIRK_signal~SLAP |
| 1 | Streptococcus sp. 'group G' | YSIRK_signal~MreC |
| 1 | Streptococcus sp. 263_SSPC | YSIRK_signal~FctA~Antigen_C~FctA~Antigen_C~FctA |
| 1 | Granulicatella elegans | YSIRK_signal~repeat_SSSPR51 |
| 1 | Streptococcus pyogenes | YSIRK_signal~LCD1 |
| 1 | Finegoldia magna | YSIRK_signal~Flg_new~Rib |
